# Antibodies Raised Against an Aβ Oligomer Mimic Recognize Pathological Features in Alzheimer’s Disease and Associated Amyloid-Disease Brain Tissue

**DOI:** 10.1021/acscentsci.3c00592

**Published:** 2023-12-21

**Authors:** Adam G. Kreutzer, Chelsea Marie T. Parrocha, Sepehr Haerianardakani, Gretchen Guaglianone, Jennifer T. Nguyen, Michelle N. Diab, William Yong, Mari Perez-Rosendahl, Elizabeth Head, James S. Nowick

**Affiliations:** † Department of Chemistry, University of California Irvine, Irvine, California 92697, United States; ‡ Department of Pharmaceutical Sciences, University of California Irvine, Irvine, California 92697, United States; § Department of Pathology and Laboratory Medicine, University of California Irvine, Irvine, California 92697, United States

## Abstract

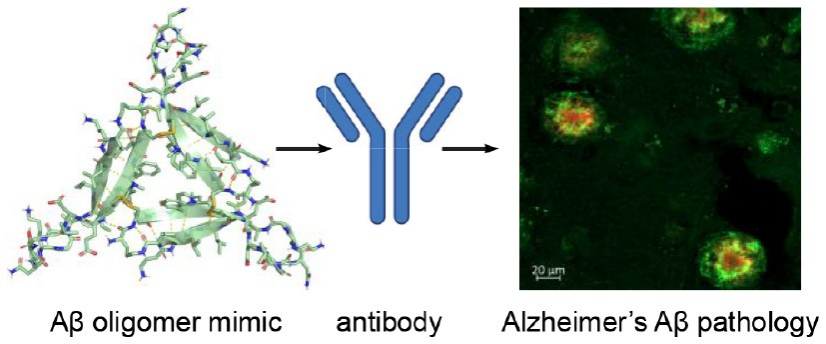

Antibodies that target the β-amyloid peptide (Aβ) and its associated assemblies are important tools in Alzheimer’s disease research and have emerged as promising Alzheimer’s disease therapies. This paper reports the creation and characterization of a triangular Aβ trimer mimic composed of Aβ_17–36_ β-hairpins and the generation and study of polyclonal antibodies raised against the Aβ trimer mimic. The Aβ trimer mimic is covalently stabilized by three disulfide bonds at the corners of the triangular trimer to create a homogeneous oligomer. Structural, biophysical, and cell-based studies demonstrate that the Aβ trimer mimic shares characteristics with oligomers of full-length Aβ. X-ray crystallography elucidates the structure of the trimer and reveals that four copies of the trimer assemble to form a dodecamer. SDS-PAGE, size exclusion chromatography, and dynamic light scattering reveal that the trimer also forms higher-order assemblies in solution. Cell-based toxicity assays show that the trimer elicits LDH release, decreases ATP levels, and activates caspase-3/7 mediated apoptosis. Immunostaining studies on brain slices from people who lived with Alzheimer’s disease and people who lived with Down syndrome reveal that the polyclonal antibodies raised against the Aβ trimer mimic recognize pathological features including different types of Aβ plaques and cerebral amyloid angiopathy.

## Abstract 2

A structurally defined Aβ oligomer mimic is created and studied, and antibodies raised against the Aβ oligomer mimic are used to investigate its relevance to Alzheimer’s disease.

## Introduction

Antibodies are important tools for probing biomolecular species in cells and in tissues. Antibodies are especially valuable, because of their strong affinity and excellent selectivity for peptides and proteins, as well as their ability to be used in highly sensitive fluorescent and luminescent technologies that can identify miniscule quantities of peptides and proteins. Antibodies can also provide insights into the structures and conformations of proteins in cells and in tissues.^1−3^

Antibodies that target monomeric, oligomeric, and fibrillar forms of the β-amyloid peptide (Aβ) are valuable tools for Alzheimer’s disease research and have emerged as Alzheimer’s disease therapies.^4,5^ The anti-Aβ antibody drugs Aducanumab,^6−8^ Lecanemab,^9−11^ and Donanemab^12^ are the first disease-modifying Alzheimer’s disease therapies, with Aducanumab and Lecanemab receiving FDA approval and Donanemab likely to gain future approval. These antibodies act by binding Aβ aggregates and facilitating their clearance from the brain, mitigating both the direct and downstream damaging effects of Aβ, and subsequently slowing cognitive decline.^13,14^ Aducanumab binds a conformational *N*-terminal epitope unique to aggregated forms of Aβ, but not a monomer.^15^ Lecanemab selectively targets Aβ protofibrils^16^ and reduced Aβ protofibrils in the brain and cerebrospinal fluid of Alzheimer’s disease transgenic mice.^17^ Donanemab targets *N*-terminally pyroglutamated Aβ that is aggregated in Aβ plaques.^18^

In Alzheimer’s disease, the Aβ peptide self-assembles to form oligomers and fibrils. Aβ oligomers appear to be important in the pathogenesis and progression of Alzheimer’s disease,^19−37^ with Aβ dimers, trimers, hexamers, and dodecamers as well as larger oligomers identified in Alzheimer’s disease brain tissue.^38−45^ Understanding the structures of Aβ oligomers and Aβ fibrils is crucial for understanding the molecular basis of Alzheimer’s disease and should lead to better diagnostics and therapies for Alzheimer’s disease. The structures of different Aβ *fibril* polymorphs have begun to emerge, owing to advances in cryo-EM and solid-state NMR spectroscopy.^46−56^ In spite of the tremendous advances in amyloid structural biology, the structures of Aβ *oligomers* remain largely unknown.^57^ High-resolution structural elucidation of Aβ oligomers by X-ray crystallography, NMR spectroscopy, or cryo-EM is hindered by challenges in preparing stable, homogeneous Aβ oligomers *in vitro* or isolating sufficient quantities of stable, homogeneous biogenic Aβ oligomers from tissue. These same challenges have also hindered the generation of antibodies against homogeneous structurally defined Aβ oligomers.

The diversity of aggregates that Aβ forms has inspired several approaches for generating Aβ antibodies as tools and probes for identifying Aβ and its many aggregates *in vitro* and in the brain. The 6E10 and 4G8 monoclonal antibodies—among the most extensively used Aβ antibodies in Alzheimer’s disease research—were generated by immunizing mice with a peptide fragment that encompassed the *N*-terminal half of Aβ (Aβ_1–24_).^58,59^ The A11 and OC polyclonal antibodies—among the first “conformation-dependent” Aβ antibodies that distinguished Aβ oligomers and Aβ fibrils—were generated by immunizing rabbits with Aβ_40_ oligomers (A11) or Aβ_42_ fibrils (OC) prepared *in vitro*.^60−62^ These conformation-dependent antibodies have allowed researchers to probe the structures of Aβ oligomers as well as Aβ fibrils in mouse and human brain tissues and fluids.^63−69^ The 1C22 monoclonal antibody—an Aβ antibody that preferentially recognizes Aβ aggregates and not Aβ monomers—was generated by immunizing mice with a disulfide-cross-linked dimer of an Aβ_40_ variant with cysteine in place of Ser_26_.^70−72^ The ACU193 monoclonal antibody—an Aβ antibody that is highly selective for specific types Aβ oligomers—was generated by immunizing mice with Aβ-derived diffusible ligands (ADDLs), a type of Aβ oligomer prepared by aggregating full-length Aβ *in vitro*.^73^ Hundreds of other Aβ antibodies have been raised against various forms of Aβ including Aβ peptide fragments, Aβ oligomers, and Aβ fibrils prepared under different *in vitro* conditions and Aβ isolated from Alzheimer’s disease brains.^74^

The Aβ antigens used to generate Aβ antibodies selective for aggregated forms of Aβ contain a mixture of oligomers or fibrils with inherently diverse epitopes and undefined molecular structures. While antibodies raised against these mixtures can distinguish different aggregation states of Aβ, the lack of high-resolution structural characterization of the Aβ antigens precludes structural correlation of the *in vitro*-prepared oligomers or fibrils with oligomers or fibrils in the brain. Antibodies raised against structurally defined Aβ oligomers, with known high-resolution structures, may help shed light on the structures of the Aβ oligomers that form in the brain or serve as potential immunotherapies for Alzheimer’s disease.

This paper reports the generation and study of antibodies raised against a homogeneous structurally defined triangular trimer derived from Aβ. We first detail the design, synthesis, and X-ray crystallographic structure of the triangular trimer and demonstrate through a series of biophysical and cell-based experiments that the triangular trimer shares many characteristics with oligomers of full-length Aβ. We then describe the generation and study of polyclonal antibodies raised against the triangular trimer. To our knowledge, these are the first antibodies raised against an Aβ-derived oligomer with a known high-resolution structure. We use these antibodies to investigate the relationship between the triangular trimer and Aβ assemblies in postmortem brain tissue from people who lived with Alzheimer’s disease and Down syndrome, as well as brain tissue from 5xFAD transgenic mice.

## Results and Discussion

### Design and Synthesis of the Covalently Stabilized Triangular Trimer 2AT-L

β-Hairpins have emerged as important structural motifs adopted by the Aβ peptide in both the oligomeric and fibrillar state.^75−78^ β-Hairpins are the simplest type of β-sheet, comprising two antiparallel hydrogen-bonded β-strands connected by a loop. Several Aβ β-hairpins have been described in which the central and *C*-terminal regions of the Aβ peptide comprise the β-strands of the β-hairpin.^79,80^ In one example, Härd et al. elucidated the NMR structure of an Aβ_17–36_ β-hairpin bound to an affibody.^81^ In subsequent studies, Härd et al. covalently stabilized Aβ_40_ and Aβ_42_ in a β-hairpin conformation by installing a cross-strand intramolecular disulfide bond and demonstrated that these stabilized Aβ β-hairpins assemble to form soluble oligomers that recapitulate many characteristics of Aβ oligomers.^82,83^

To gain insights into the high-resolution structures of Aβ oligomers, our laboratory has pioneered macrocyclic β-hairpin peptides that mimic Aβ β-hairpins.^84,85^ These β-hairpin peptides contain chemical modifications that stabilize the peptides in a β-hairpin conformation and limit their propensity to aggregate. These modifications enable crystallization and elucidation of the X-ray crystallographic structures of the oligomers that the peptides can form. Using this approach, we have discovered that β-hairpin peptides that mimic Aβ_17–36_ β-hairpins assemble to form triangular trimers that further assemble to form higher-order oligomers, such as hexamers and dodecamers.

**Figure 1:**
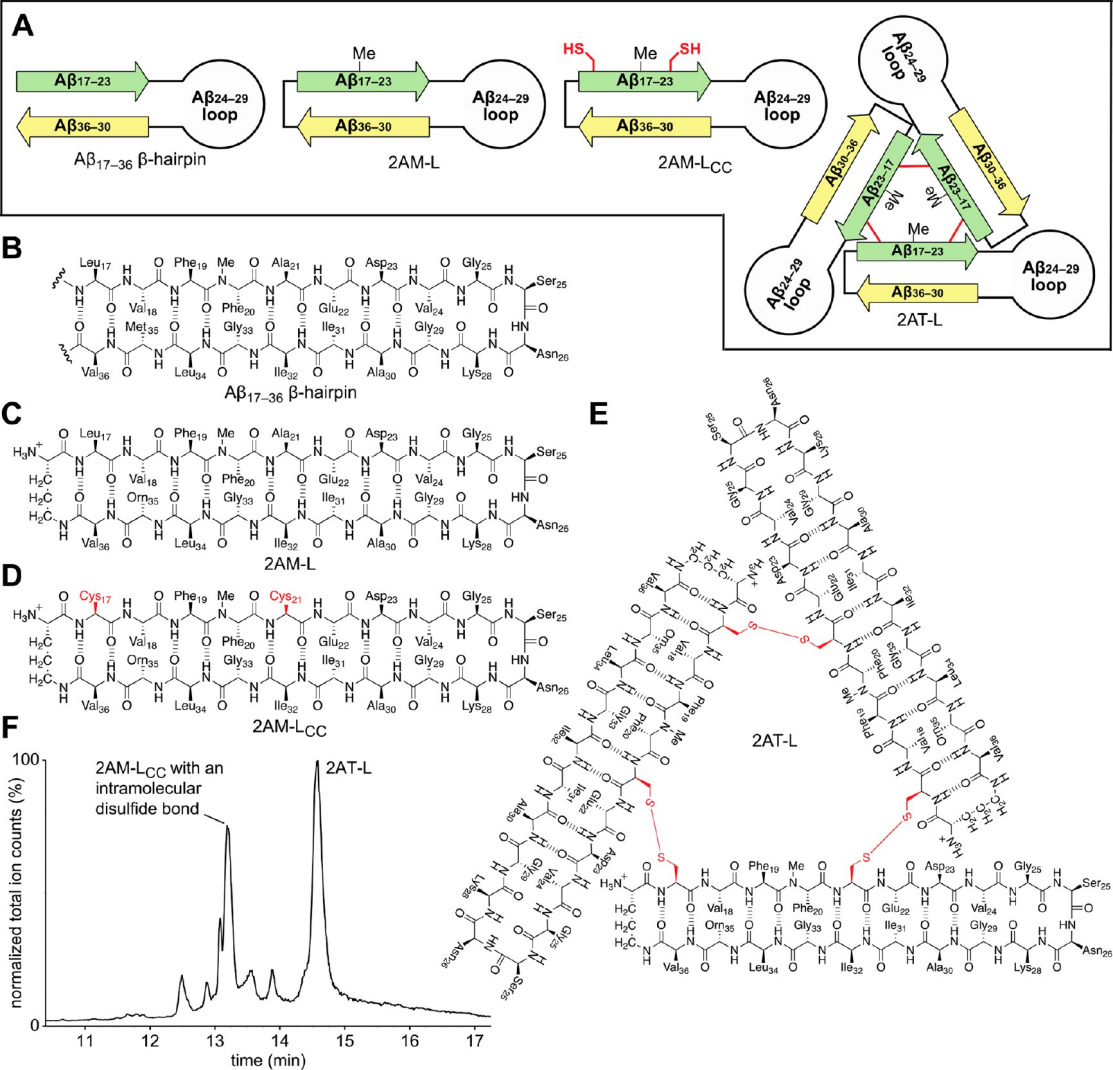
Design and synthesis of the covalently stabilized triangular trimer 2AT-L. (A) Cartoons illustrating the design of 2AM-L, 2AM-L_CC_, and 2AT-L and their relationship to an Aβ_17–36_ β-hairpin. (B–E) Chemical structures of an Aβ_17–36_ β-hairpin, 2AM-L, 2AM-L_CC_, and 2AT-L. (F) LC-MS trace of the oxidation reaction mixture of 2AM-L_CC_ to form 2AT-L after 48 h in 20% DMSO with triethylamine. The two major products that form during the oxidation reaction are indicated on the trace—the desired species 2AT-L and 2AM-L_CC_ with an intramolecular disulfide bond.

We designed peptide 2AM-L to mimic an Aβ_17–36_ β-hairpin (Figure 1A–C). 2AM-L contains a δ-linked ornithine turn unit that connects the N- and C-termini of the peptide and helps enforce a β-hairpin conformation. To improve solubility of the peptide and prevent uncontrolled aggregation, 2AM-L also contains an *N*-methyl group on the amide backbone of Phe_20_ and the charged isostere of methionine, ornithine, at position 35. Previous X-ray crystallographic studies of three closely related peptide analogues of 2AM-L revealed that these peptides assemble to form triangular trimers (Figure S1). While these 2AM-L analogues assemble to form triangular trimers at high concentrations of X-ray crystallography (>1 mM), these analogues and 2AM-L do not appear to form a triangular trimer at low, more biologically meaningful concentrations (<50 μM). For this reason, covalent stabilization of the triangular trimer is needed to study its structural, biophysical, and biological properties.^86,87^ Covalent stabilization of the triangular trimer also ensures oligomer homogeneity by eliminating the monomer–oligomer equilibrium that would occur for monomers that assemble to form trimers or other oligomers.

We designed 2AT-L as a covalently stabilized analogue of a triangular trimer formed by 2AM-L (Figure 1A and E). The design of 2AT-L is based on the previously reported X-ray crystallographic structures of triangular trimers composed of β-hairpin peptides derived from Aβ_17–36_ (Figure S1).^88−90^ At the three corners of these triangular trimers, Leu_17_ of one monomer subunit is near Ala_21_ of an adjacent monomer subunit. To stabilize 2AM-L into a triangular trimer, we mutated Leu_17_ and Ala_21_ to cysteine to create 2AM-L_CC_ (Figure 1A and D). Oxidation of 2AM-L_CC_ in aqueous DMSO with triethylamine (TEA) generates 2AT-L. LC-MS analysis of the oxidation reaction mixture shows that 2AM-L_CC_ cross-links to form two major products—2AT-L and 2AM-L_CC_ with an intramolecular disulfide bond (Figure 1F). 2AT-L is isolated from the crude reaction mixture using reverse-phase HPLC. Oxidation of ∼30 mg of 2AM-L_CC_ typically yields ∼8–10 mg 2AT-L of >98% purity.

### X-Ray Crystallographic Structure of 2AT-L

**Figure 2:**
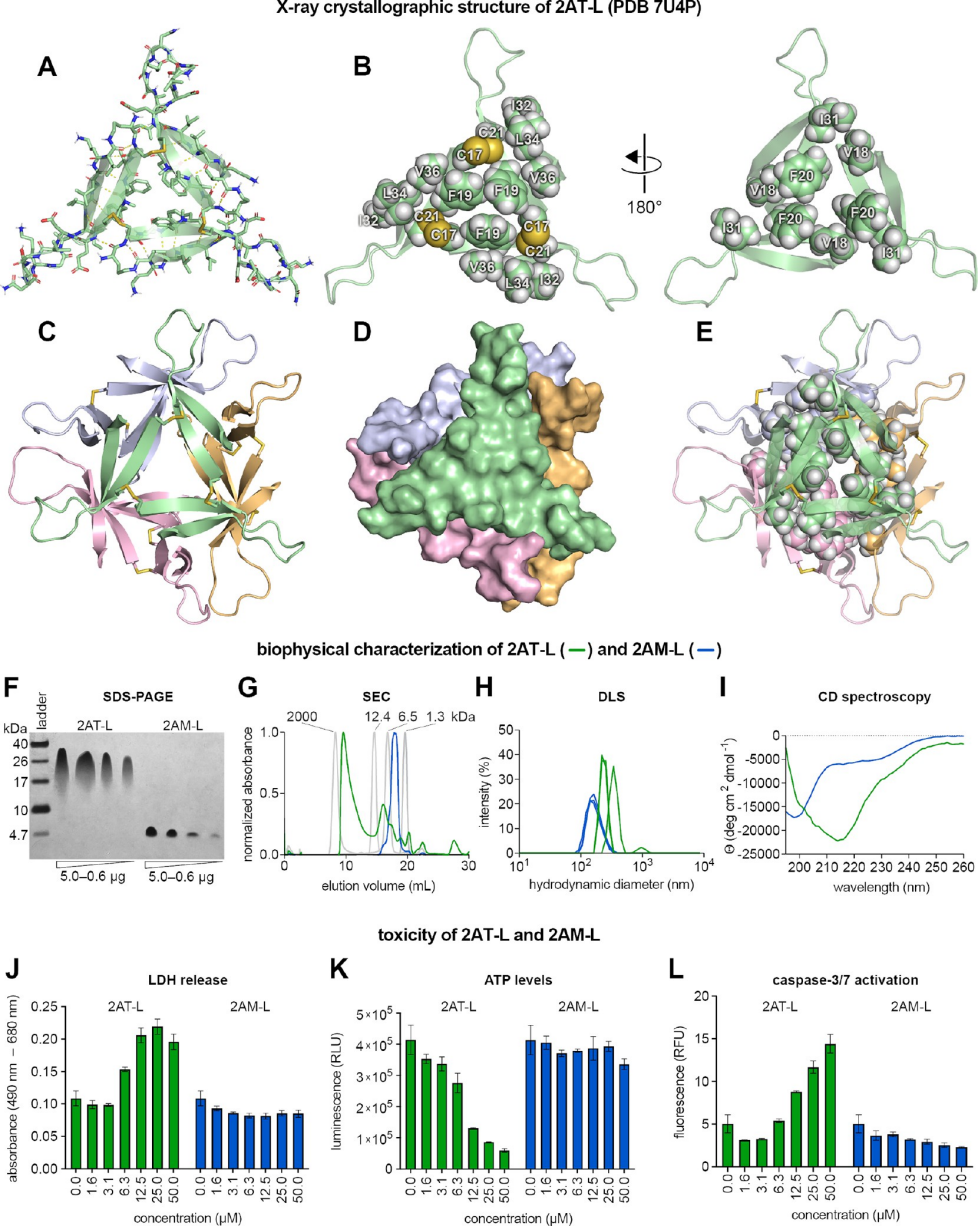
Structural, biophysical, and cell-based toxicity studies of 2AT-L. (A) X-ray crystallographic structure of 2AT-L illustrating the three folded Aβ_17–36_ β-hairpins that comprise 2AT-L (PDB 7U4P). (B) Cartoon and sphere models of 2AT-L illustrating the two hydrophobic surfaces of 2AT-L and the hydrophilic loops that extend off the core of the trimer. (C) X-ray crystallographic structure of the ball-shaped dodecamer formed by four copies of 2AT-L. (D) Surface rendering of the ball-shaped dodecamer formed by 2AT-L illustrating how the four trimers fit together to form the dodecamer. (E) Cartoon and sphere model of the ball-shaped dodecamer formed by 2AT-L illustrating the hydrophobic core formed by Val_18_, Phe_20_, and Ile_31_ at the center of the dodecamer. (F) Silver stained SDS-PAGE of varying amounts of 2AT-L and 2AM-L. SDS-PAGE was performed in Tris buffer at pH 6.8 with 2% (w/v) SDS. (G) SEC chromatograms of 2AT-L and 2AM-L. SEC was performed on 1.0-mg/mL solutions of 2AT-L and 2AM-L in 50 mM Tris buffer (pH 8.0) with 150 mM NaCl using a Superdex 75 10/300 column. Dextran blue (2000 kDa), cytochrome C (12.4 kDa), aprotinin (6.5 kDa), and vitamin B_12_ (1.3 kDa) were run as size standards. (H) DLS traces of 2AT-L and 2AM-L. DLS traces were acquired on a 25 μM solution of 2AT-L and a 75 μM solution of 2AM-L in 10 mM phosphate buffer at pH 7.4 after centrifugation at 16 000*g* for 5 min. (I) CD spectra of 2AT-L and 2AM-L. CD spectra were acquired on a 25-μM solution of 2AT-L and a 75-μM solution of 2AM-L in 10 mM phosphate buffer at pH 7.4. (J) LDH release assay of 2AT-L and 2AM-L. (K) CellTiter-Glo ATP assay of 2AT-L and 2AM-L. (L) Caspase-3/7 activation assay of 2AT-L and 2AM-L. The assays in J–L were performed by exposing SH-SY5Y cells (30 000 cells/well on a black-walled half area 96-well plate) to a 2-fold dilution series of 2AT-L and 2AM-L (50 μM to 1.6 μM) for 72 h. Each assay was performed according to the manufacturer’s instructions. Data from these assays are shown as the mean of three technical replicates, with error bars representing the standard deviation.

We determined the X-ray crystallographic structure of 2AT-L at 1.8-Å resolution (PDB 7U4P). The X-ray crystallographic structure reveals that 2AT-L is composed of three folded β-hairpins that are cross-linked together in the envisioned manner, in which Cys_17_ on one monomer forms a disulfide bond with Cys_21_ of the adjacent monomer at each corner (Figure 2A). The Aβ_17–23_ and Aβ_30–36_ β-strands of the three β-hairpins that comprise 2AT-L consist mainly of residues from the hydrophobic central and *C*-terminal regions of Aβ, creating two hydrophobic surfaces on 2AT-L (Figure 2B). The three hydrophilic Aβ_24–29_ loops extend off the hydrophobic core of 2AT-L.

In the crystal lattice, four copies of 2AT-L assemble to form a ball-shaped dodecamer (Figure 2C and D). The dodecamer is stabilized by an edge-to-edge hydrogen-bonding network between the backbones of adjacent trimers and by hydrophobic packing at the core of the dodecamer between the surfaces of the trimers that contain Val_18_, Phe_20_, and Ile_31_ (Figure 2E). In total, the dodecamer contains 34 intermolecular hydrogen bonds between the four copies of 2AT-L, and the core is packed with 36 hydrophobic amino acid side chains. The outer surface of the dodecamer displays the hydrophobic amino acids on the other surface of the trimer—Phe_19_, Ile_32_, Leu_34_, and Val_36_, as well as the disulfide bond between Cys_17_ and Cys_21_. In the crystal lattice, six dodecamers pack together to form an annular pore-like structure (Figure S2). The annular pore-like structure is stabilized by hydrophobic packing between the outer surfaces of adjacent dodecamers and contacts between the loops. The propensity to form dodecamers that further assemble into pore-like structures appears to be a common characteristic of triangular trimers derived from Aβ_17–36_, as we have observed similar dodecameric assemblies in previous studies.^86−90^

### Biophysical Studies of 2AT-L

To investigate the structure and assembly of 2AT-L in solution, we turned to SDS-PAGE, size exclusion chromatography (SEC), dynamic light scattering (DLS), and circular dichroism (CD) spectroscopy. For SDS-PAGE, we loaded varying amounts (5.0, 2.5, 1.25, and 0.6 μg) of either 2AT-L or 2AM-L in each lane. 2AT-L migrates as comet-shaped bands between the 40- and 26-kDa molecular weight markers (Figure 2F). The positions of these bands indicate that the 2AT-L (6.6 kDa) forms higher-order assemblies. At low loading (0.6 μg), 2AT-L migrates at the 26-kDa molecular weight marker, which is consistent with the molecular weight of a dodecamer composed of four copies of 2AT-L (∼26.4 kDa). The band streaks downward, indicating that under the conditions of SDS-PAGE the dodecamer is in equilibrium with smaller assemblies, such as hexamers and nonamers. At higher loadings of 2AT-L, the bands migrate at molecular weights larger than 26 kDa, suggesting that additional 2AT-L trimers may be bound to the dodecamer. 2AM-L migrates at or below the 4.7-kDa molecular weight marker and above the 1.7-kDa marker (Figure S3), which is consistent with the molecular weight of a monomer (2.1 kDa) or dimer (4.2 kDa). The assembly of 2AT-L to form a dodecamer in SDS-PAGE is consistent with the observation of the ball-shaped dodecamer in the crystal lattice of 2AT-L, suggesting that the ball-shaped dodecamer is the actual assembly that 2AT-L forms in a membrane-like environment and is not merely an artifact of crystal lattice formation.

To investigate the assembly of 2AT-L in an aqueous environment in the absence of SDS, we used SEC and DLS. For SEC, we ran 2AT-L on a Superdex 75 column and eluted with TBS (50 mM Tris buffer at pH 8.0 with 150 mM NaCl). Under these conditions, 2AT-L elutes as two major peaks, with the most predominant of the two peaks eluting at 9.6 mL and the other peak eluting at 16.3 mL (Figure 2G). The 9.6-mL peak elutes between the 132.8 kDa and 66.4 kDa size standards, indicating that 2AT-L assembles to form large species of ca. 100 kDa, well above the 26-kDa size of the dodecamer (Figure S4). The 16.3-mL peak elutes between the 12.4-kDa and 6.5-kDa size standards, which is consistent with the molecular weight of 2AT-L itself. Investigation of 2AT-L using DLS shows that in phosphate buffer (10 mM sodium phosphate at pH 7.4) 2AT-L forms large species with hydrodynamic diameters of ca. 300 nm (Figure 2H). The SDS-PAGE, SEC, and DLS experiments support an assembly model where, in aqueous solution, 2AT-L aggregates to form large species, and SDS dissociates these large species into their component parts, which appear to be dodecamers. Our working model is that these large 2AT-L species assemble similarly to how the dodecamers pack together in the crystal lattice, where the outer surfaces of the trimer subunits of adjacent dodecamers pack together, with additional contacts between loops.

In SEC, 2AM-L elutes between the 1.3-kDa and 6.5-kDa size standards, which is consistent with the molecular weight of a monomer or dimer (Figure 2G). In contrast, in DLS, 2AM-L forms large species with hydrodynamic diameters of ca. 150 nm (Figure 2H). The different assembly properties of 2AM-L in SEC and DLS might be explained by differences in these techniques—SEC is performed under flowing conditions through a gel matrix, which may cause sheering, whereas DLS is performed in a still solution with no matrix.

To better understand the structures of 2AT-L and the higher-order assemblies formed by 2AT-L in solution, we used CD spectroscopy. In phosphate buffer, the CD spectrum of 2AT-L shows a minimum centered at 218 nm, which is characteristic of β-hairpins (Figure 2I).^91−93^ In contrast, the CD spectrum of 2AM-L shows a minimum near 200 nm, with shallow negative ellipticity from ca. 210 to 240 nm, which suggests a random coil structure. These data support a structural model in which 2AT-L and the higher-order assemblies formed by 2AT-L are composed of folded β-hairpins and that 2AM-L does not fold to form a β-hairpin. These contrasting behaviors of 2AT-L and 2AM-L demonstrate the cooperativity between folding and assembly often observed for amyloidogenic peptides and proteins.^84,85^ Furthermore, the CD data suggest that, in solution, the component β-hairpin peptides of 2AT-L adopt the folded conformation observed in the X-ray crystallographic structure of 2AT-L.

The structural and biophysical studies described above demonstrate similarities between 2AT-L and oligomers of full length Aβ. 2AT-L assembles in the crystal lattice and in the membrane-like environment of SDS micelles to form a dodecamer and forms large higher-order assemblies with molecular weights of ca. 10^2^–10^3^ kDa in the absence of SDS. SDS-stable Aβ dodecamers composed of antiparallel β-sheets have been observed in protein extracts from mouse and human brains,^41,42,129^ and the large assemblies formed by 2AT-L in the aqueous environments of SEC and DLS recapitulate previously observed large assemblies of full-length Aβ.^39,94,95^ While we do not know the exact structures of the dodecamer and higher-order assemblies formed by 2AT-L in solution, our working model is that the dodecamer observed in SDS-PAGE is similar to the dodecamer observed crystallographically and that the dodecamer is a building block of the higher-order assemblies. It is also possible that the dodecamer and higher-order assemblies formed in solution do not resemble the higher-order assemblies observed crystallographically.

### Cell-Based Toxicity Studies of 2AT-L

Oligomers of full-length Aβ are toxic toward cells in culture.^35,37^ To determine if 2AT-L is also toxic, we exposed the human neuroblastoma cell line SH-SY5Y to 2AT-L and assessed three different metrics of toxicity: LDH release, ATP reduction, and caspase-3/7 activation. In each of the three assays, we first exposed SH-SY5Y cells to varying concentrations of 2AT-L or 2AM-L (0–50 μM) for 72 h before performing the assay. The three toxicity metrics indicate that 2AT-L is toxic toward SH-SY5Y cells in a dose-dependent manner (Figure 2J–L). Exposing the SH-SY5Y cells to 2AT-L increased LDH release and reduced ATP levels at concentrations as low as 6.3 μM and activated caspase-3/7 at concentrations as low as 12.5 μM. In contrast, exposing SH-SY5Y cells to the monomer 2AM-L caused little to no change in any of the three toxicity markers at concentrations up to 50 μM, which is equivalent to 16.7 μM of the trimer 2AT-L.

These toxicity studies further demonstrate similarities between 2AT-L and oligomers of full-length Aβ. Like oligomers of full-length Aβ, 2AT-L is toxic toward cells in culture, eliciting toxicity by interacting with the cells and promoting membrane disruption and release of LDH, depleting ATP, and activating caspase-3/7-mediated apoptosis. Our laboratory has previously shown that full-length Aβ also promotes LDH release, ATP depletion, and caspase-3/7 activation.^87^

### Generation and in Vitro Characterization of a Polyclonal Antibody against 2AT-L

While the structural, biophysical, and cell-based studies described above show that 2AT-L behaves like an Aβ oligomer, these studies do not on their own establish a relationship between 2AT-L and biogenic assemblies of full-length Aβ formed in the brain. To investigate the relationship between 2AT-L and Aβ assemblies that form in the brain, we generated a polyclonal antibody (pAb) against 2AT-L (pAb_2AT-L_) and then examined the immunoreactivity of this antibody with postmortem brain tissue from people who lived with Alzheimer’s disease and people who lived with Down syndrome, as well as brain tissue from 5xFAD transgenic mice. The goal of these studies was to determine if antibodies raised against the synthetic Aβ oligomer model 2AT-L recognize biogenic Aβ assemblies and thus provide evidence that 2AT-L may share structural or conformational epitopes with assemblies of full-length Aβ.

To generate pAb_2AT-L_, 2AT-L was first conjugated to the carrier protein keyhole limpet hemocyanin (KLH), and then rabbits were immunized with the trimer-KLH conjugate in Freunds adjuvant. Antibody titers in the rabbits reached high levels after two immunizations and remained high with repeated boosts over the course of the immunization schedule. We purified pAb_2AT-L_ from rabbit blood plasma by affinity chromatography using 2AT-L conjugated to NHS-activated agarose. The affinity-purified pAb_2AT-L_ was used in all subsequent studies.

**Figure 3:**
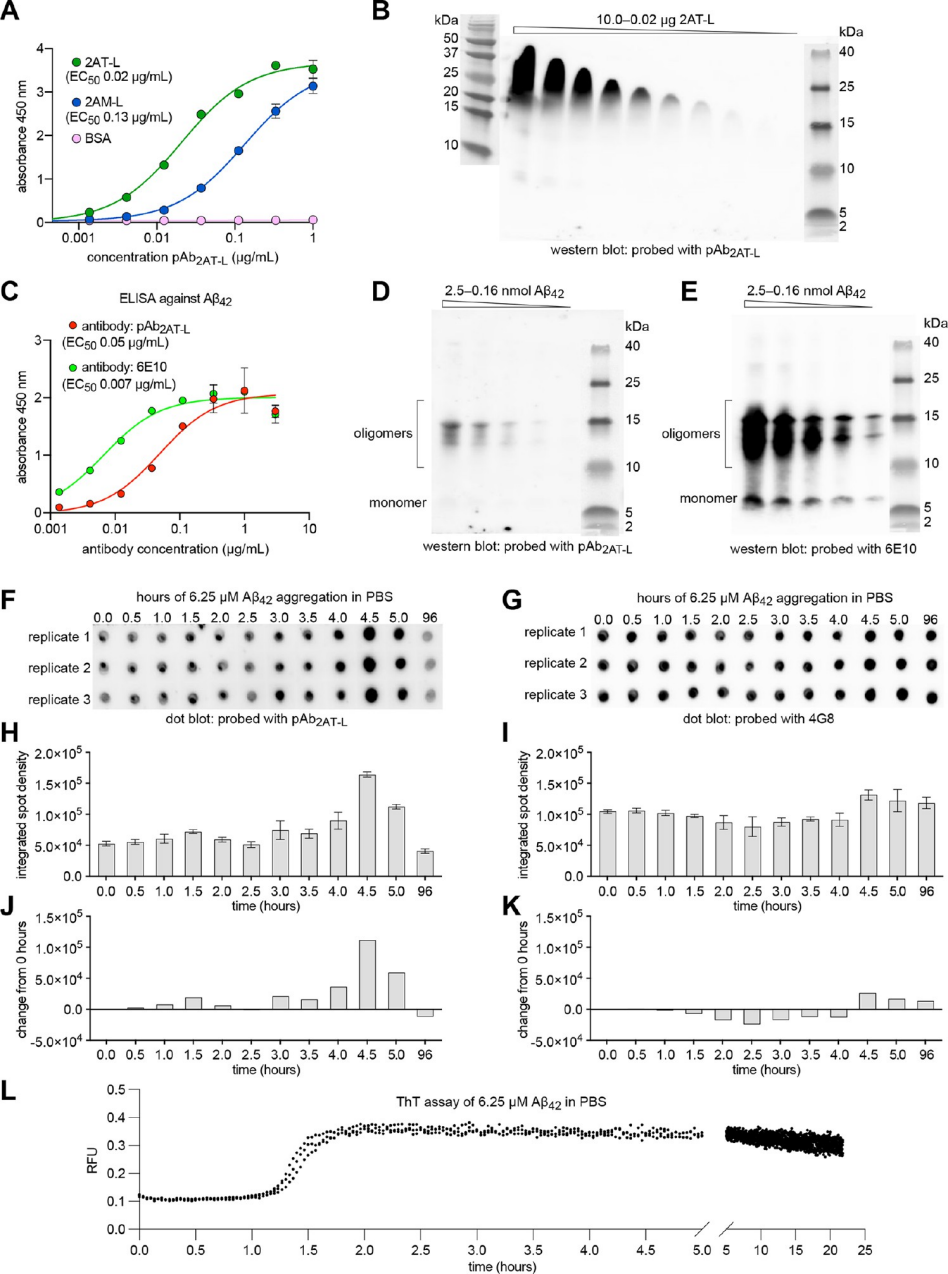
*In vitro* characterization of pAb_2AT-L_ with 2AT-L and 2AM-L and Aβ_42_. (A) Indirect ELISA of pAb_2AT-L_ against 2AT-L, 2AM-L, and BSA. (B) Western blot analysis of pAb_2AT-L_ against a concentration gradient of 2AT-L. SDS-PAGE was performed on a 16.5% Tris-Tricine gel (Bio-Rad); 10 μL was loaded in each lane. (C) Indirect ELISA of pAb_2AT-L_ and 6E10 against Aβ_42_. (D and E) Western blot analysis of pAb_2AT-L_ (D) and 6E10 (E) against a concentration gradient of Aβ_42_. SDS-PAGE was performed on a 16.5% Tris-Tricine gel (Bio-Rad); 10 μL was loaded in each lane. (F and G) Dot blot analysis of pAb_2AT-L_ (F) and 4G8 (G) against 6.25 μM Aβ_42_ aggregated over time in PBS. A 1 μL portion of the Aβ_42_ solution was spotted in triplicate on 0.2 μm nitrocellulose membranes every 30 min for 5 h and then at 96 h. (H and I) Average integrated spot density from the dot blots in F and G. Integrated density for each spot was determined using ImageJ. (J and K) Change in integrated spot density from the 0-h time point. (L) ThT aggregation assay of 6.25 μM Aβ_42_. The ThT assay was performed at 25 °C under quiescent conditions in PBS (10 mM Na_2_HPO_4_, 1.8 mM KH_2_PO_4_, 137 mM NaCl, 2.7 mM KCl) at pH 7.4 containing 10 μM ThT. ThT fluorescence was monitored at 440 nm excitation and 485 nm emission.

The Aβ oligomer model 2AT-L has unique conformations, multivalency, and structures that are not present on the monomer 2AM-L; conversely, 2AT-L shares significant sequence homology with 2AM-L. Thus, 2AT-L displays unique epitopes that are not present on 2AM-L, as well as epitopes that are not unique and are present on 2AM-L. To investigate the selectivity of pAb_2AT-L_ for epitopes that are unique to 2AT-L, we compared the binding of pAb_2AT-L_ to 2AT-L and the corresponding monomer 2AM-L using an indirect ELISA. In this ELISA experiment, each well of a 96-well plate was treated with 50 ng of either 2AT-L or 2AM-L, or 1% bovine serum albumin (BSA) as a negative control. A 3-fold dilution series of pAb_2AT-L_ was then applied to the wells, followed by an HRP-conjugated antirabbit IgG secondary antibody. The ELISA showed that pAb_2AT-L_ binds 2AT-L with a half-maximal effective concentration (EC_50_) of 0.02 μg/mL, while it only binds 2AM-L with an EC_50_ of 0.13 μg/mL (Figure 3A). Thus, pAb_2AT-L_ is 6.5-fold more selective for 2AT-L than for 2AM-L. The greater selectivity for 2AT-L demonstrates that pAb_2AT-L_ is more selective for epitopes unique to the triangular trimer 2AT-L than epitopes shared by 2AT-L and the monomer 2AM-L. Western blot analysis shows that pAb_2AT-L_ recognizes epitopes on the higher-order assemblies formed by 2AT-L in SDS-PAGE and further illustrates the concentration dependence of higher-order assembly of 2AT-L (Figure 3B).

To investigate the immunoreactivity of pAb_2AT-L_ with full-length Aβ, we turned to ELISA and Western blot analysis. For the ELISA, each well of a 96-well plate was treated with a 1 μM solution of Aβ_42_ to coat the wells with Aβ_42_. A 3-fold dilution series of pAb_2AT-L_ or the anti-Aβ antibody 6E10 was then applied to the wells, followed by an appropriate HRP-conjugated secondary antibody. The ELISA shows that pAb_2AT-L_ binds Aβ_42_ with an EC_50_ of 0.05 μg/mL and 6E10 binds Aβ_42_ with an EC_50_ of 0.007 μg/mL (Figure 3C). For the Western blot analysis, we prepared a 2-fold dilution series of Aβ_42_ (0.25–0.016 mM) in 1× Tricine Sample Buffer (Bio-Rad) and ran 10 μL of each solution through a 16.5% Tris-Tricine Gel (Bio-Rad). The gel was then transferred to a 0.2 μm nitrocellulose membrane, and standard immunoblotting procedures were performed with pAb_2AT-L_ or 6E10. The Western blot shows that under the conditions of SDS-PAGE, Aβ_42_ forms a mixture of oligomers as well as a monomer. The predominant Aβ_42_ oligomers observed on SDS-PAGE migrate at a molecular weight consistent with a trimer and tetramer. While 6E10 recognizes the oligomers and the monomer (Figure 3E), pAb_2AT-L_ appears to primarily recognize the oligomers, exhibiting little or no recognition of the monomer bands (Figure 3D). The ELISA and Western blot demonstrate that pAb_2AT-L_ binds Aβ_42_*in vitro* with less affinity than 6E10 and with greater selectivity for Aβ_42_ oligomers formed under the conditions of SDS-PAGE. These findings suggest that some of the Aβ_42_ oligomers formed under the conditions of SDS-PAGE might share conformational or structural similarities with 2AT-L.

To better understand the selectivity of pAb_2AT-L_ for aggregated forms of Aβ_42_, we performed a dot blot assay in which we examined the immunoreactivity of pAb_2AT-L_ and the anti-Aβ antibody 4G8 with Aβ_42_ aggregated over time. In this experiment, we aggregated 6.25 μM Aβ_42_ in PBS and spotted 1 μL portions of the Aβ_42_ solution every 30 min over a 5-h period, and again after 96 h, which constitutes mature fibrils. (A thioflavin T (ThT) assay of 6.25 μM Aβ_42_ in PBS shows that under these conditions Aβ_42_ begins to form ThT-reactive aggregates after 1 h, which plateau after 1.75 h (Figure 3L).) We then performed standard immunoblotting with pAb_2AT-L_ and 4G8 and quantified the integrated density of each spot on the dot blot using ImageJ. The dot blot assay shows that pAb_2AT-L_ immunoreactivity with Aβ_42_ substantially increases between 4.0 and 5.0 h (Figure 3F, H, and J), whereas 4G8 immunoreactivity remains relatively constant (Figure 3G, I, and K). pAb_2AT-L_ exhibits less immunoreactivity with the mature fibrils at 96 h than the early aggregates formed over the first 5 h of aggregation. In contrast, 4G8 exhibits comparable immunoreactivity with the mature fibrils at 96 h and the early aggregates. The results from the dot blot assays suggest that pAb_2AT-L_ exhibits some selectivity for conformational or structural epitopes that form during Aβ_42_ aggregation *in vitro* and that these epitopes are more prevalent on early Aβ_42_ aggregates than mature Aβ_42_ fibrils. Furthermore, these findings suggest that these epitopes might share conformational or structural similarities with 2AT-L.

### Immunoreactivity of pAb_2AT-L_ with Brain Tissue from People Who Lived with Alzheimer’s Disease and People Who Lived with Down Syndrome

Accumulation of Aβ is etiologically associated with Alzheimer’s disease and other amyloid-related diseases.^96^ In individuals with late-onset Alzheimer’s disease (LOAD)—the most common form of the disease—Aβ oligomer levels begin to rise, and plaque deposition typically starts about two decades before the onset of symptoms and continues throughout the disease.^42,97−100^ Individuals with trisomy 21 (Down syndrome) have an additional copy of the *APP* gene, which encodes the amyloid precursor protein from which Aβ is cleaved. As a result, Aβ accumulation and subsequent plaque formation occurs much earlier in individuals with trisomy 21, with almost all having plaque pathology by 40 years of age, and many Down syndrome Alzheimer’s disease (DSAD) individuals showing clinical signs of dementia after 50 years of age.^101−104^ In individuals with cerebral amyloid angiopathy (CAA), another neuropathology often associated with Alzheimer’s disease, Aβ assemblies accumulate around arterioles and capillaries in the cerebral cortex.^105−107^ Although CAA and Alzheimer’s disease can occur independently, the deposition of Aβ in CAA is thought to occur concurrently with Aβ plaque deposition and to contribute to dementia in Alzheimer’s disease.^108^

To explore the relationship between the trimer 2AT-L and biogenic Aβ assemblies formed in brains from people with Alzheimer’s disease, we performed immunohistochemical experiments with pAb_2AT-L_ on clinically characterized brain tissue from elderly LOAD individuals, younger DSAD individuals, and elderly LOAD individuals with CAA. Table 1 summarizes the demographics of each individual.

**Table 1:** Individual Demographics^a^

		age	sex	PMI (hours)	NPDx1	tangle stage	plaque stage
LOAD individuals	1	90	F	6.58	Alzheimer’s disease	stage 6	stage C
	2	90	M	4.17	Alzheimer’s disease	stage 6	stage C
	3	89	F	4.08	Alzheimer’s disease	stage 6	stage C
DSAD individuals	1	47	F	6.5	trisomy 21 (AD present)	stage 6	stage C
	2	57	M	4.25	trisomy 21 (AD present)	stage 6	stage C
	3	49	M	6.03	trisomy 21 (AD present)	stage 6	stage C
	4	55	F	4.58	trisomy 21 (AD present)	stage 6	stage C
CAA individual	1	90	M	3.75	Alzheimer’s disease	stage 6	stage B

a Abbreviations: postmortem interval (PMI), neuropathological diagnosis (NPDx1), Alzheimer’s disease (AD).

**Figure 4:**
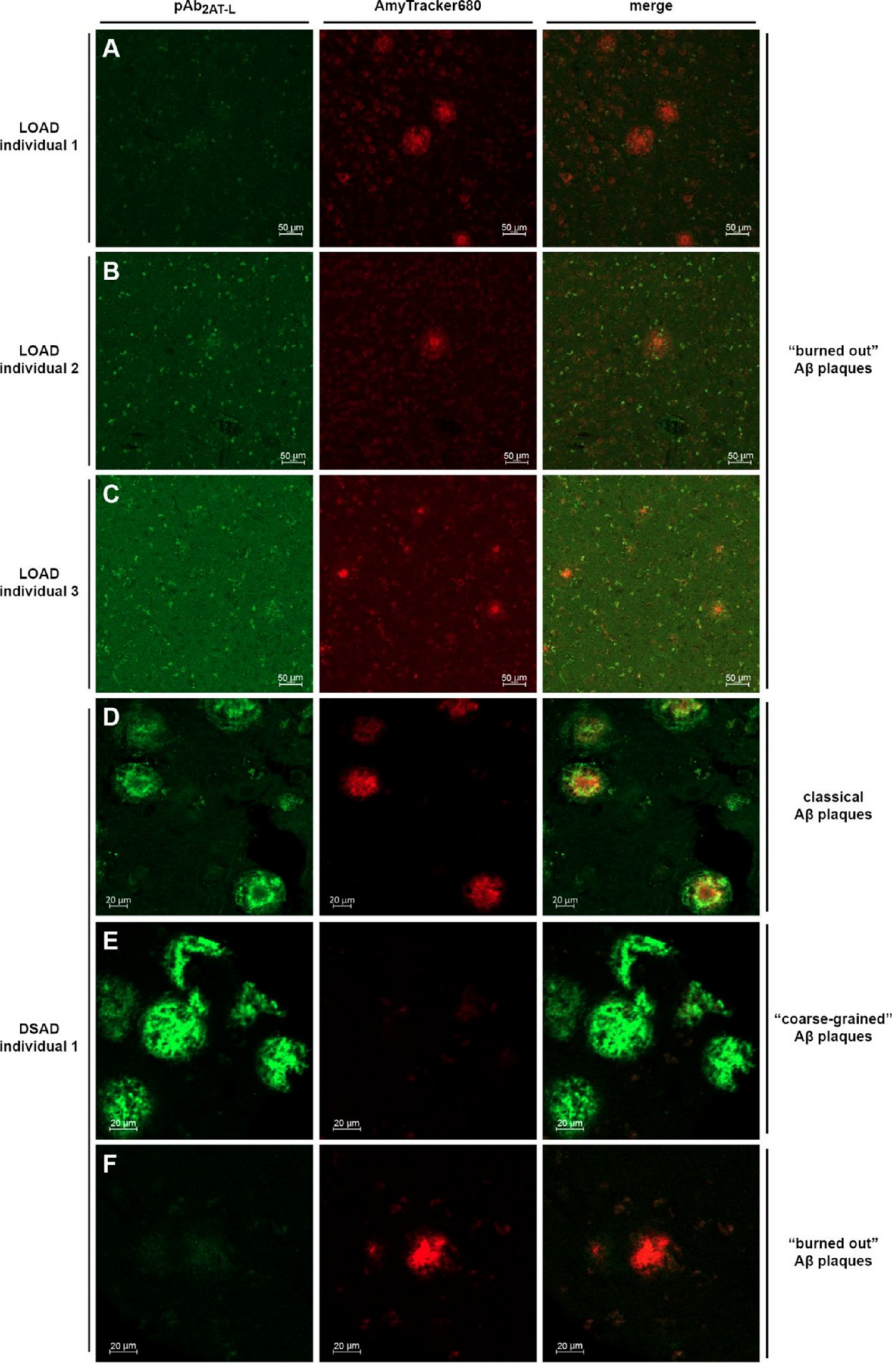
Confocal fluorescence micrographs of LOAD and DSAD frontal cortex brain tissue stained with pAb_2AT-L_ (green) and AmyTracker680 (red). (A–C) Representative images (10× objective) of plaques in frontal cortex brain slices from people who lived with late-onset Alzheimer’s disease (LOAD). (D–F) Representative images (20× objective) of classical Aβ plaques (D), “coarse-grained” plaques (E), and “burned-out” plaques (F) in a frontal cortex brain slice from a DSAD individual.

To investigate the immunoreactivity of pAb_2AT-L_ with Aβ plaques from LOAD individuals, we stained brain slices from each LOAD individual with pAb_2AT-L_ and AmyTracker680 and then imaged the brain slices using confocal fluorescence microscopy. Although AmyTracker680 stained the dense cores of the plaques, no significant pAb_2AT-L_ staining was observed in or around the plaques, even after imaging at a higher laser power (Figures 4A–C and S5 and S6). These plaques correspond to “burned-out” plaques, which are composed of only dense cores and lack the diffuse Aβ around the cores and are thought to have once been neuritic plaques.^109−111^

To investigate the immunoreactivity of pAb_2AT-L_ with Aβ plaques from the DSAD individuals, we stained a brain slice from DSAD individual 1 with pAb_2AT-L_ and AmyTracker680 and brain slices from DSAD individuals 2–4 with only pAb_2AT-L_. Confocal fluorescence microscopy reveals that pAb_2AT-L_ strongly stains plaques in the brain slices from DSAD individual 1 (Figure 4D–F) and DSAD individuals 2–4 (Figures S7–S11). Three distinct plaque types that exhibit different immunohistochemical and chemical staining properties were observed in DSAD individual 1: plaques that are stained by both pAb_2AT-L_ and AmyTracker680, plaques that are only stained by AmyTracker680, and plaques that are only stained by pAb_2AT-L_.

The observation of these different plaque types is consistent with previous immunohistochemical and chemical staining studies in DSAD brain tissue slices.^112^ The plaques that are stained by both pAb_2AT-L_ and AmyTracker680 correspond to classical Aβ plaques (Figure 4D).^113^ Classical Aβ plaques are characterized by a dense Aβ fibrillar core surrounded by more diffuse Aβ deposits that are thought to be nonfibrillar.^61,114−118^ These classical Aβ plaques show the strongest pAb_2AT-L_ staining around the peripheries of the dense cores and weaker staining of the diffuse Aβ around the dense cores, with little or no overlap of pAb_2AT-L_ and AmyTracker680 staining. The plaques that are only stained by pAb_2AT-L_ correspond to diffuse “coarse-grained” plaques, which are associated with early onset forms of Alzheimer’s disease and are common in DSAD pathology (Figure 4E).^110,119^ The plaques that are only stained by AmyTracker680 correspond to “burned-out” dense-core plaques (Figure 4F).

The brain slices from the LOAD and DSAD individuals exhibited markedly different plaque pathologies and staining properties, an observation consistent with previous studies of LOAD and DSAD brain tissue.^112^ The LOAD tissues almost exclusively contained end-stage burned-out plaques, composed of only dense cores, which were not stained by pAb_2AT-L_. In contrast, the DSAD tissue contained multiple plaque types, many of which were strongly stained by pAb_2AT-L_. Importantly, the differences in staining between the LOAD and DSAD tissues likely does *not* reflect a preference of pAb_2AT-L_ for binding plaques in DSAD tissue over plaques in LOAD tissue but, rather, likely reflects that the DSAD tissue contains more diffuse Aβ plaques than the LOAD tissues, an observation consistent with previous studies on brain tissue from people who lived with early onset Alzheimer’s disease and people who lived without cognitive impairment.^110,112,119−122^

**Figure 5:**
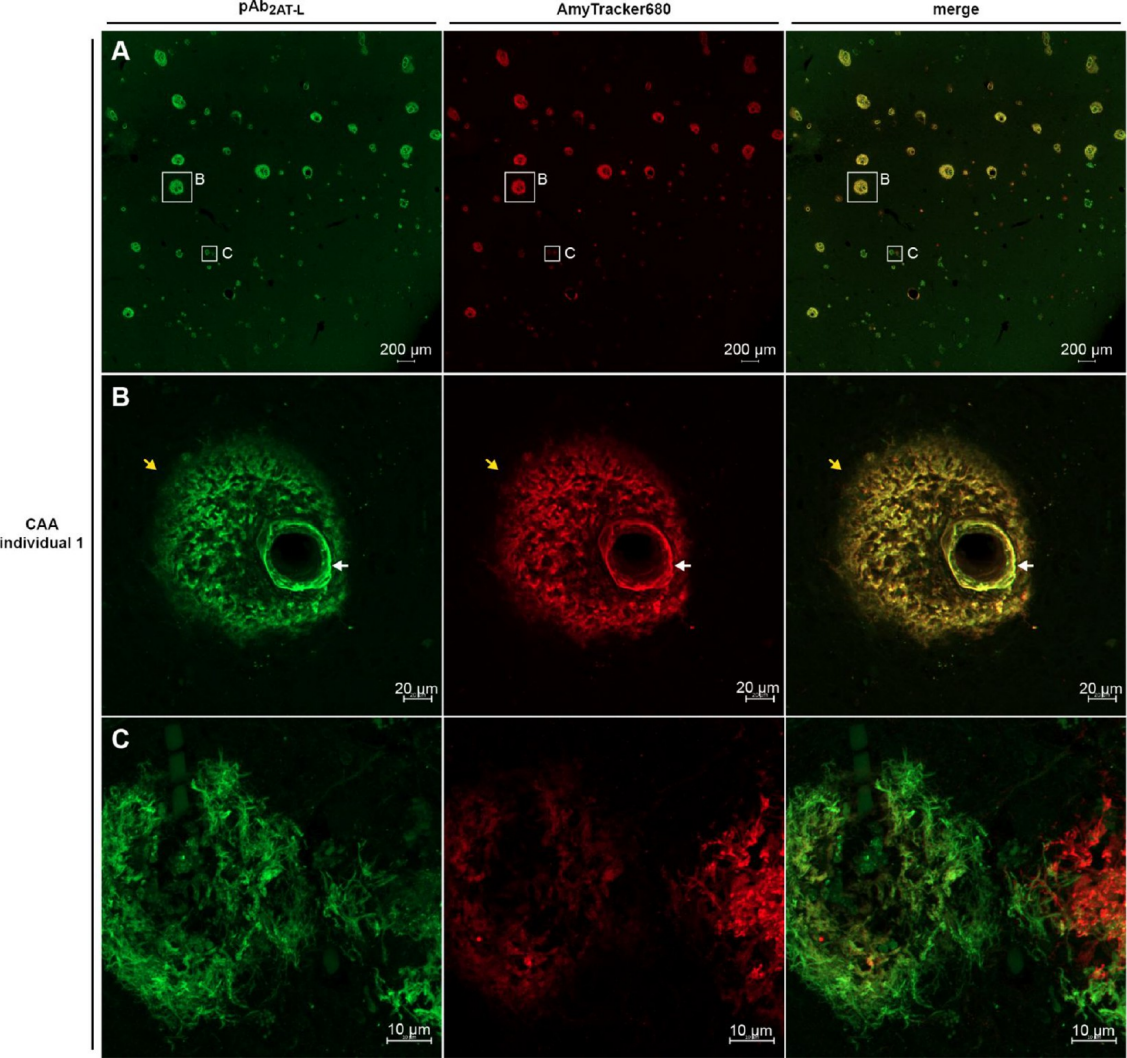
Confocal fluorescence micrographs of LOAD occipital cortex brain tissue containing CAA stained with pAb_2AT-L_ (green) and AmyTracker680 (red). (A) Representative stitched image (10× objective) of CAA and plaques in an occipital cortex brain slice. (B) Representative image (20× objective) of an arteriole in which pAb_2AT-L_ and AmyTracker680 have stained Aβ deposits in the arterial walls (white arrow) and around the arteriole in the perivascular neuropil (yellow arrow). (C) Representative image (63× objective) of plaques in the LOAD brain tissue containing CAA.

To investigate the immunoreactivity of pAb_2AT-L_ with Aβ deposits in CAA, we stained a brain slice from a LOAD individual exhibiting CAA pathology with pAb_2AT-L_ and AmyTracker680. Confocal fluorescence microscopy of the CAA brain slice revealed that pAb_2AT-L_ and AmyTracker680 strongly stain CAA pathology (Figure 5A). Figure 5B shows a representative image of an arteriole in which pAb_2AT-L_ and AmyTracker680 have stained Aβ deposits in the arterial walls (white arrow) and around the arteriole in the perivascular neuropil (yellow arrow). This staining and deposition pattern of Aβ is consistent with previous immunohistochemical studies of CAA brain tissue.^105−108^ The CAA tissue also contained Aβ plaques that exhibited pAb_2AT-L_ staining and AmyTracker680 staining similar to that observed in the DSAD brain slice (Figure 5C).

The CAA and Aβ plaque staining images show differences in the overlap of pAb_2AT-L_ and AmyTracker680. In the DSAD brain slices, pAb_2AT-L_ and AmyTracker680 exhibited little or no overlap in staining (Figure 4D–F). In contrast, pAb_2AT-L_ and AmyTracker680 exhibited significant overlap in staining in CAA (Figure 5). This variation is consistent with previous studies that have found that the Aβ deposits in CAA are distinct from Aβ in plaques. The 40-amino-acid alloform of Aβ (Aβ_40_) predominates in CAA^123^ and is thought to form fibrils composed of parallel and antiparallel β-sheets in CAA,^124^ while the 42-amino-acid alloform of Aβ (Aβ_42_) predominates in plaques and forms fibrils composed of only parallel β-sheets.^53,125^

The staining experiments with pAb_2AT-L_ in brain slices from individuals with Alzheimer’s disease indicate that the biogenic Aβ assemblies in these Alzheimer’s disease brains present epitopes that are similar to epitopes displayed on the synthetic Aβ oligomer mimic 2AT-L. These studies further support the biological significance of 2AT-L and suggest that these biogenic Aβ assemblies may resemble 2AT-L. The pAb_2AT-L_ staining experiments in LOAD individuals, DSAD individuals, and a LOAD individual with CAA provide a broad overview of the immunostaining properties of pAb_2AT-L_ with Alzheimer’s disease brain tissue and indicate that antibodies raised against 2AT-L strongly bind pathological Aβ assemblies formed in these Alzheimer’s disease brains.

### Immunoreactivity of pAb_2AT-L_ with Brain Tissue from 5xFAD mice

Alzheimer’s disease transgenic mouse models have aided in understanding Aβ plaque formation and its relationship to the pathogenesis and progression of Alzheimer’s disease. The Alzheimer’s disease mouse model 5xFAD contains five mutations associated with early onset Alzheimer’s disease that lead to overproduction of Aβ_42_.^126^ 5xFAD mice exhibit accelerated Aβ plaque deposition that begins at 2 months and progresses rapidly, reaching a large plaque burden by 4–6 months and continuing to progress as the mouse ages. To explore the relationship between the trimer 2AT-L and biogenic Aβ assemblies formed in 5xFAD mouse brains, we performed immunohistochemical and immunoblotting experiments with pAb_2AT-L_ on brain tissue from 5xFAD mice.

**Figure 6:**
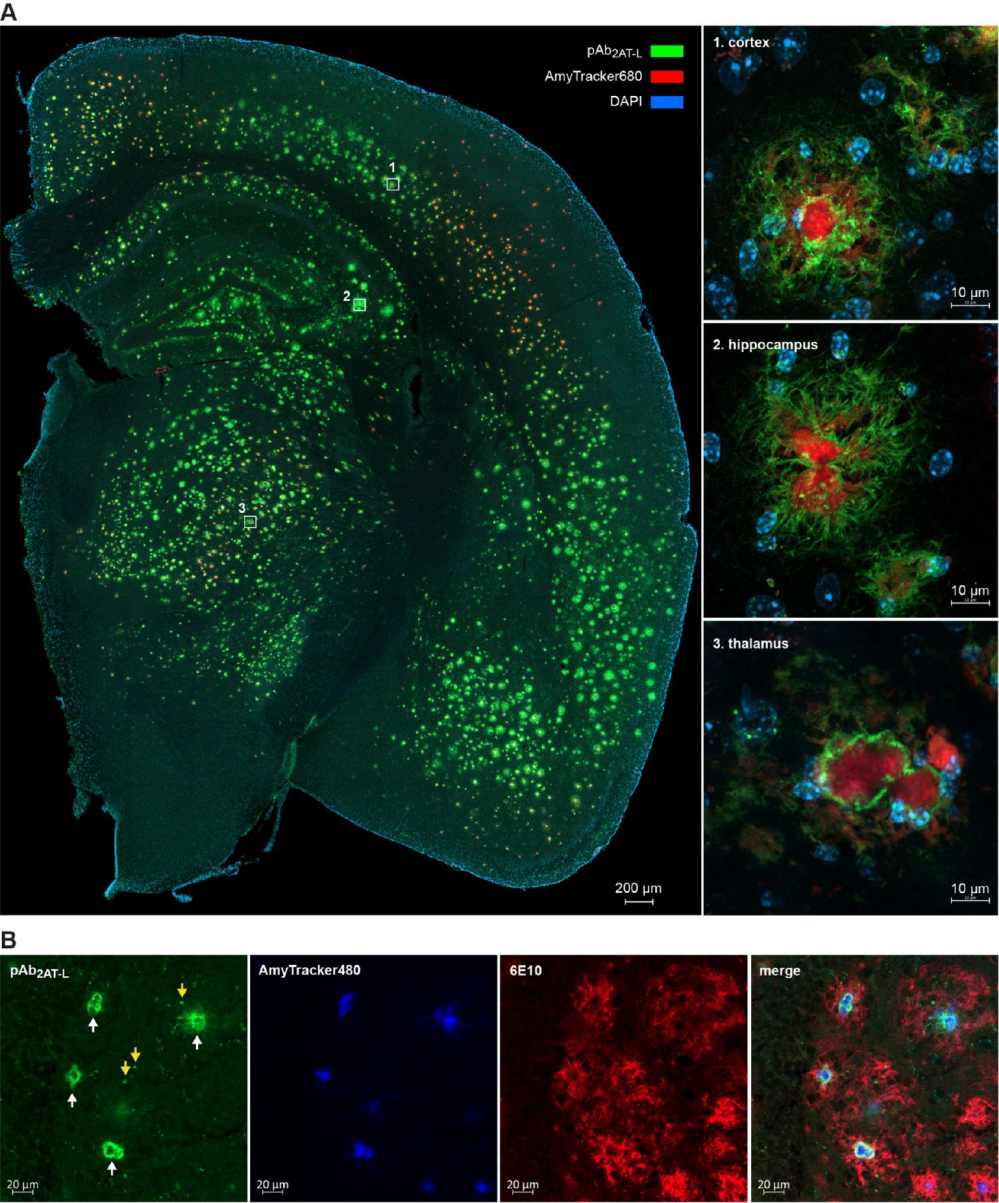
Confocal fluorescence micrographs of 5xFAD brain tissue. (A, left) Representative stitched image (10× objective) of a coronal brain section from a 13-month-old female 5xFAD mouse stained with pAb_2AT-L_ (green), AmyTracker680 (red), and DAPI (blue). (A, right) Representative images (63× objective) of plaques in the (1) isocortex, (2) CA3 region of hippocampus, and (3) thalamus of the 5xFAD brain slice. (B) Representative images (20× objective) of plaques in the cortex of a 13-month-old female 5xFAD mouse after extended washing after immunostaining with pAb_2AT-L_ (green) and 6E10 (red) and then subsequently staining with AmyTracker480 (blue). White arrows in the first panel designate the staining of the direct periphery of the cores by pAb_2AT-L_; yellow arrows in the first panel designate the punctate features stained by pAb_2AT-L_.

We investigated the immunoreactivity of pAb_2AT-L_ with Aβ assemblies in 5xFAD mouse brains by staining brain slices from a 13-month-old 5xFAD mouse and a 13-month-old wild type control mouse with pAb_2AT-L_ and the amyloid-binding dye AmyTracker680 (Ebba Biotech).^127,128^ Confocal fluorescence microscopy of the 5xFAD mouse brain slice reveals that pAb_2AT-L_ binds to the outer, more diffuse Aβ deposits of the plaques (Figure 6A). Higher magnification images of representative plaques in the cortex, hippocampus, and thalamus show that the peripheries around the dense cores of the plaques exhibit the most intense staining by pAb_2AT-L_, and that the diffuse Aβ exhibits weaker, albeit still significant staining (boxed insets in Figure 6A). No staining of the dense cores by pAb_2AT-L_ was observed, and no significant staining of the diffuse Aβ around the dense cores by AmyTracker680 was observed; thus there is little or no overlap in staining between pAb_2AT-L_ and AmyTracker680. No significant staining was observed in the wild type control (Figure S12). DAB staining with pAb_2AT-L_ of a brain slice from an 8-month-old 5xFAD mouse further illustrates the Aβ plaque staining by pAb_2AT-L_ (Figure S13).

To further assess the immunostaining properties of pAb_2AT-L_ in 5xFAD mouse brain slices, we performed a subsequent experiment in which we triple labeled the plaques with pAb_2AT-L_, 6E10, and AmyTracker480 and extended the washing step after immunostaining. In the staining experiment described in the preceding paragraph and detailed in Figure 6A, we washed the tissue three times for 5 min in TBS with 0.1% Triton X-100 (TBSX) after immunostaining. In the subsequent triple-labeling experiment, we washed the tissue two times in TBSX for 5 min and then overnight (∼16 h) in TBSX after immunostaining. Confocal fluorescence microscopy of this brain slice revealed that extended washing eliminated the weaker pAb_2AT-L_ staining of the diffuse Aβ around the dense cores but left the pAb_2AT-L_ staining of the direct peripheries of the dense cores (white arrows in Figure 6B). The extended washing also accentuated punctate features stained by pAb_2AT-L_ that appear to reside within the diffuse Aβ around the cores (yellow arrows in Figure 6B). In contrast, the 6E10 staining of the peripheries of the dense cores and the diffuse Aβ around the dense cores is still prominent after the extended washing.

The staining experiments with pAb_2AT-L_ in 5xFAD mouse brain slices indicate that biogenic Aβ assemblies produced in 5xFAD mice present epitopes that are similar to epitopes displayed on 2AT-L, positively correlating 2AT-L with biogenic Aβ and further establishing 2AT-L as a suitable model for an Aβ oligomer. The immunostaining observed after extended washing suggests that among the antibodies in the pAb_2AT-L_ polyclonal antibody mixture, the strongest binders recognize unique features of the plaques in 5xFAD mice—the peripheries of the dense cores and punctate features embedded in the diffuse Aβ around the cores. The staining of these unique features by pAb_2AT-L_ suggests that these features are structurally distinct from the dense cores and the outer diffuse Aβ around the cores, and that pAb_2AT-L_ predominantly recognizes Aβ epitopes that are conformationally distinct from the Aβ epitopes of the diffuse Aβ around the dense cores. To our knowledge, antibodies that specifically stain the direct peripheries of the dense cores of plaques have not been previously reported.

Previous studies have shown that Aβ plaques contain structurally distinct Aβ assemblies, including both fibrils and oligomers.^61,129^ Ashe and co-workers isolated the dense Aβ cores and the diffuse Aβ around the cores from rTg9191 mouse brains using laser microdissection.^129^ Immunological analyses of these different plaque regions revealed that the putative Aβ dodecamer Aβ*56 and other Aβ oligomers are almost exclusively found in the diffuse Aβ of the plaques, although recent reports have called the identification, characterization, and study of Aβ*56 into question.^130,131^ Walsh and co-workers dissolved Aβ plaques from Alzheimer’s disease individuals and used LC-MS/MS to show that the plaques contain heterogeneously cross-linked dimers of different Aβ alloforms.^38^ While we do not know the three-dimensional structures of the Aβ assemblies recognized by pAb_2AT-L_ in the brain or the relationship between the dodecamer formed by 2AT-L and the putative Aβ*56 dodecamer, the staining of unique features in plaques by pAb_2AT-L_ is consistent with the model that Aβ plaques are composed of structurally diverse Aβ assemblies.

### Immunoreactivity of pAb_2AT-L_ against 5xFAD Brain Protein Extract

To corroborate that pAb_2AT-L_ recognizes biogenic Aβ in tissue, we performed biochemical experiments on brain protein extracts from 5xFAD mouse brains. We first performed a dot blot experiment to determine extraction conditions for isolating pAb_2AT-L_-reactive species. We then performed immunoprecipitation mass spectrometry experiments in which we analyzed the species pulled down by pAb_2AT-L_ by LC-MS.

**Figure 7:**
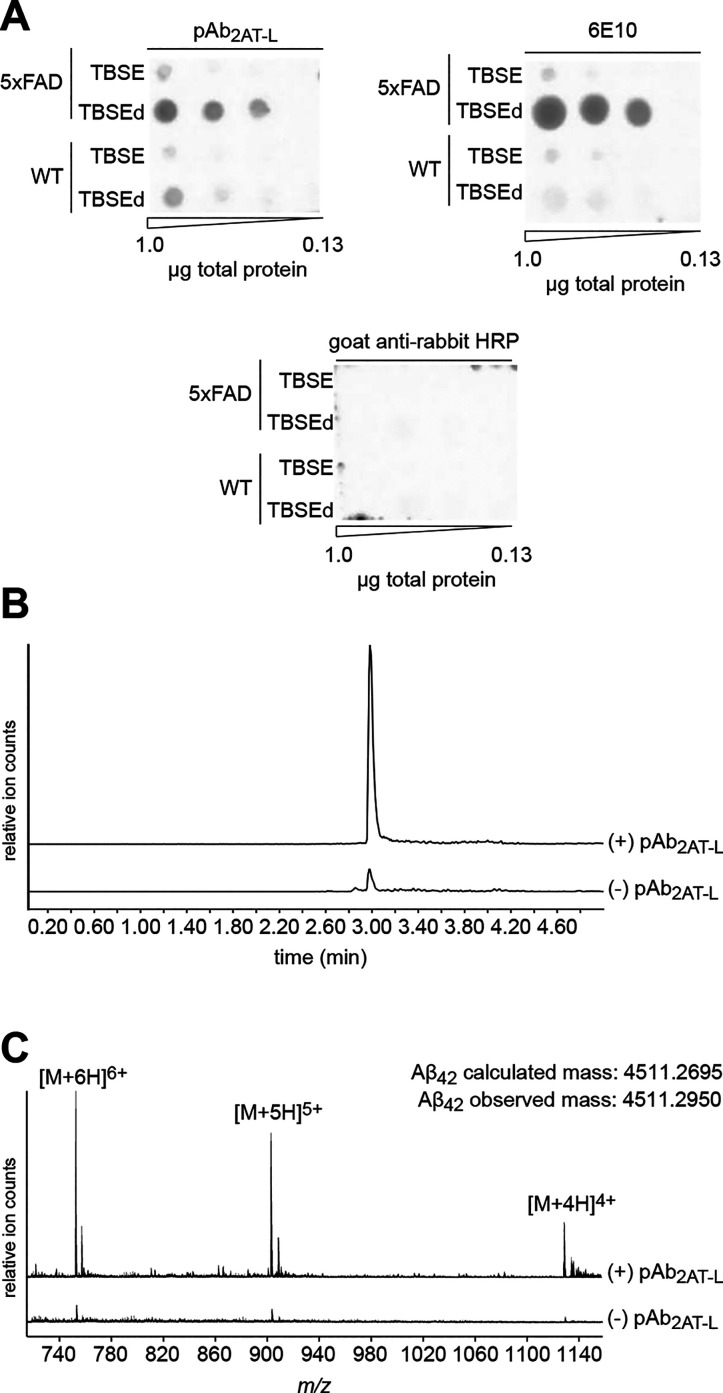
Biochemical analysis of pAb_2AT-L_ immunoreactivity with 5xFAD brain protein extracts. (A) Dot blot analysis of the immunoreactivity of pAb_2AT-L_, 6E10, and goat antirabbit HRP with protein extracts from 5xFAD and WT mouse brains. (B) LC-MS chromatograms of Aβ_42_ pulled down from TBSEd 5xFAD mouse brain protein extract with protein A/G Dynabeads in the presence (+) or absence (−) of pAb_2AT-L_. Chromatograms are filtered for the *m*/*z* of Aβ_42_. (C) Mass spectra of the Aβ_42_ peaks from B. The top spectrum corresponds to the top peak in B in which pAb_2AT-L_ was present in the pull-down experiment and shows the three Aβ_42_ charge states observed. The bottom spectrum corresponds to the bottom peak in B in which pAb_2AT-L_ was absent.

To determine extraction conditions for isolating pAb_2AT-L_-reactive species, we adapted a protein extraction protocol first described by Ashe and co-workers^41^ and then performed dot blot analysis on the protein extracts. In this extraction protocol, we fractionated brain proteins from a 5xFAD mouse and WT mouse into proteins soluble in TBSE (50 mM Tris buffer at pH 7.4, 100 mM NaCl, 1 mM EDTA) and proteins soluble in TBSE with detergents (TBSEd; TBSE with 3% SDS, 0.5% Triton-X, and 0.1% deoxycholate). We then spotted equal quantities of these protein extracts on nitrocellulose membranes and performed standard immunoblotting procedures with pAb_2AT-L_, 6E10, and the negative control antibody goat antirabbit-IgG-HRP. The dot blots show that pAb_2AT-L_ predominantly recognizes protein in the 5xFAD TBSEd fraction, showing weaker recognition of protein in the WT TBSEd extract and little or no recognition of protein in the TBSE extracts from both 5xFAD and WT brains (Figure 7A). 6E10 exhibits similar recognition properties to those of pAb_2AT-L_, showing weaker recognition toward protein in the WT TBSEd extract than pAb_2AT-L_. The goat antirabbit-IgG-HRP antibody exhibits no reactivity with proteins in any of the extracts.

To determine the molecular identity of the protein species that pAb_2AT-L_ recognizes in the 5xFAD TBSEd fraction, we turned to immunoprecipitation liquid chromatography mass spectrometry (IP-LC-MS). In these experiments, we immunoprecipitated from the 5xFAD TBSEd fraction with protein A/G Dynabeads in the presence (+) or absence (−) of pAb_2AT-L_. We then washed the Dynabeads and decomplexed the bound material by treating the Dynabeads with 88% formic acid.^132,133^ Comparison of the (+) pAb_2AT-L_ and (−) pAb_2AT-L_ LC-MS chromatograms shows a peak at 2.98 min that is ∼10-fold more prominent in the (+) pAb_2AT-L_ sample than the (−) pAb_2AT-L_ sample (Figures 7B and S14). Mass spectrometric analysis reveals that this peak is Aβ_42_ (Figures 7C and S14). These results indicate that during the immunoprecipitation, pAb_2AT-L_ engages with and binds biogenic Aβ_42_ in a mixture of 5xFAD brain proteins. These results also suggest that the molecular identity of the species that pAb_2AT-L_ recognizes in the tissue staining experiments is Aβ and not another protein associated with the plaques.

## Summary and Conclusion

The structures of Aβ oligomers that form during Alzheimer’s disease pathogenesis and progression are unknown, constituting a significant gap in understanding the disease. Elucidating the structures of disease-relevant Aβ assemblies that form in the brain enhances our understanding of Alzheimer’s disease and holds the promise of developing better drugs that prevent or alter the course of the disease. The approach described in this paper provides a roadmap for filling this gap in understanding. This approach includes: (1) designing and synthesizing conformationally constrained Aβ β-hairpin peptides, (2) elucidating the structures of the oligomers that the Aβ β-hairpin peptides form using X-ray crystallography, (3) designing and synthesizing covalently stabilized Aβ oligomer models, (4) studying the structural, biophysical, and biological properties of the Aβ oligomer models, (5) generating antibodies against the Aβ oligomer models, and (6) characterizing the immunoreactivity of the antibodies with transgenic mouse and human brain tissue.

In this paper, we use the approach above to study the Aβ oligomer model 2AT-L, a covalently stabilized triangular trimer composed of Aβ_17–36_ β-hairpin peptides. These studies support the biological significance of 2AT-L as an Aβ oligomer model and suggest that Aβ assemblies that form in the brain may share structural features with 2AT-L. Structural, biophysical, and cell-based studies indicate that 2AT-L shares characteristics with oligomers formed by full-length Aβ. X-ray crystallography reveals the high-resolution structure of 2AT-L and shows that four copies of 2AT-L further assemble to form a ball-shaped dodecamer. SDS-PAGE demonstrates that 2AT-L also assembles to form a dodecamer in membrane-like environments, and cell-based studies revealed that 2AT-L is toxic toward cells. Studies with the antibody pAb_2AT-L_ indicate that 2AT-L promotes the generation of antibodies that recognize Aβ *in vitro*, with some selectivity for aggregated forms of Aβ, as well as Aβ in unique pathological features in brain tissue. Immunostaining brain slices from LOAD and DSAD individuals demonstrates that pAb_2AT-L_ recognizes different types of Aβ plaques in Alzheimer’s disease brains. Immunostaining a brain slice from a LOAD individual with CAA shows that pAb_2AT-L_ recognizes Aβ that deposits around blood vessels in the brains. Immunostaining of a brain slice from a 5xFAD mouse reveals that pAb_2AT-L_ recognizes Aβ around the direct peripheries of the dense cores of Aβ plaques, and immunoprecipitation LC-MS studies demonstrate that pAb_2AT-L_ engages and binds Aβ in a mixture of brain proteins and corroborates that pAb_2AT-L_ is recognizing Aβ in the immunostaining studies.

The immunoreactivity of pAb_2AT-L_ with Aβ assemblies present in plaques and CAA demonstrates that antibodies raised against 2AT-L recognize the Aβ assemblies present in these pathologies and suggests that these assemblies may share structural similarities with 2AT-L. These findings represent an important step toward understanding the structures of Aβ assemblies that form in the brain. Furthermore, these findings set the stage for pursing monoclonal antibodies against 2AT-L as well as other Aβ oligomer models our laboratory has developed.

## Data Availability

Crystallographic coordinates of 2AT-L were deposited into the Protein Data Bank (PDB) with code 7U4P.

## Supporting Information Available

Supporting figures, X-ray crystallography data collection and refinement statistics, materials and methods, characterization data (PDF)
