# Supplementary material for: Antibodies Raised Against an Aβ Oligomer Mimic Recognize Pathological Features in Alzheimer’s Disease and Associated Amyloid-Disease Brain Tissue
Source: ACS Cent Sci. 2023 Dec 21;10(1):104–21. doi: 10.1021/acscentsci.3c00592 (PMC10823522; doi:10.1021/acscentsci.3c00592)
Supplement: Supplementary file 1 — oc3c00592_si_001.pdf [file oc3c00592_si_001.pdf]

Supporting Information for:

Antibodies Raised Against an Aβ Oligomer Mimic Recognize Pathological Features in Alzheimer's Disease and Associated Amyloid-Disease Brain Tissue

Adam G. Kreutzer,[a]* Chelsea Marie T. Parrocha,[b] Sepehr Haerianardakani,[a] Gretchen Guaglianone,[a] Jennifer T. Nguyen,[b] Michelle N. Diab,[a] William Yong,[c] Mari Perez-Rosendahl,[c] Elizabeth Head,[c] and James S. Nowick[a,b]*

[a]Department of Chemistry, University of California Irvine, Irvine, California, 92697, USA
[b]Department of Pharmaceutical Sciences, University of California Irvine, Irvine, California, 92697, USA.
[c]Department of Pathology and Laboratory Medicine University of California Irvine, Irvine, California, 92697, USA.

*Corresponding authors:
Adam G. Kreutzer, 4302 Natural Sciences 1, University of California Irvine, Irvine, CA 92697
James S. Nowick, 4126 Natural Sciences 1, University of California Irvine, Irvine, CA 92697

Email: akreutze@uci.edu and jsnowick@uci.edu

This PDF includes:

Supporting Figures and Table

Figure S1. X-ray crystallographic structures of triangular trimers composed of β-hairpin peptides derived from $A\beta_{17-36}$. S4

Figure S2. An annular pore-like structure formed by six 2AT-L dodecamers. S5

Figure S3. Images of silver-stained SDS-PAGE with no contrast adjustment and increased contrast. S6

Figure S4. Enlarged image of the SEC chromatograms from Figure 2G containing the size standard bovine serum albumin (BSA). S7

Figure S5. Fluorescence micrograph of a brain slice from LOAD individual 1 stained with pAb_{2AT-L}. S8

Figure S6. Fluorescence micrograph of a brain slice from LOAD individual 2 stained with pAb_{2AT-L}. S9

Figure S7. Fluorescence micrograph of a brain slice from DSAD individual 1 stained with pAb_{2AT-L}. S10

Figure S8. Fluorescence micrograph of a brain slice from DSAD individual 2 stained with pAb_{2AT-L}. S11

Figure S9. Fluorescence micrograph of a brain slice from DSAD individual 3 stained with pAb_{2AT-L}. S12

Figure S10. Fluorescence micrograph of a brain slice from DSAD individual 4 stained with pAb_{2AT-L}. S13

Figure S11. Micrograph of a frontal cortex brain slice from DSAD individual 1 DAB stained with $pAb_{2AT\text{-}L}$. S14

Figure S12. Representative stitched image of a coronal brain section from a 13-month-old female wild type mouse stained with $pAb_{2AT\text{-}L}$ and DAPI. S15

Figure S13. Micrograph of a coronal brain slice from an 8-month-old female 5xFAD mouse DAB stained with $pAb_{2AT\text{-}L}$. S16

Figure S14. IP-LC-MS data from immunoprecipitation with $pAb_{2AT\text{-}L}$ from 5xFAD mouse brain protein extract. S17

Table S1. Crystallographic properties, crystallization conditions, and data collection and model refinement statistics for 2AT-L. S18

Materials and Methods

General information. S19

Synthesis and purification of 2AM-L and 2AM-L_{CC}. S19

Loading of the resin. S19

Peptide coupling. S19

Cleavage of the peptide from the resin. S20

Cyclization of the linear peptide. S20

Global deprotection of the cyclic peptide. S20

Reverse-phase HPLC purification. S21

Scheme 1. Synthetic scheme for 2AM-L. S22

Synthesis, purification, and characterization of 2AT-L. S23

Synthesis of 2AT-L. S23

LC-MS analysis of the 2AM-L_{CC} oxidation reaction mixture. S23

Purification of 2AT-L. S24

LC-MS characterization of 2AT-L. S25

X-ray crystallography of 2AT-L. S26

Crystallization procedure for 2AT-L. S26

X-ray crystallographic data collection, data processing, and structure determination. S26

SDS-PAGE and silver staining. S27

Sample preparation and gel running. S27

Silver staining. S27

Size exclusion chromatography (SEC). S28

Dynamic light scattering (DLS). S29

Circular dichroism (CD) spectroscopy. S29

Cell-based toxicity assays of 2AT-L and 2AM-L in SH-SY5Y cells. S29

Preparation of SH-SY5Y cells for the toxicity assays. S30
Preparation of 2AT-L and 2AM-L for toxicity assays. S30
Treatment of the SH-SY5Y cells with 2AT-L and 2AM-L. S31
CyQUANT™ LDH Cytotoxicity Assay. S31
CellTiter-Glo® 2.0 Cell Viability Assay. S31
Apo-ONE® Homogeneous Caspase-3/7 Assay. S32
Generation of $pAb_{2AT\text{-}L}$. S32
Rabbit immunization. S32
Affinity purification of $pAb_{2AT\text{-}L}$. S33
Preparation of $A\beta_{42}$. S35
Indirect ELISAs. S35
Coating the wells of the ELISA plates. S35
Treating the ELISA plates with $pAb_{2AT\text{-}L}$ or 6E10. S36
Treating the ELISA plate with the secondary antibodies. S37
Developing the ELISA plates. S37
Western blots. S38
Time course $A\beta_{42}$ aggregation dot blots. S39
ThT assay on $A\beta_{42}$. S41
Immunostaining and microscopy of human and 5xFAD mouse brain slices. S42
Preparing the human brain tissues for immunostaining. S42
Preparing the 5xFAD mouse brain tissues for immunostaining. S42
Immunofluorescence staining and imaging of the human and 5xFAD mouse brain tissue slices. S43
DAB staining and imaging of the human and 5xFAD mouse brain tissue slices. S45
Preparation of 5xFAD brain protein extracts. S46
Dot blot assay of $pAb_{2AT\text{-}L}$ against 5xFAD brain protein extracts. S47
Immunoprecipitation LC-MS on 5xFAD brain protein extracts. S48
References and Notes S50
Characterization Data
Characterization of 2AM-L. S51
Characterization of 2AT-L. S53

A

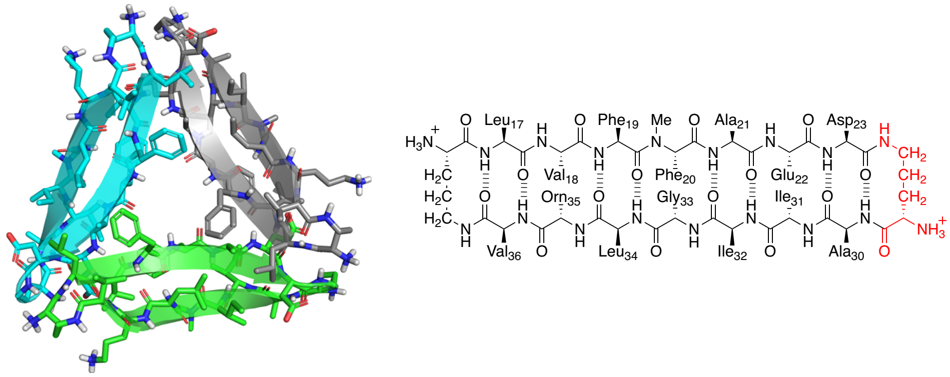

trimer from Aβ$_{17-36}$
PDB 4NW9
JACS, **2014**, *136*, 5595–5598

B

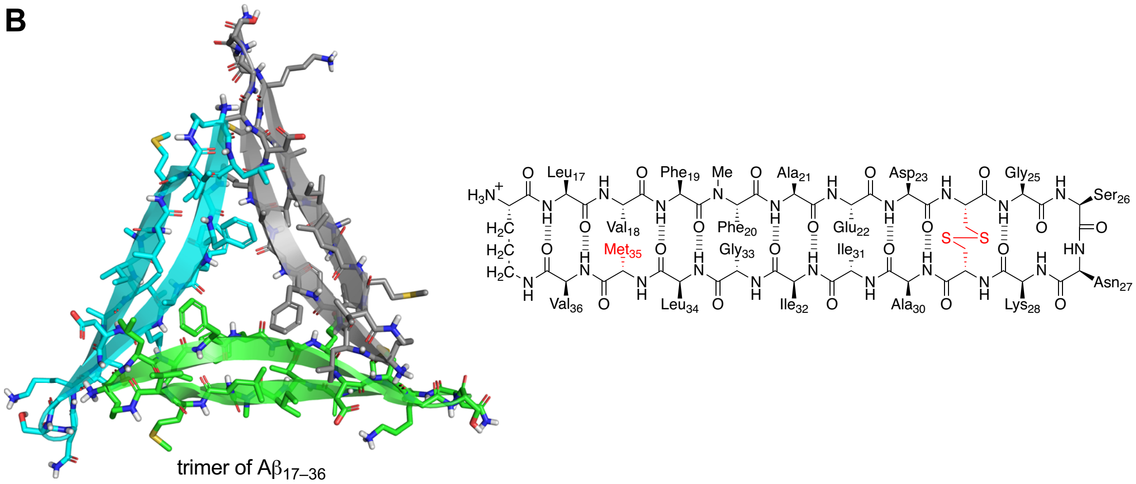

trimer of Aβ$_{17-36}$
PDB 5HOX
JACS, **2016**, *138*, 4634–4642

C

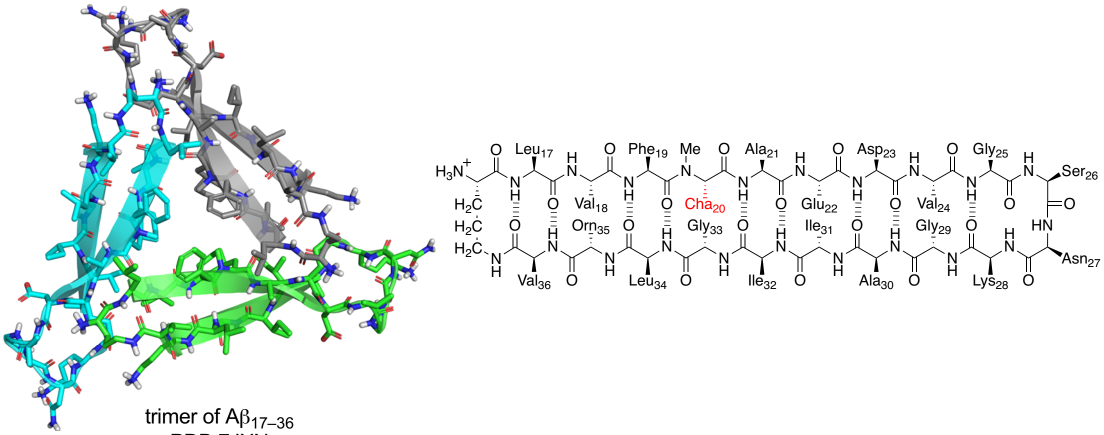

trimer of Aβ$_{17-36}$
PDB 7JXN
JACS, **2020**, *142*, 20708–20716

Figure S1. X-ray crystallographic structures of triangular trimers composed of β-hairpin peptides derived from Aβ$_{17-36}$. The amino acids in red in the chemical structures of the peptides designate the differences between these peptides and 2AM-L and the monomer components of 2AT-L. ***(A)*** Trimer composed of peptides derived from an Aβ$_{17-36}$ β-hairpin that lacks the Aβ$_{24-29}$ loop. ***(B)*** Trimer composed of Aβ$_{17-36}$ β-hairpins that contain an intramolecular disulfide linkage between positions 24 and 29 to reinforce β-hairpin conformation. ***(C)*** Trimer composed of Aβ$_{17-36}$ β-hairpins that contain cyclohexylalanine at position 20 in place of phenylalanine.

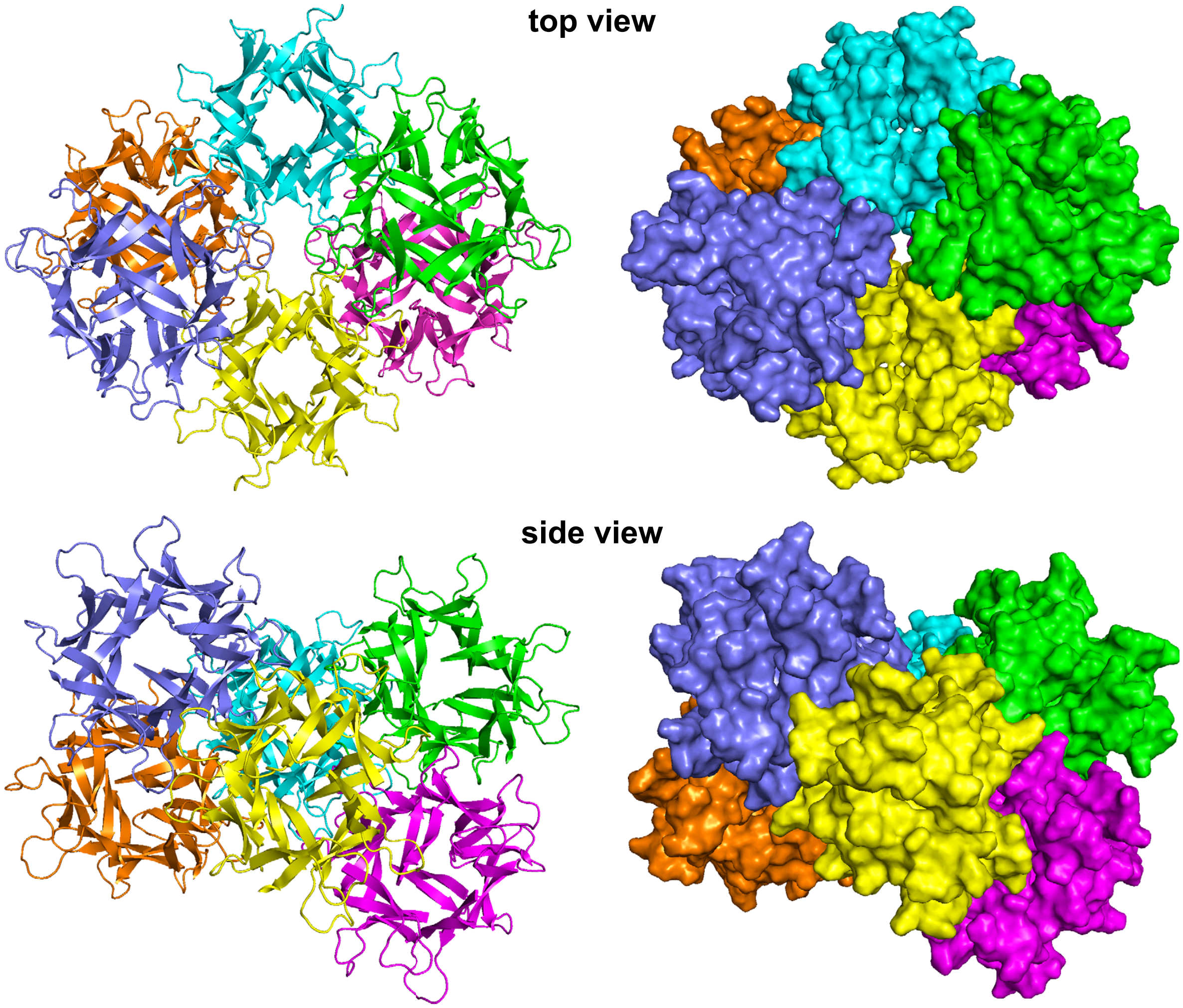

Figure S2. Cartoon and surface models of an annular pore-like structure formed by six 2AT-L dodecamers. Each dodecamer is shown as a different color.

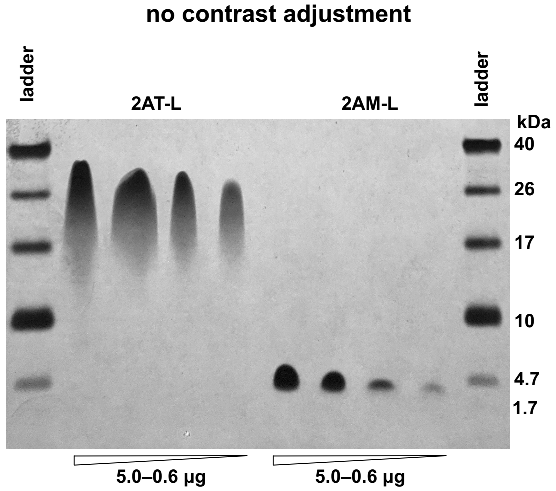

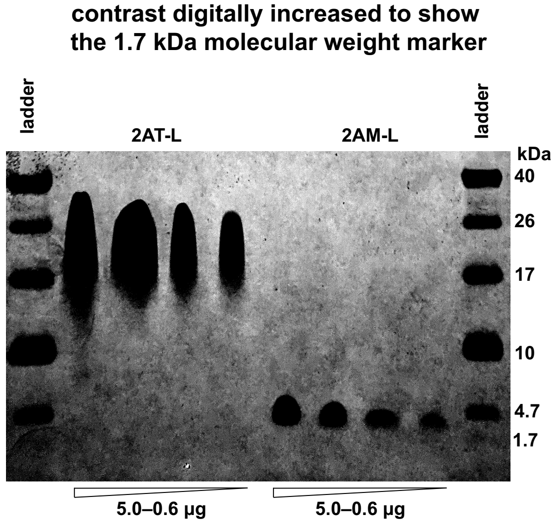

Figure S3. Two images of the silver-stained SDS-PAGE gel from Figure 2F. In the top image the contrast has not been adjusted; in the bottom image the contrast has been digitally increased so that the 1.7-kDa molecular weight marker is visible. The 2AM-L bands run at or below the 4.7-kDa molecular weight marker and above the 1.7-kDa molecular weight marker.

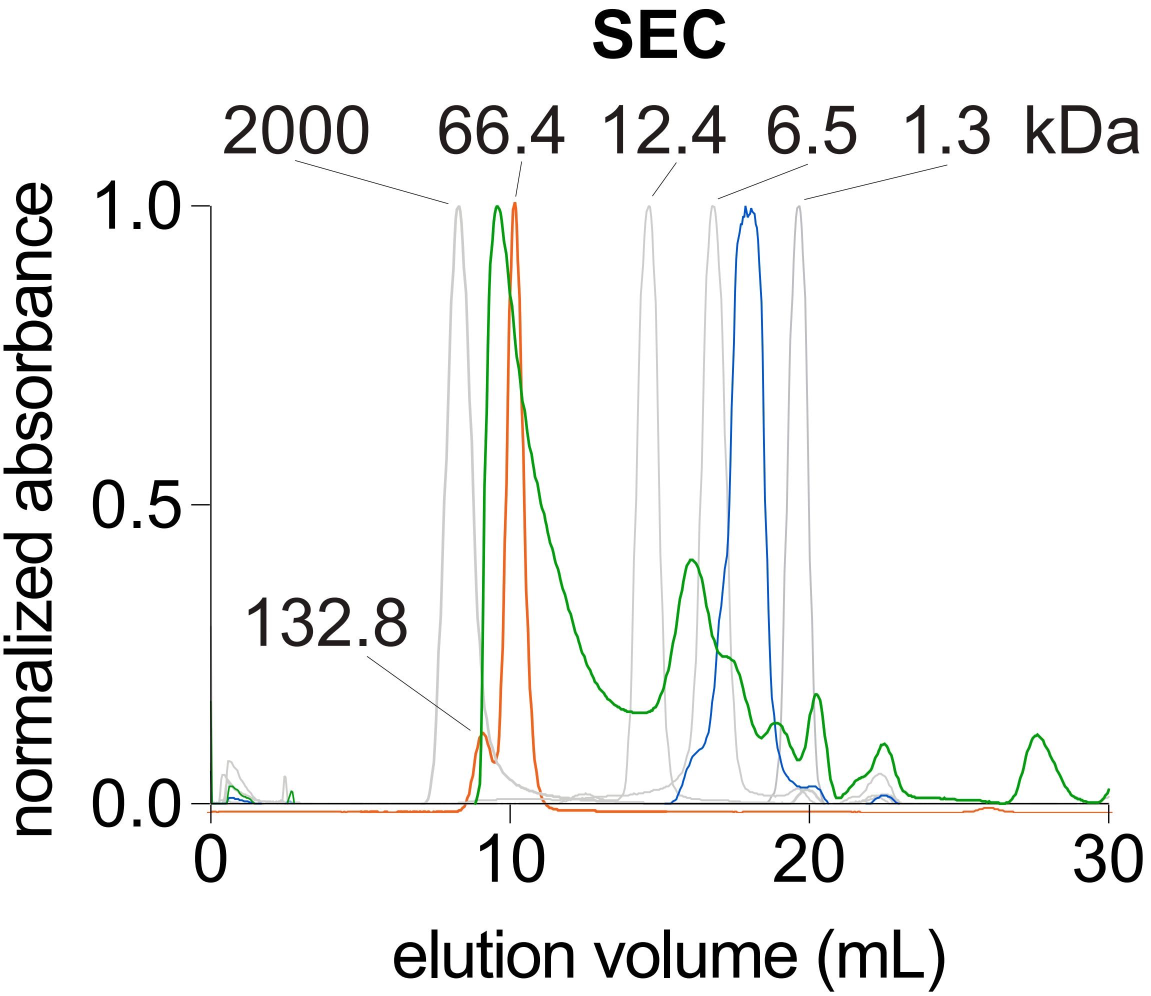

Figure S4. Enlarged image of the SEC chromatograms from Figure 2G containing the size standard bovine serum albumin (BSA), which elutes as two peaks. The BSA chromatogram is colored orange to highlight both BSA peaks. The BSA peak that elutes at 9.1 mL corresponds to a BSA dimer (132.8 kDa); the BSA peak that elutes at 10.2 mL corresponds to a BSA monomer (66.4 kDa). The other size standards correspond to dextran blue (2000 kDa), cytochrome C (12.4 kDa), aprotinin (6.5 kDa), and vitamin B_{12} (1.3 kDa). The chromatogram for 2AT-L is colored green; the chromatogram for 2AM-L is colored blue.

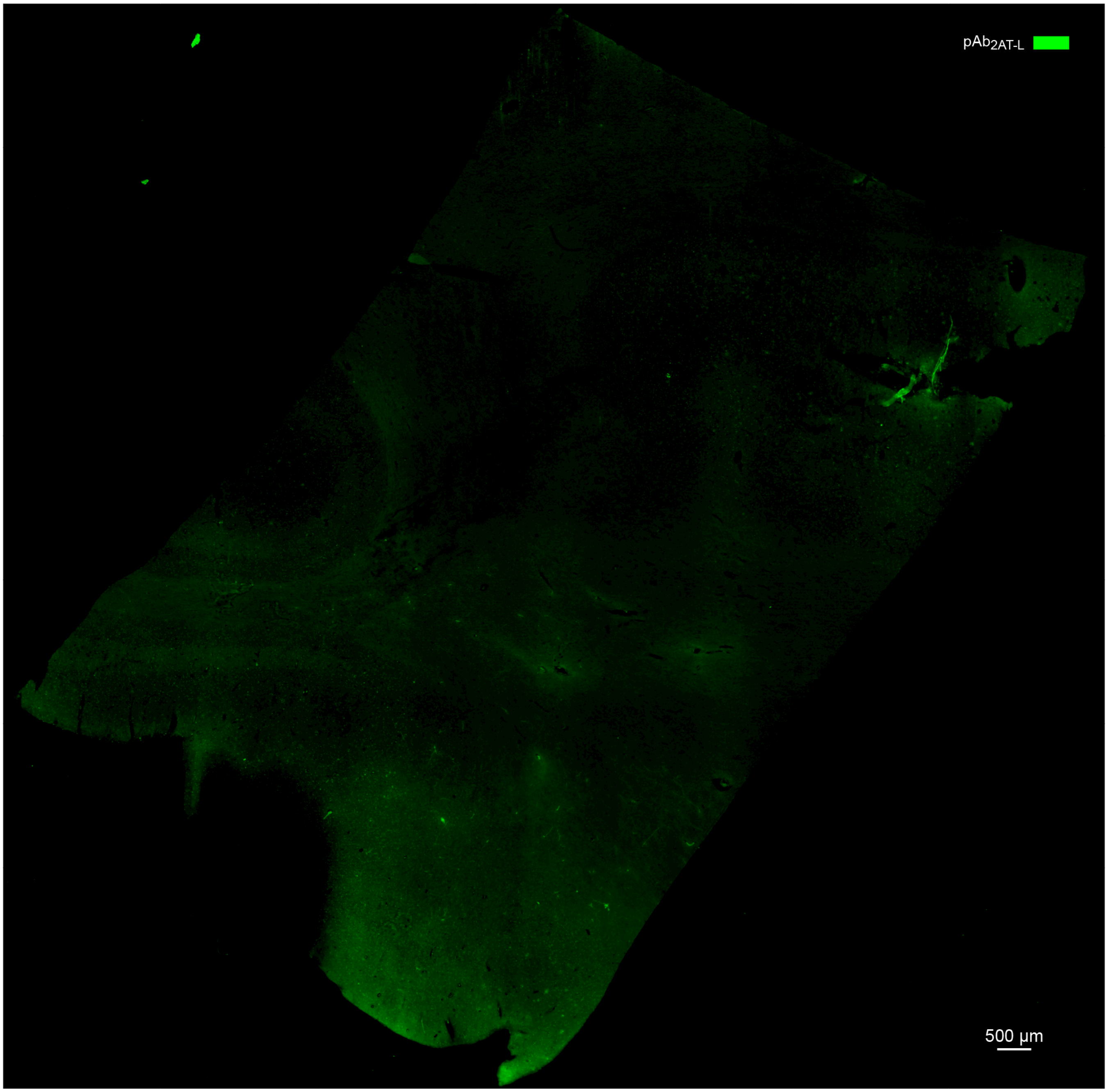

Figure S5. Fluorescence micrograph (4x objective, stitched image) of a frontal cortex brain slice from LOAD individual 1 stained with $pAb_{2AT\text{-}L}$ (green).

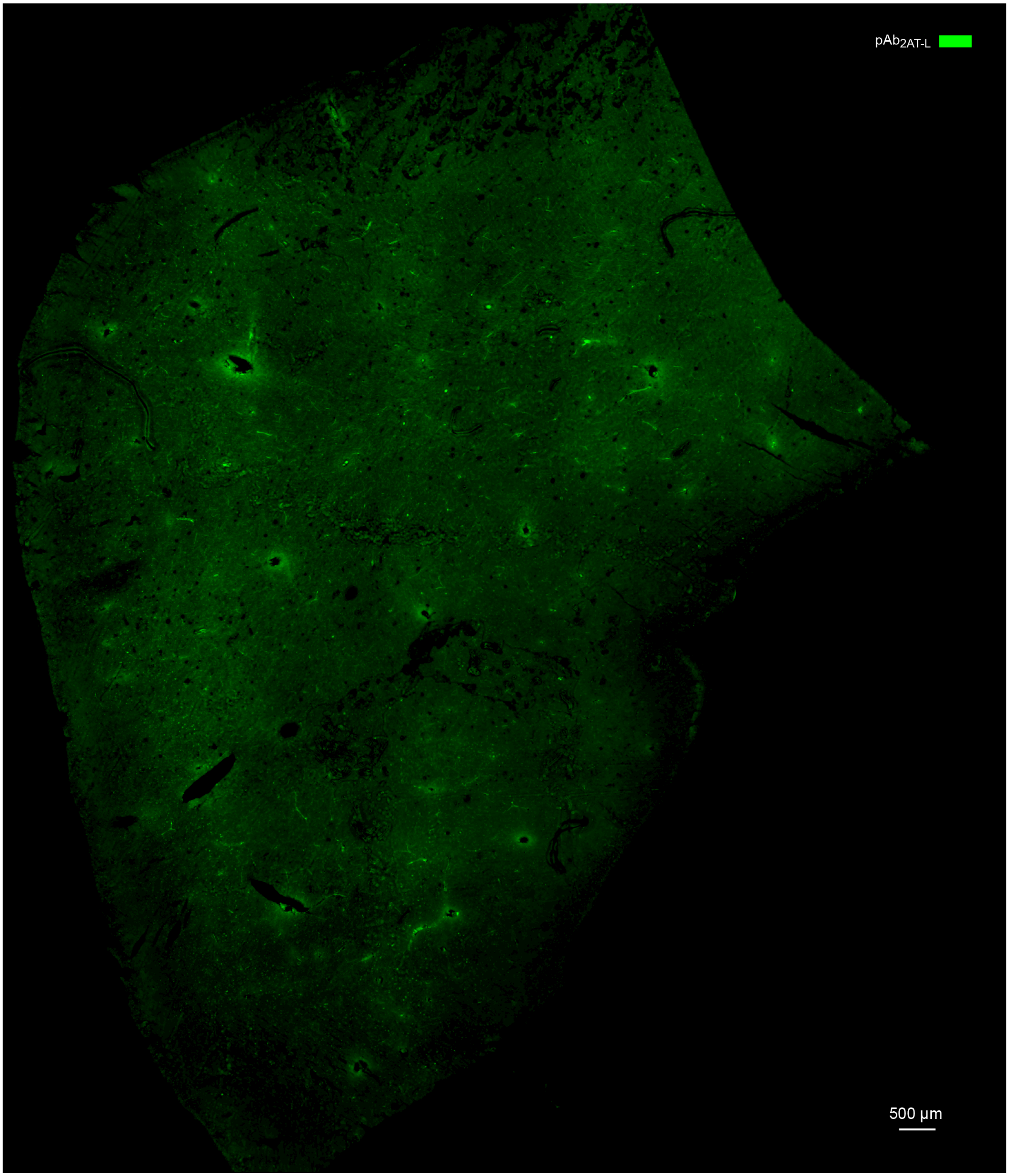

Figure S6. Fluorescence micrograph (4x objective, stitched image) of a frontal cortex brain slice from LOAD individual 2 stained with pAb_{2AT-L} (green).

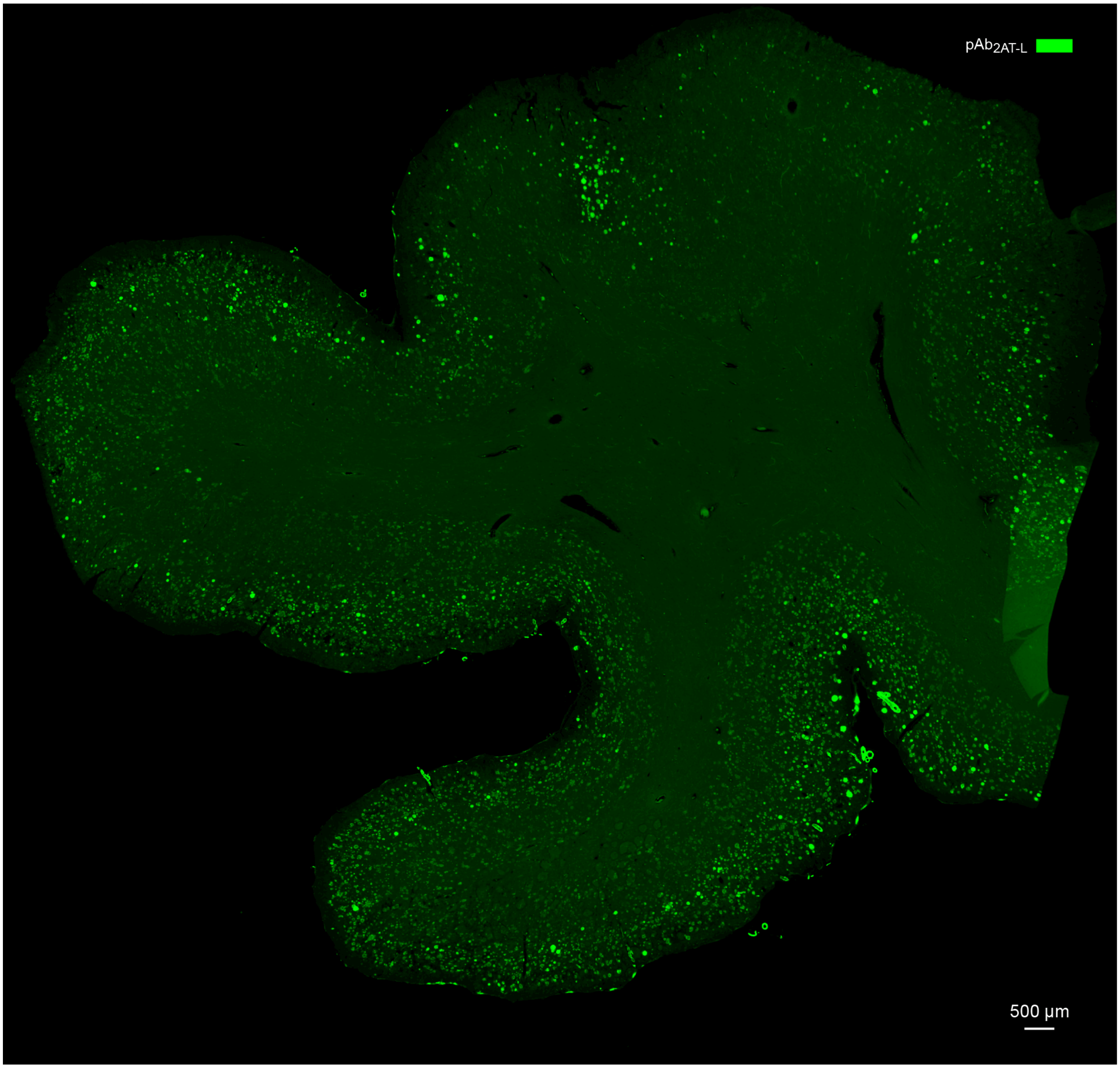

Figure S7. Fluorescence micrograph (4x objective, stitched image) of a frontal cortex brain slice from DSAD individual 1 stained with pAb_{2AT-L} (green).

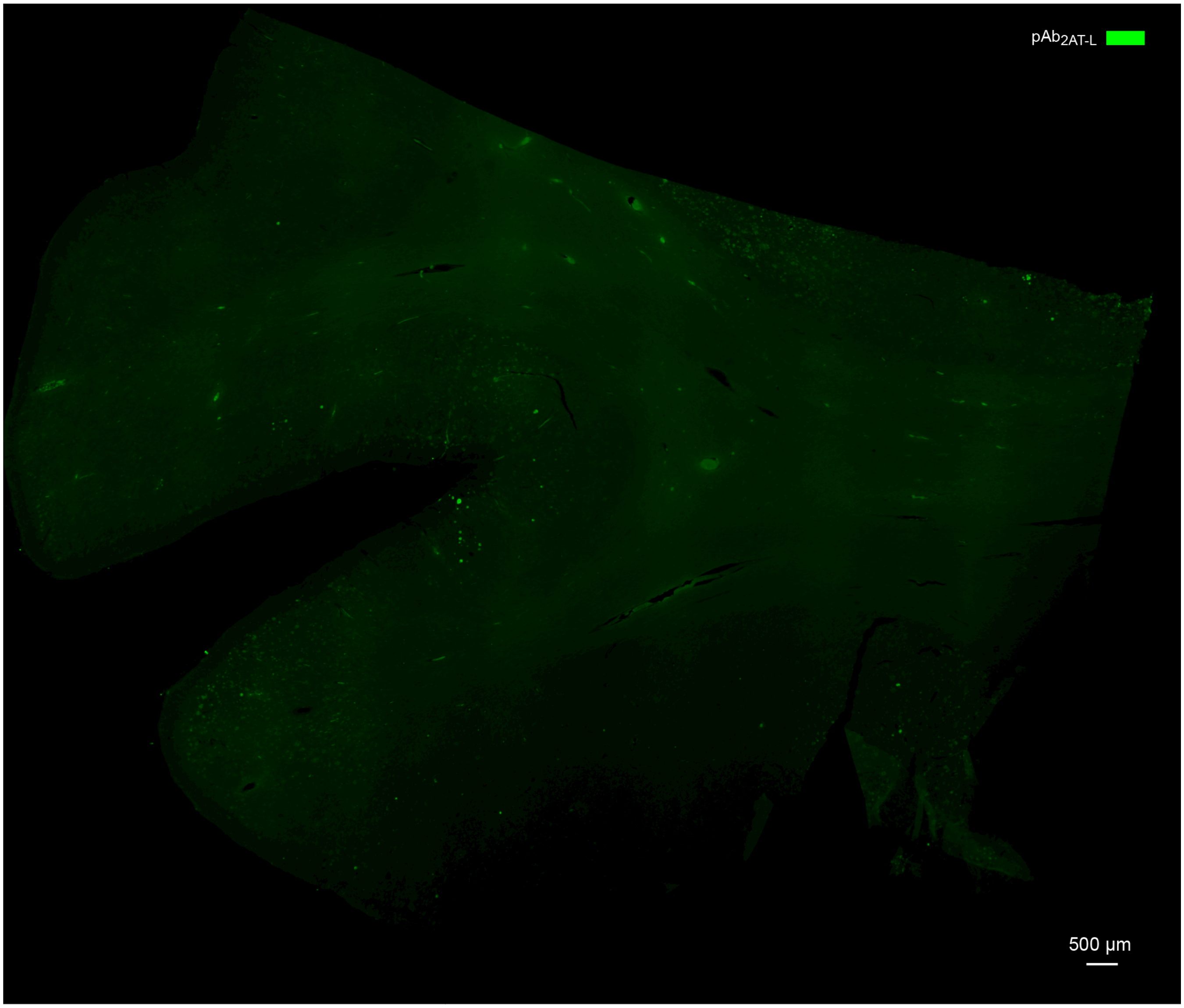

Figure S8. Fluorescence micrograph (4x objective, stitched image) of a frontal cortex brain slice from DSAD individual 2 stained with pAb_{2AT-L} (green).

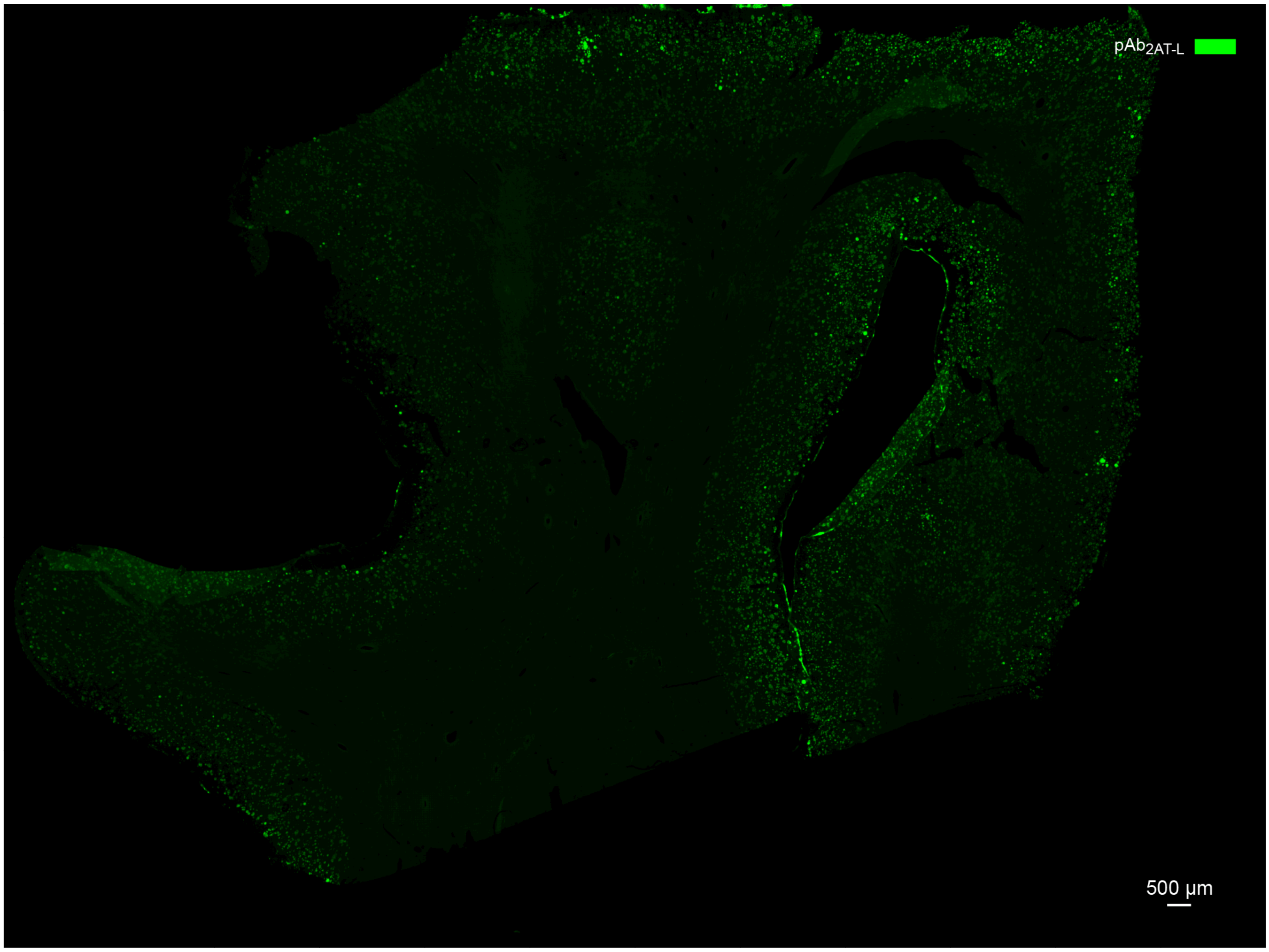

Figure S9. Fluorescence micrograph (4x objective, stitched image) of a frontal cortex brain slice from DSAD individual 3 stained with $pAb_{2AT\text{-}L}$ (green).

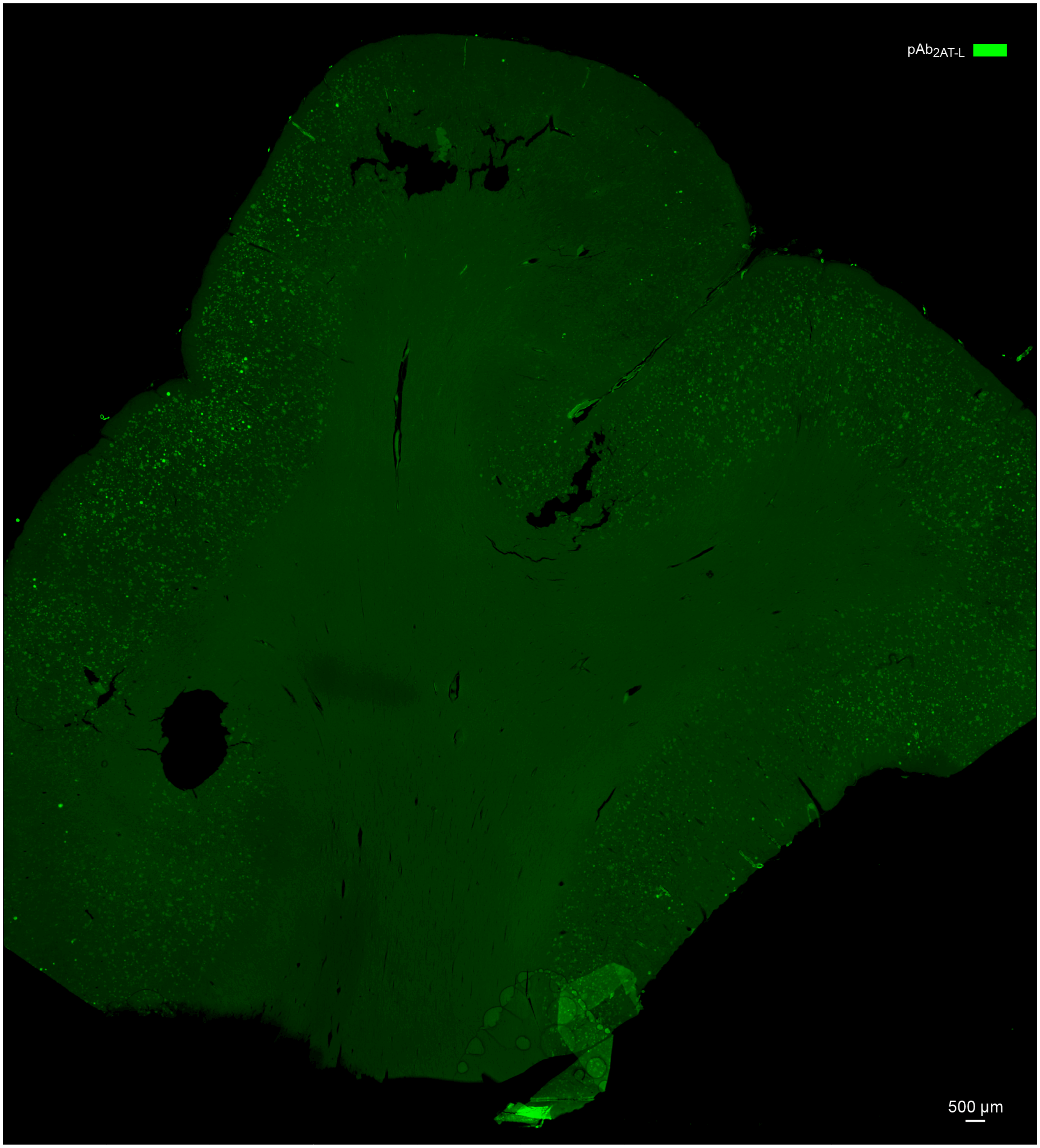

Figure S10. Fluorescence micrograph (4x objective, stitched image) of a frontal cortex brain slice from DSAD individual 4 stained with $pAb_{2AT\text{-}L}$ (green).

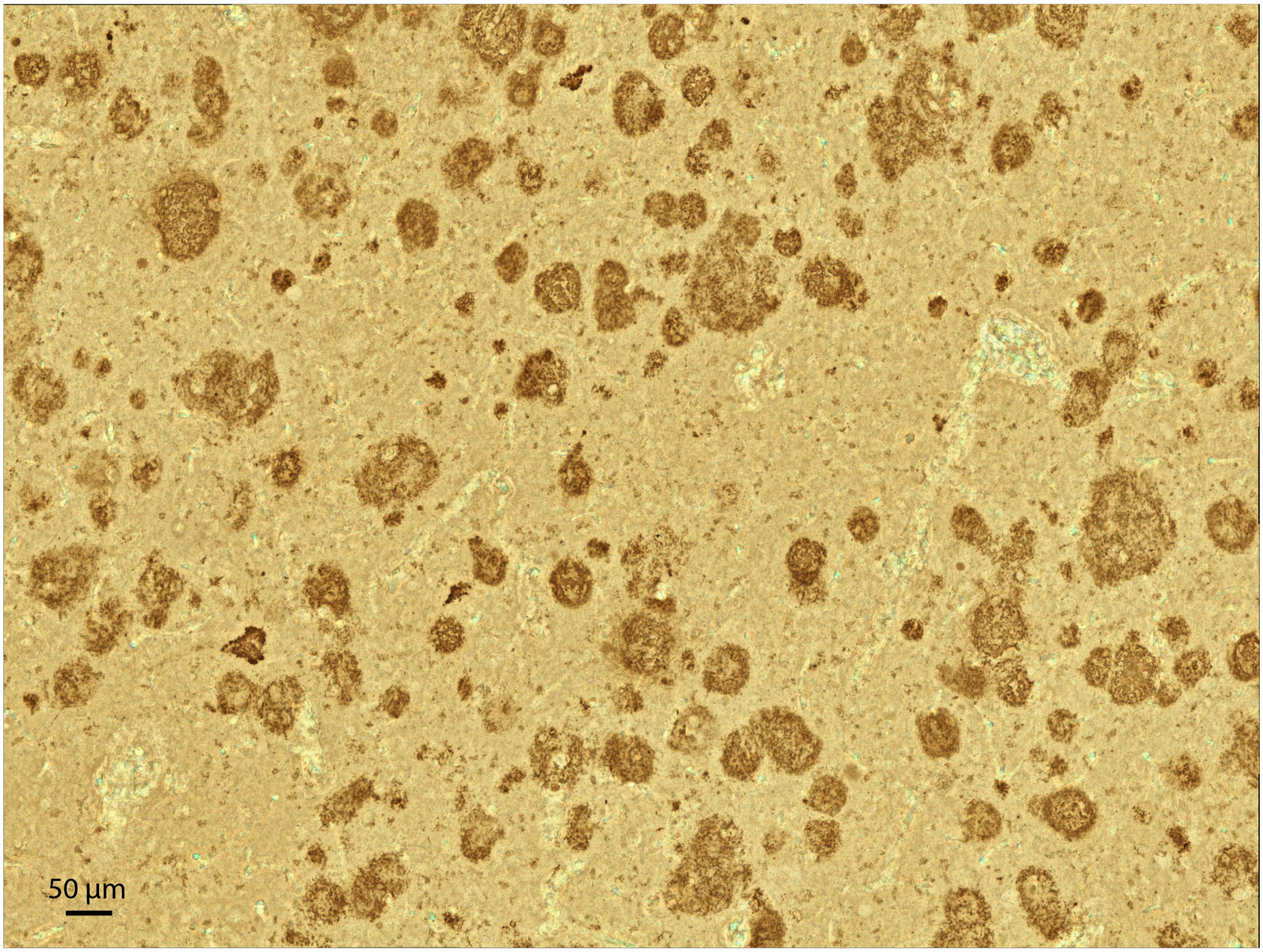

Figure S11. Micrograph (40x objective; stitched image) of a frontal cortex brain slice from DSAD individual 1 DAB stained with $pAb_{2AT\text{-}L}$.

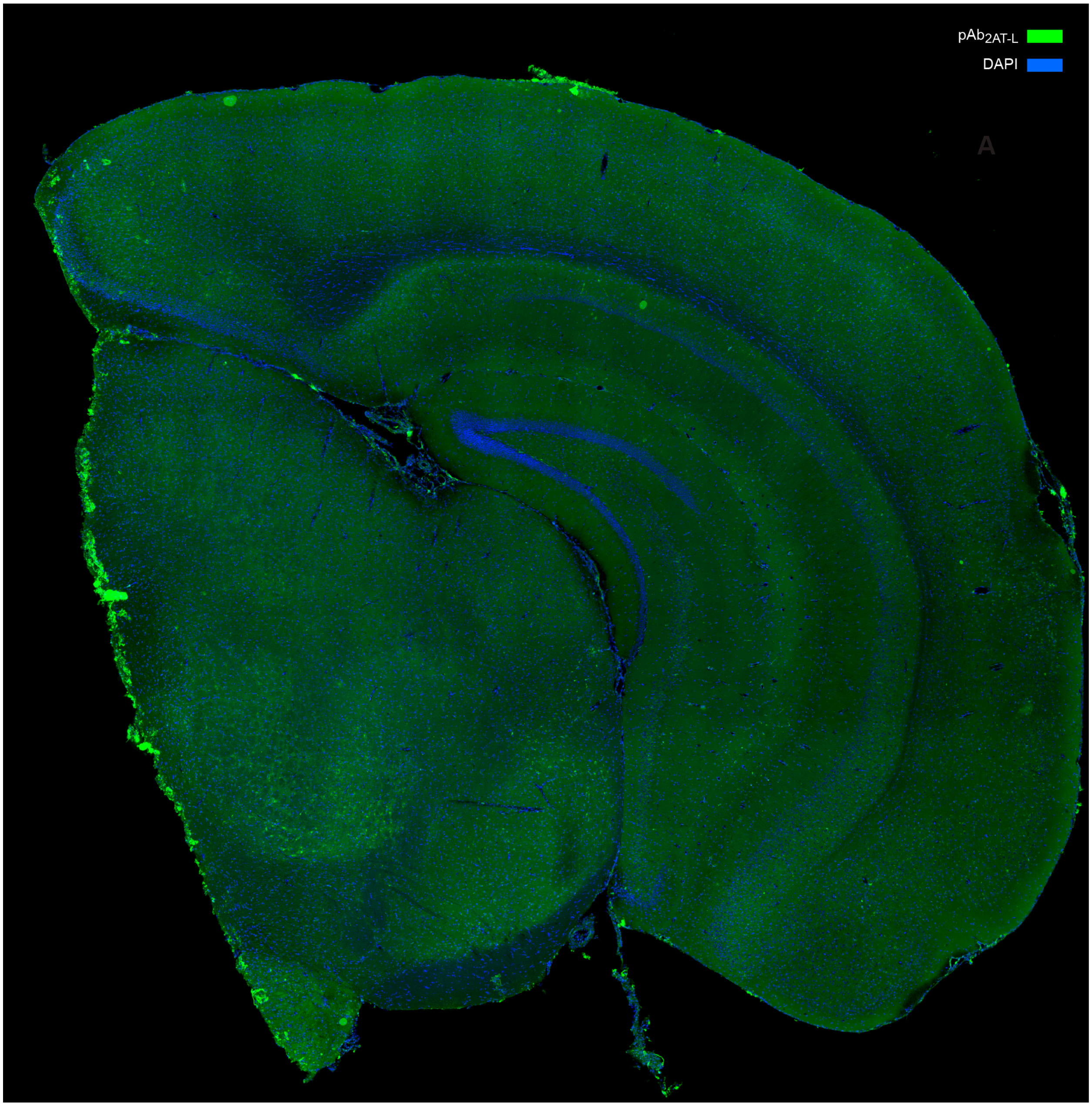

Figure S12. Representative stitched image (10x objective) of a coronal brain section from a 13-month-old female wild type mouse stained with pAb_{2AT-L} (green) and DAPI (blue).

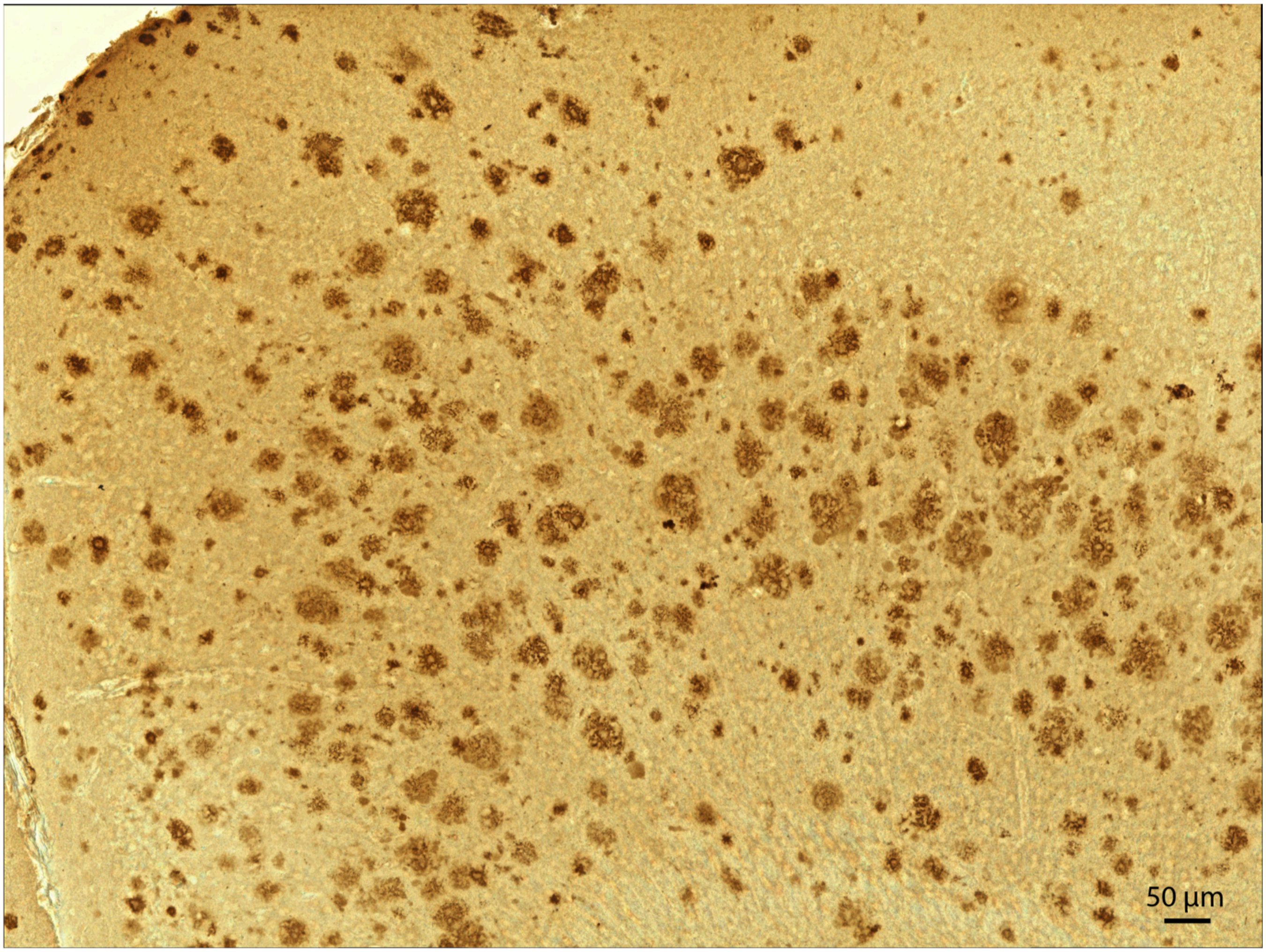

Figure S13. Micrograph (40x objective; stitched image) of a coronal brain slice from an 8-month-old female 5xFAD mouse DAB stained with $pAb_{2AT\text{-}L}$.

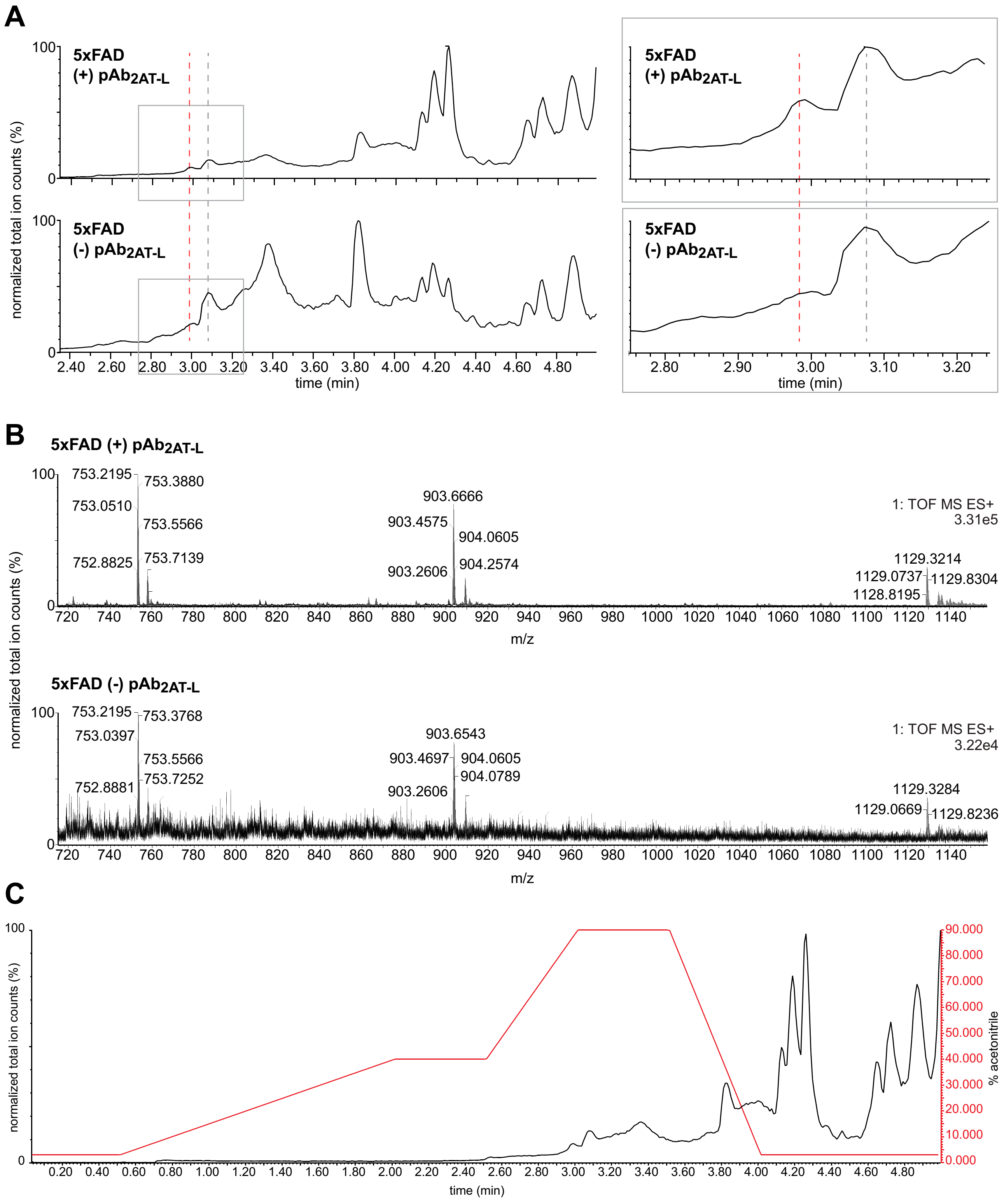

Figure S14. LC-MS data from immunoprecipitation with pAb$_{2AT-L}$ from 5xFAD mouse brain protein extract. ***(A)*** LC-MS chromatograms showing all material pulled down from TBSEd 5xFAD mouse brain protein extract with protein A/G Dynabeads in the presence of pAb$_{2AT-L}$ (+) or absence of pAb$_{2AT-L}$ (-) after elution with 88% formic acid. The red dashed line designates the Aβ$_{42}$ peak. The grey boxed insets are zoomed in chromatograms showing the Aβ$_{42}$ peak ***(B)*** Mass spectra of the Aβ$_{42}$ peaks from (A). ***(C)*** Overlay of the acetonitrile gradient and the full chromatogram from 5xFAD (+) pAb$_{2AT-L}$.

Table S1. Crystallographic properties, crystallization conditions, and data collection and model refinement statistics for 2AT-L.

PDB ID	7U4P
space group	$P6_222$
a, *b*, *c* (Å)	62.054 62.054 47.839
α, β, λ (°)	90, 90, 120
molecules per asymmetric unit	1
wavelength (Å)	0.97741
resolution (Å)	31.03–1.803 (1.868–1.803)
total reflections	194477 (18796)
unique reflections	5373 (522)
multiplicity	36.2 (36.0)
completeness (%)	99.74 (99.81)
mean I/σ(I)	39.93 (4.07)
Wilson B factor	31.27
R_{merge}	0.0643 (0.9299)
$R_{measure}$	0.06524 (0.9431)
$CC_{1/2}$	1 (0.958)
CC^*	1 (0.989)
R_{work}	0.2054 (0.2875)
R_{free}	0.2475 (0.3070)
number of non-hydrogen atoms	484
RMS_{bonds}	0.010
RMS_{angles}	1.00
Ramachandran favored (%)	92.31
Ramachandran allowed (%)	7.69
Ramachandran outliers (%)	0
rotamer outliers (%)	0
clashscore	9.23
average B-factor	56.12
number of TLS groups	6
ligands/ions	0
water molecules	114
crystallization conditions	0.1 M Tris at pH 8.3, 0.2 M $MgCl_2$, 2.8 M 1,6-hexanediol

Materials and Methods[1]

General information

All chemicals were used as received unless otherwise noted. Methylene chloride (CH_2Cl_2) was passed through alumina under nitrogen prior to use. Anhydrous, amine-free *N,N*-dimethylformamide (DMF) was purchased from Alfa Aesar. Deionized water (18 MΩ) was obtained from a Barnstead NANOpure Diamond water purification system. HPLC grade acetonitrile and deionized water, each containing 0.1% trifluoroacetic acid (TFA), were used for analytical and preparative reverse-phase HPLC. 2AM-L, 2AM-L_{CC}, and 2AT-L, were prepared and used as the trifluoroacetate salts and were assumed to have one trifluoroacetic acid molecule per amine group on the peptide. No unexpected or unusually high safety hazards were encountered.

Synthesis, purification, and characterization of 2AM-L and 2AM-L_{CC}.[1]

Loading of the resin. 2-Chlorotrityl chloride resin (300 mg, 1.2 mmol/g) was added to a Bio-Rad Poly-Prep chromatography column (10 mL). The resin was suspended in dry CH_2Cl_2 (10 mL) and allowed to swell for 30 min. The solution was drained from the resin and a solution of Fmoc-Gly-OH (0.50 equiv, 53.5 mg, 0.18 mmol) in 6% (v/v) 2,4,6-collidine in dry CH_2Cl_2 (8 mL) was added immediately and the suspension was gently agitated for 12 h. The solution was then drained and a mixture of CH_2Cl_2/MeOH/*N,N*-diisopropylethylamine (DIPEA) (17:2:1, 10 mL) was added immediately. The mixture was gently agitated for 1 h to cap the unreacted 2-chlorotrityl chloride resin sites. The resin was then washed with dry CH_2Cl_2 (2x) and dried by passing nitrogen through the vessel. This procedure typically yields 0.12–0.15 mmol of loaded resin (0.4–0.5 mmol/g loading).

Peptide coupling. The Fmoc-Gly-2-chlorotrityl resin generated from the previous step was transferred to a peptide synthesis vessel and submitted to cycles of peptide coupling with Fmoc-protected amino acid building blocks. The linear peptide was synthesized from the *C*-terminus of G_{25} to the *N*-terminus of S_{26} (Scheme 1). Each coupling cycle consisted of i. Fmoc-deprotection with 20% (v/v) piperidine in DMF for 5–10 min, ii. washing with DMF (3x), iii. coupling of the amino acid (0.75 mmol,

5 equiv) in the presence of HCTU (0.675 mmol, 4.5 equiv) and 20% (v/v) *N*-methylmorpholine (NMM) in DMF for 30 min, iv. washing with DMF (3x). The Fmoc-Phe-OH that follows the *N*-methyl phenylalanine was double coupled (0.75 mmol, 5 equiv.) in the presence of HATU and HOAT (0.675 mmol, 4.5 equiv) and allowed to react for 1 h per coupling. After coupling of the last amino acid, the terminal Fmoc group was removed with 20% (v/v) piperidine in DMF. The resin was transferred from the peptide synthesis vessel to a Bio-Rad Poly-Prep chromatography column.

Cleavage of the peptide from the resin. The linear peptide was cleaved from the resin by agitating the resin for 1 h with a solution of 1,1,1,3,3,3-hexafluoroisopropanol (HFIP) in CH_2Cl_2 (1:4, 7 mL) in the Bio-Rad Poly-Prep column.[2] The suspension was filtered through the frit of the Poly-Prep column and the filtrate was collected in a 250-mL round-bottomed flask. The resin was washed with additional HFIP in CH_2Cl_2 (1:4, 7 mL) and then with CH_2Cl_2 (2×10 mL). The combined filtrates were concentrated by rotary evaporation to give a white solid. The white solid was further dried by vacuum pump to afford the crude protected linear peptide, which was cyclized without further purification.

Cyclization of the linear peptide. The crude protected linear peptide was dissolved in dry DMF (150 mL). HOBt (114 mg, 0.75 mmol, 5 equiv) and HBTU (317 mg, 0.75 mmol, 5 equiv) were added to the solution. DIPEA (0.33 mL, 1.8 mmol, 12 equiv) was added to the solution and the mixture was stirred under nitrogen for 24 h. The mixture was concentrated under reduced pressure to afford the crude protected cyclic peptide.

Global deprotection of the cyclic peptide. The protected cyclic peptide was dissolved in TFA/triisopropylsilane (TIPS)/H_2O (18:1:1, 20 mL) in a 250-mL round-bottomed flask equipped with a nitrogen-inlet adaptor. The solution was stirred for 1.5 h. The reaction mixture was then concentrated by rotary evaporation under reduced pressure to afford the crude cyclic peptide as a thin yellow film on the side of the round-bottomed flask. The crude cyclic peptide was immediately subjected to purification by reverse-phase HPLC (RP-HPLC), as described below.

Reverse-phase HPLC purification. The crude cyclic peptide was dissolved in H_2O and acetonitrile (7:3, 10 mL), and the solution was filtered through a 0.2 µm syringe filter. The filtrate was injected onto an Agilent Zorbax 300SB-C18 semi-preparative column (21.2 mm x 250 mm, 7 µm particle size) with an Agilent Prep 100Å C18 guard column (21.2 mm x 10 mm) on a Rainin Dynamax HPLC with a flow rate of 20.0 mL/min. The peptides were eluted with a gradient of acetonitrile (20–45% over 90 minutes). Elution was monitored at 214 nm with the accompanying DA Rainin HPLC software. Pure fractions were identified by analytical reverse-phase HPLC using an Agilent 1200 instrument equipped with a Phenomonex Aeris PEPTIDE 2.6u XB-C18 column and then combined and lyophilized to yield pure 2AM-L or 2AM-L_{CC} as fluffy white solids. Syntheses typically yielded 30–40 mg of each peptide as the TFA salt. The lyophilized peptides were analyzed by LC-MS to confirm the purity of the peptide.

Scheme 1. Synthetic scheme for 2AM-L. Synthesis of 2AM-L$_{CC}$ is performed identically, except L$_{17}$ and A$_{21}$ are mutated to cysteine.

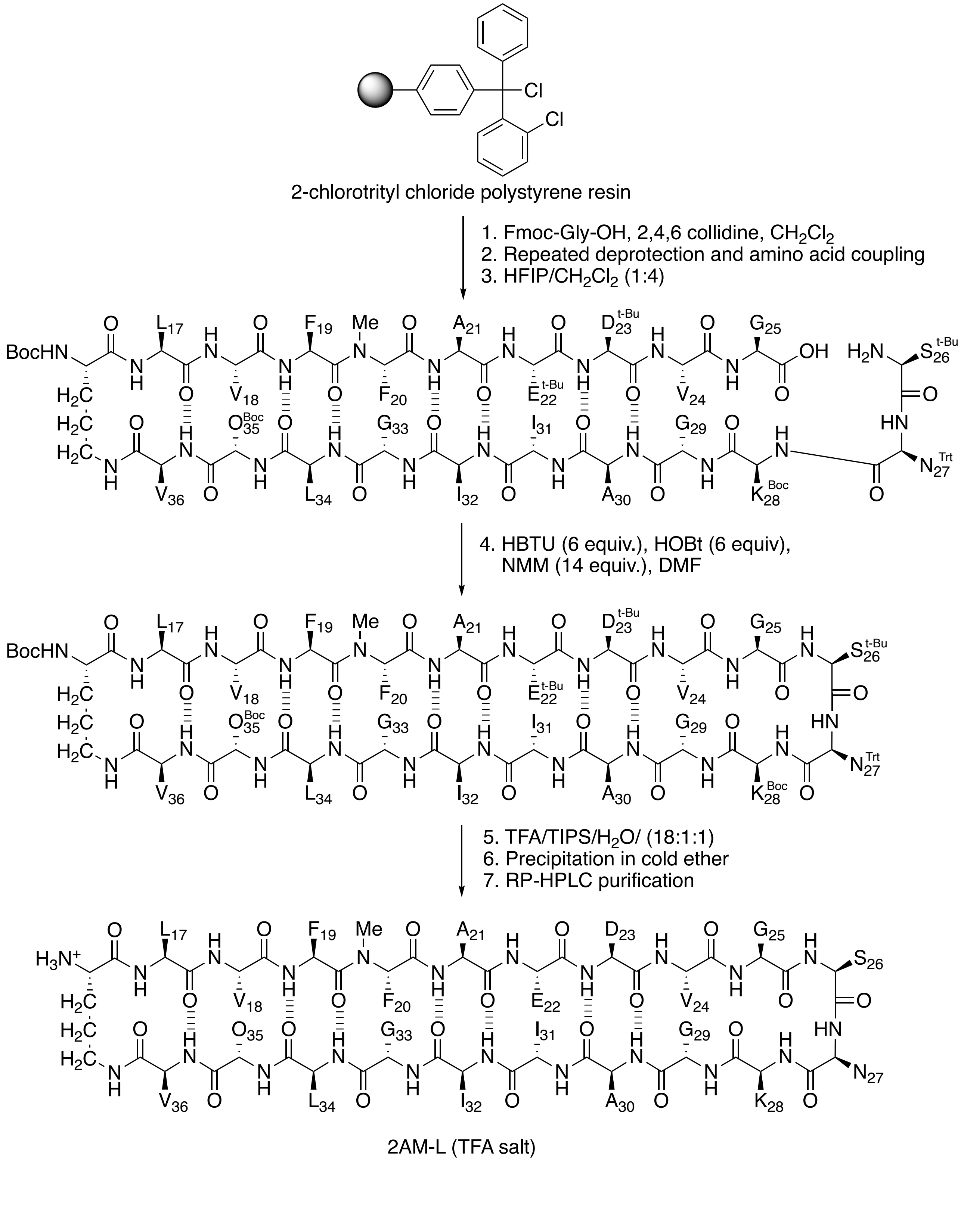

Synthesis, purification, and characterization of 2AT-L.

Synthesis of 2AT-L. 2AT-L was synthesized by oxidizing 2AM-L_{CC} in 20% aqueous DMSO with 60 μM triethylamine (TEA).[3,4] A 6 mM solution of lyophilized 2AM-L_{CC} was prepared gravimetrically by dissolving the peptide in an appropriate volume of deionized water. An appropriate volume of DMSO was then added to achieve a final concentration of 20% (v/v) DMSO. An appropriate volume of TEA was then added to achieve a final concentration of 60 μM TEA. In a representative procedure, 30 mg of the 2AM-L_{CC} TFA salt (0.012 mmol) was dissolved in 1.576 mL of deionized water, and then 0.394 mL of DMSO was added, followed by 16.48 μL TEA. The reaction was carried out in a capped 25 mL glass scintillation vial with gentle swirling (80–90 RPM) on a rotating platform at room temperature for 72 h.

LC-MS analysis of the 2AM-L_{CC} oxidation reaction mixture. LC-MS analysis of the 2AM-L_{CC} oxidation reaction mixture was performed after 72 h on an ACQUITY UPLC H-class system, Xevo G2-XS QTof (Waters Corp.) equipped with a Protein BEH C4 column (300 Å, 1.7 μm, 2.1 mm X 50 mm, Waters Corp). For LC-MS, the oxidation reaction mixture was diluted 100x by combining 1 μL of the oxidation reaction mixture with 199 μL of water. A 5 μL aliquot of the diluted oxidation reaction mixture was injected onto the column and eluted with gradient of Buffer A consisting of 0.1% formic acid in water (Water LC-MS #9831-02, J.T. Baker; Formic Acid LC-MS #85178, Thermo Scientific) and Buffer B, acetonitrile (Acetonitrile UHPLC-MS #A956, Thermo Scientific). Gradient table listed below:

LC-MS elution gradient table (30-minute method)

time (min)	flow rate (mL/min)	%A	%B
initial	0.3	97	3
1.0	0.3	97	3
25.0	0.3	50	50
27.0	0.3	10	90
27.5	0.3	10	90
29.0	0.3	97	3
30.0	0.05	97	3

Purification of 2AT-L. The 2AM-L_{CC} oxidation reaction mixture was subjected to an initial reverse-phase LC purification by directly injecting the reaction mixture onto a Biotage® Isolera One flash chromatography instrument equipped with a Biotage® Isolera Sfär Bio C18 D - Duo 300 Å 20 μm 25 g column. The reaction mixture was injected at 20% aqueous CH_3CN and eluted with a gradient of 20–50% CH_3CN. Fractions containing 2AT-L were identified by LC-MS as described above and then frozen and lyophilized to afford a white powder containing a mixture of products composed of predominantly 2AT-L. The product mixture was then subjected to reverse-phase HPLC purification by dissolving the white powder in 1–3 mL of 20% aqueous CH_3CN and then injecting the solution onto an Agilent Zorbax 300SB-C18 semi-preparative column (21.2 mm x 250 mm, 7 μm particle size) with an Agilent Prep 100 Å C18 guard column (21.2 mm x 10 mm) on a Rainin Dynamax HPLC with a flow rate of 20.0 mL/min and eluted with a gradient of 20–45% CH_3CN over 90 min. In this HPLC purification, the column was submerged in a 60 ºC water bath heated by a thermal immersion circulator. Fractions containing pure 2AT-L were identified by LC-MS using the 30-minute method described above and then combined, frozen, and lyophilized to afford 2AT-L as a white powder. The powder was analyzed by LC-MS in a similar fashion to that described above to confirm the purity of 2AT-L.

In some instances, a significant number of fractions containing 2AT-L from the HPLC purification were contaminated with intramolecular disulfide monomer. In these instances, the intramolecular disulfide monomer (~2.2 kDa) was removed from 2AT-L (~6.6 kDa) by spin filtration as follows: a solution of the product mixture was prepared in 20 mL of 20% aqueous CH_3CN and then centrifuged through a 3 kDa molecular weight cutoff protein concentrator with a PES filter (Pierce™ catalog # 88526) at 3800 x g until the volume in the upper chamber of the spin filter reached 0.5–1 mL. Multiple rounds of centrifugation were performed (typically 3–6 rounds), replenishing the 20 mL of 20% aqueous CH_3CN at the beginning of each round. After the third round, the solution in the upper chamber was analyzed by LC-MS as described above to determine if all intramolecular disulfide monomer had been removed. If intramolecular disulfide monomer remained, additional rounds of centrifugation were performed. Once all the

intramolecular disulfide monomer was removed, the solution in the upper chamber of the spin filter was transferred to a 15 mL conical tube. The reservoir of the upper chamber of the spin filter was rinsed with deionized water and the water rinse was transferred to the 15 mL conical tube. The solution was then frozen in dry ice and lyophilized to yield a fluffy white powder.

Typical syntheses yielded ~5 mg of >95% pure 2AT-L as the TFA salt from a 0.1 mmol scale synthesis of 2AM-L_{CC}.

LC-MS characterization of 2AT-L. The lyophilized 2AT-L was analyzed by LC-MS to confirm the purity of the trimer. LC-MS analysis was performed on an ACQUITY UPLC H-class system, Xevo G2-XS QTof (Waters Corp.) equipped with a Protein BEH C4 column (300 Å, 1.7 µm, 2.1 mm X 50 mm, Waters Corp). For LC-MS, a 10 mg/mL solution of 2AT-L was prepared gravimetrically by dissolving 1.0 mg of the peptide in 100 µL of deionized water. A 0.05 mg/mL solution of 2AT-L was then created by combining 1 µL of the 10 mg/mL solution with 199 µL of deionized water. The 0.05 mg/mL solution of 2AT-L was then further diluted with deionized water to create a 0.005 mg/mL solution. A 5 µL portion of the 0.005 mg/mL 2AT-L solution was injected onto the column and eluted with gradient of Buffer A consisting of 0.1% formic acid in water (Water LC-MS #9831-02, J.T. Baker; Formic Acid LC-MS #85178, Thermo Scientific) and Buffer B, acetonitrile (Acetonitrile UHPLC-MS #A956, Thermo Scientific). Gradient table listed below:

LC-MS elution gradient table (30-minute method)

time (min)	flow rate (mL/min)	%A	%B
initial	0.3	97	3
1.0	0.3	97	3
25.0	0.3	50	50
27.0	0.3	10	90
27.5	0.3	10	90
29.0	0.3	97	3
30.0	0.05	97	3

X-ray crystallography of 2AT-L.[1]

Crystallization procedure for 2AT-L. Initial crystallization conditions were determined using the hanging-drop vapor-diffusion method. Crystallization conditions were screened for 2AT-L using three crystallization kits in a 96-well plate format (Hampton Index, PEG/Ion, and Crystal Screen). Three 150 nL hanging drops that differed in the ratio of peptide to well solution were made per condition in each 96-well plate for a total of 864 experiments. Hanging drops were made by combining an appropriate volume of 2AT-L (10 mg/mL in 18 MΩ water) with an appropriate volume of well solution to create three 150 nL hanging drops with 1:1, 1:2, and 2:1 2AT-L:well solution. The hanging drops were made using a TTP LabTech Mosquito nanodisperse instrument. Crystals of 2AT-L suitable for X-ray diffraction grew in a solution of 0.1 M Tris at pH 8.3, 0.2 M $MgCl_2$, 2.8 M 1,6-hexanediol.

Crystallization conditions for 2AT-L were optimized using a 4x6 matrix Hampton VDX 24-well plate. The pH of Tris was varied in each row in increments of 0.25 pH units (8.05, 8.30, 8.55, and 8.80) and the 1,6-hexanediol concentration in each column in increments of 0.2 M (2.2 M, 2.4 M, 2.6 M, 2.8 M, 3.0 M, 3.2 M). For the first well in the 4x6 matrix we combined 100 μL of 1 M Tris buffer at pH 8.05, 440 μL of 5 M 1,6-hexanediol solution, 100 μL of 1 M $MgCl_2$, and 360 μL of 18 MΩ water. The other wells were prepared in analogous fashion, by combining 100 μL of Tris buffer of varying pH, 1,6-hexanediol in varying amounts, $MgCl_2$, and 18 MΩ water for a total volume of 1 mL in each well.

Three hanging-drops were prepared per borosilicate glass slide by combining a 10 mg/mL solution of 2AT-L (1 μL) and the well solution (1 μL) in a ratio of 1:1, 2:1, and 1:2. Slides were inverted and pressed firmly against the silicone grease surrounding each well. Crystals of 2AT-L grew in ~96 h. Crystals were harvested with a nylon loop attached to a copper or steel pin and flash frozen in liquid nitrogen prior to data collection.

X-ray crystallographic data collection, data processing, and structure determination. Diffraction data for 2AT-L were collected on the Advanced Light Source Synchrotron at the Berkeley Center for Structural Biology on beamline 5.0.1. at 0.97 Å wavelength with 0.5° oscillation. Diffraction data were

collected using b4. Diffraction data were scaled and merged using XDS and pointless and aimless. The resolution was limited to the $CC_{1/2}$ resolution of 1.8 Å. Coordinates for the anomalous signal from *para*-iodo-phenylalanine were determined by HySS in the Phenix software suite 1.10.1. Electron density maps were generated using anomalous coordinates determined by HySS as initial positions in Autosol. Molecular manipulations of the model were performed with Coot. Coordinates were refined in the $C222_1$ space group with phenix.refine. The optimal number and composition of the TLS groups were chosen automatically by phenix.refine.[5,6]

SDS-PAGE and silver staining.[1]

Running buffers for Tricine SDS-PAGE were prepared according to recipes detailed in Schägger, H. *Nat. Protoc.* 2006, *1*, 16–22.[7] 2AT-L and 2AM-L were run on a 16.5% Mini-PROTEAN® Tris-Tricine Gel (Bio-Rad 4563063) with the Spectra™ Multicolor Low Range Protein Ladder (ThermoFisher Scientific, catalog # 26628).

Sample preparation and gel running. Lyophilized 2AT-L and 2AM-L were dissolved in deionized water to a concentration of 10 mg/mL. Aliquots of the 10 mg/mL solutions were then used to create 1.0 mg/mL solutions in 1X SDS-PAGE loading buffer (112.5 mM Tris buffer (pH 8.0) with 2% (w/v) SDS and 6% (v/v) glycerol). The 1.0 mg/mL solutions were then serially diluted in 1X SDS-PAGE loading buffer to create 0.5, 0.25, and 0.125 mg/mL solutions. A 5.0-μL aliquot of each dilution was run on the gel. Once the gels were loaded, the gel-running apparatus was moved to a 4 °C cold room and allowed to equilibrate to 4 °C for 30 min. After equilibration, the gels were run at a constant 30 volts until adequate band separation was observed for the protein ladders.

Silver staining. Staining with silver nitrate was used to visualize 2AT-L and 2AM-L in the gels. Reagents for silver staining were prepared according to procedures detailed in Simpson, R. J. *CSH Protoc.* 2007.[8] [The sodium thiosulfate solution, silver nitrate solution, and developing solution were prepared

fresh each time silver staining was performed. Furthermore, the purity of the sodium carbonate in the developing solution was >99.5%].

For silver staining, the gel was removed from the cast and submerged and rocked in fixing solution (50% (v/v) methanol and 5% (v/v) acetic acid in deionized water) for 20 min. The fixing solution was then discarded and the gel was rocked in 50% (v/v) aqueous methanol for 10 min. The 50% methanol was then discarded and the gel was rocked in deionized water for 10 min. The water was then discarded and the gel was soaked in 0.02% (w/v) sodium thiosulfate in deionized water for 1 min. The sodium thiosulfate was then discarded and the gel was rinsed with deionized water for 1 min (2X). After the last rinse, the gel was submerged in chilled 0.1% (w/v) silver nitrate in deionized water and rocked at 4 °C for 20 min. The silver nitrate solution was then discarded and the gel was rinsed with deionized water for 1 min (2X). To develop the gel, the gel was incubated in developing solution (2% (w/v) sodium carbonate, 0.04% (w/v) formaldehyde until the desired intensity of staining was reached (~1–3 min). When the desired intensity of staining was reached, the development was stopped by discarding the developing solution and submerging the gel in 5% aqueous acetic acid. The silver-stained gels were immediately visualized with a ChemiDoc™ MP Imaging System using the "Optimal Auto-exposure" exposure under the "Silver Stain Gel" application of the Image Lab Touch Software.

Size exclusion chromatography (SEC).[1]

2AT-L and 2AM-L were studied by size exclusion chromatography (SEC) in TBS (50 mM Tris buffer (pH 7.5) and 150 mM NaCl) as follows: Each compound was dissolved in deionized water to a concentration of 10 mg/mL. The 10-mg/mL solutions were then diluted to 1 mg/mL by adding 80 µL of the 10-mg/mL solutions to 720 µL of TBS. The 1-mg/mL solutions were centrifuged at 13,500 RPM for 30 seconds to remove precipitate, and the supernatant was and then loaded onto a GE Superdex 75 10/300 GL column at 0.5 mL/min over 1 min. After loading, the samples were eluted with TBS at 1 mL/min.

Chromatograms were recorded at 214 nm and normalized to the highest absorbance value. The standards Dextran blue, BSA, cytochrome C, aprotinin, and vitamin B_{12} were run in the same fashion.

Dynamic light scattering (DLS).[1]

Dynamic light scattering was measured using a Malvern Zetasizer ZS Nano DLS at ambient temperature (ca. 20ºC). Solutions of 2AT-L and 2AM-L (25 μM) were prepared in 10 mM potassium phosphate buffer at pH 7.4, centrifuged at 17,000 x g for 2 minutes, and then the supernatants were transferred to 1 cm disposable plastic cuvettes. Data were collected in 10 seconds time intervals and averaged over 3 measurements. The scattering was measured with a 173º backscattering angle.

Circular dichroism (CD) spectroscopy.[1]

A 50-μM solution of either 2AT-L or 2AM-L was prepared by diluting an aliquot from a 10 mg/mL stock solution of each compound prepared in deionized water with 10 mM potassium phosphate buffer at pH 7.4. Each solution was transferred to a 1 mm quartz cuvette for data acquisition. CD spectra were acquired on a Jasco J-810 circular dichroism spectropolarimeter at room temperature. Data were collected using 0.2 nm intervals from 260 nm to 190 nm and averaged over five accumulations with smoothing.

Cell-based toxicity assays of 2AT and KLT in SH-SY5Y cells.[1]

The cellular toxicity of 2AT-L and 2AM-L was assessed by measuring LDH release, ATP levels, and caspase-3/7 activation in SH-SY5Y cells exposed to a twofold dilution series (50–1.6 μM, 0 μM) of 2AT-L or 2AM-L for 72 h. LDH release, ATP levels, and caspase-3/7 activation were measured using the following assays:

- LDH release: CyQUANT™ LDH Cytotoxicity Assay (ThermoFisher Scientific; cat# C20301)
- ATP levels: CellTiter-Glo® 2.0 Cell Viability Assay (Promega Corporation; cat. # G9242).

- Caspase-3/7 activation: Apo-ONE® Homogeneous Caspase-3/7 Assay (Promega Corporation; cat. # G7790).

Serial dilutions of 2AT-L and 2AM-L were first prepared in a replica 96-well plate and then transferred to the plate containing cells. Three technical replicates were performed for each experiment.

Preparation of SH-SY5Y cells for the toxicity assays. SH-SY5Y cells were plated in the inner 60 wells (rows B–G, columns 2–10) of cell culture-treated, black-walled, half-area, flat-bottom, clear-bottom 96-well plates (Corning™ cat. # 3882) at 30,000 cells/well. DMEM:F12 media (100 µL) was added to the outer wells (rows A and H and columns 1 and 12), to create an evaporative barrier and ensure the greatest reproducibility of data generated from the inner wells. The cells were plated in 50 µL of a 1:1 mixture of DMEM:F12 media supplemented with 10% fetal bovine serum, 100 U/mL penicillin, and 100 µg/mL streptomycin and incubated at 37 °C in a 5% CO_2 atmosphere for 24 hours to allow the cells to adhere to the bottoms of the wells.

Preparation of 2AT-L and 2AM-L for toxicity assays. 10 mg/mL stock solutions of the 2AT-L and 2AM-L TFA salts were prepared gravimetrically by dissolving 1.0 mg of each compound in 100 µL of deionized water that had been passed through a 0.2 µm filter. The 10 mg/mL solution of the 2AT-L TFA salt is equivalent to 1.25 mM 2AT-L. The 10 mg/mL solution of the 2AM-L TFA salt is equivalent to 3.75 mM 2AM-L. The 10 mg/mL stock solutions were prepared in a 1.7 mL microcentrifuge tube and stored at -20 ºC when not in use.

After the cells had adhered to the bottoms of the wells, the 10 mg/mL stock solutions of each compound were used to create a replica 96-well plate (Corning™ cat. # 353075) containing a twofold dilution series (50–1.6 µM, 0 µM) of 2AT-L and 2AM-L as follows: (1) Add 75 µL of serum-free, Phenol Red-free DMEM:F12 media to the wells in columns 3–11 of rows B–G of the 96-well plate. (2) In the wells of column 2 of rows B–D, prepare 150 µL of a 50 µM solution of 2AT-L by adding 6.0 µL of the 1.25 mM stock solution of 2AT-L to 144.0 µL of serum-free, Phenol Red-free DMEM:F12 media; in the wells of column 2 of rows E–G prepare 150 µL of a 50 µM solution of 2AM-L by adding 2 µL of the 3.75

mM stock solution of 2AM-L to 148 µL of serum-free, Phenol Red-free DMEM:F12 media. (3) using a multi-channel pipette, perform a twofold serial dilution of the trimers by transferring 75 µL of the 50 µM solutions from column 2 to column 3 and mixing by pipetting up and down 8–10 times, and then transferring 75 µL from column 3 to column 4 and mixing, and so on, ending the serial dilution on column 7, with column 8 not receiving any trimer and thus constituting the 0-µM vehicle control.

Treatment of the SH-SY5Y cells with 2AT-L and 2AM-L. A multi-channel pipette was used to remove the media from the wells of the 96-well plate containing cells. A multi-channel pipette was then used to immediately add 50 µL from each well of the 96-well replica plate containing 2AT-L and 2AM-L dilution series to each respective well of the 96-well plate containing cells. The plate was incubated at 37 °C in a 5% CO_2 atmosphere for 72 hours and then the assays were performed according to manufacturer's instructions.

CyQUANT™ LDH Cytotoxicity Assay. After 72 hours, LDH release was measured using a CyQUANT™ LDH Cytotoxicity Assay according to the manufacturer's instructions, except the volumes of assay reagent added to the wells were halved, to accommodate the half-area wells. A 50 µL aliquot of the supernatant media from each well was transferred to a new 96-well plate and 50 µL of LDH substrate solution, prepared according to manufacturer's protocol, was added to each well. The treated plates were stored in the dark for 30 min. The absorbance of each well was measured at 490 nm on a ThermoFisher Scientific Varioskan Lux plate reader. The absorbance measurements for the three replicates of each treatment group were averaged and the standard deviations were calculated using GraphPad Prism. The data were then plotted using GraphPad Prism.

CellTiter-Glo® 2.0 Cell Viability Assay. After 72 hours, ATP levels were measured using a CellTiter-Glo® 2.0 Cell Viability Assay according to the manufacturer's instructions, except the volumes of assay reagent added to the wells were halved, to accommodate the half-area wells. The 96-well plate was removed from the incubator and allowed to come to room temperature for 30 minutes. Once the plate reached room temperature, a 4 mL aliquot of CellTiter-Glo® 2.0 reagent was thawed and then added to a

reagent reservoir (Thermo Scientific cat. # 8093-11). A multi-channel pipette was then used to transfer 50 μL of the CellTiter-Glo® 2.0 reagent to each well containing cells on the 96-well plate. The plate was then shaken on a rotating shaker for 2 minutes at 100 RPM. The luminescence from each well was then measured on a Promega GloMax® Discover Microplate Reader. The luminescence readings for the three replicates of each treatment group were averaged and the standard deviations were calculated using GraphPad Prism. The data were then plotted using GraphPad Prism.

Apo-ONE® Homogeneous Caspase-3/7 Assay. After 72 hours, caspase-3/7 activation was measured using an Apo-ONE® Homogeneous Caspase-3/7 Assay according to the manufacturer's instructions, except the volumes of assay reagent added to the wells were halved, to accommodate the half-area wells. The Apo-ONE® Homogeneous Caspase-3/7 Assay reagents were prepared according to the instructions. The 96-well plate was removed from the incubator and the compound-containing media was removed and replaced with 25 μL of fresh serum-free DMEM/F12 media. A 25-μL aliquot of assay reagent was then added to each well. The plate was sealed with a clear adhesive plate sealer and fluorescence was monitored over 18 h while shaking at 250 rpm using a ThermoFisher Scientific Varioskan Lux plate reader (excitation at 499 nm, emission at 521 nm). The end-point fluorescence readings for the three replicates of each treatment group were averaged and the standard deviations were calculated using GraphPad Prism. The data were then plotted using GraphPad Prism.

Generation of pAb$_{2AT-L}$.

Rabbit immunization. pAb$_{2AT-L}$ was generated by Pacific Immunology (Ramona, CA, www.pacificimmunology.com) using standard custom antibody production procedures. Briefly, 2AT-L was conjugated to the carrier protein keyhole limpet hemocyanin (KLH) using standard EDC conjugation chemistry. The EDC was obtained from Thermo Scientific (catalog # 22980) and the conjugation was performed according to manufacturer's instructions in MES buffer at an acidic pH of 5. We have previously found that at acidic pH, a related covalently stabilized trimer does not assemble to form higher-order

assemblies [*JACS*, **2020**, *142*, 20708]. It is thus likely that 2AT-L does not assemble to form higher-order structures under the conditions of the conjugation reaction to KLH. Two New Zealand white rabbits, 9–10 weeks of age, were used to generate pAb_{2AT-L}. Rabbits were primed with 200 µg of the 2AT-L-KLH conjugate in AdjuLite Complete Freund's Adjuvant (CFA) (Cat. #A5001), by subcutaneous injection using a 19-gauge needle in the neck/shoulder region. On days 21, 42, and 70 the rabbits received booster injections of 100 µg of the 2AT-L-KLH conjugate in AdjuLite Incomplete Freund's Adjuvant (IFA) (Cat. #A5002). Blood was drawn from the central ear artery using a 19-gauge needle on days 0, 49, 63, 77, and 91.

As whole blood was collected, it was diluted in Anticoagulant Citrate Dextrose Solution USP (ACD) Solution A (0.80 g citric acid monohydrate, 2.45 g dextrose monohydrate, and 2.20 g of sodium citrate dihydrate in 100 mL) in a 1:10 ratio. The blood-ACD solution was diluted 1:1 with sterile PBS containing 2.5 mM EDTA and 0.5% BSA. 20 mL of StemCell Lymphoprep was added to a 50-mL StemCell SepMate-50 separation tube, then 30 mL of the blood and ACD solution was added over the Lymphoprep, then centrifuged for 30 minutes at 3,000 RPM. The supernatant (plasma) was retrieved and decanted once the PBMCs have been spun down to form a pellet. The plasma was then used in the affinity purification as described below.

Affinity purification of pAb_{2AT-L}. pAb_{2AT-L} was purified from the plasma using affinity chromatography. To create the affinity chromatography column, 10 mg of the 2AT-L TFA salt was dissolved in 2 mL DMSO. The 2AT-L/DMSO solution was then added to 10 mL PBS (pH 7.4) to create a 1 mg/mL 2AT-L solution in PBS with 20% DMSO. The 1 mg/mL 2AT-L solution was then added to 750 mg dry NHS-activated agarose resin (Thermo Scientific, catalog # 26197) in a 15 mL polypropylene conical tube. The 2AT-L/agarose suspension was then continuously inverted using a tube rotator for two hours at room temperature. After the two-hour incubation, the agarose was transferred to a Bio-Rad Poly-Prep chromatography column (10 mL) and washed 3x with PBS. The agarose was then incubated in 5 mL 1 M Tris buffer (pH 8.5) for 1 hour at room temperature on a tube rotator to cap unreacted NHS-ester groups, and then washed 3x with PBS.

For the affinity purification of pAb_{2AT-L} from the plasma, the 2AT-L-agarose was transferred to a 50 mL CrystalCruz® glass chromatography column. The plasmas from a single production bleed from both rabbits were combined and then filtered through 0.45 μm syringe filters. The filtered plasma was then added to the column containing the 2AT-L-agarose and rocked on a rocker for ~3 hours at room temperature or overnight at 4 ºC. Next, the plasma was drained from the column into a 50 mL polypropylene conical tube, and the 2AT-L-agarose was washed with ice-cold PBS. Washing was performed with 50 mL portions of ice-cold PBS until the absorbance at 214 nm of the wash buffer eluent measured less than 0.05 on a NanoDrop One/One^{c} Microvolume UV-Vis Spectrophotometer.

pAb_{2AT-L} was then eluted from the 2AT-L-agarose using ice-cold 0.2 M glycine buffer (pH 1.85). The pAb_{2AT-L} elution was performed by adding the glycine buffer in 1 mL portions and then collecting the eluent into 1.7 mL microcentrifuge tubes containing 0.5 mL 1 M Tris buffer (pH 8.5). Typically, 10–15 1-mL portions of the glycine buffer were sufficient to elute all of pAb_{2AT-L} from the 2AT-L-agarose. The eluents were then combined and transferred to a 30 kDa molecular weight cutoff filter (30K MWCOF) (Thermo Scientific catalog # 88531) and buffer exchange with PBS was performed. The 30K MWCOF was centrifuged in a swinging bucket centrifuge at 3800 x g until the volume fell below 1 mL, after which the buffer was replenished with ice-cold PBS up to the 20 mL marker on the 30K MWCOF. This process was repeated at least six times. For the final centrifugation step, the pAb_{2AT-L} volume was allowed to reach ~0.5 mL in the 30K MWCOF and was then transferred to a 1.7 mL microcentrifuge tube. The pAb_{2AT-L} concentration was determined using a BCA assay and then adjusted to 1 mg/mL with PBS. Typical affinity purifications yielded 1–3 mg of pAb_{2AT-L} from 50 mL of antisera. The affinity purified pAb_{2AT-L} was then portioned into 50 μL aliquots, which were stored at -80 ºC and thawed as needed. Once thawed, the aliquot was stored at 4 ºC.

Preparation of Aβ_{42}.

A 1 mg portion of recombinantly expressed Aβ_{42} as the ammonium salt was purchased from rPeptide (catalog# A-1167-2) and received as a fluffy lyophilized solid in a glass amber vial. The 1 mg Aβ_{42} portion was dissolved with 1 mL of 2 mM NaOH to create a 1 mg/mL Aβ_{42} solution. The 1 mg/mL Aβ_{42} solution was then sonicated in a water bath sonicator for 5 minutes. After sonication, 0.02 μmol aliquots of Aβ_{42} were prepared by transferring 92.6 μL portions of the 1 mg/mL Aβ_{42} solution to low-binding microcentrifuge tubes (Axygen, catalog# MCT-175-L-C) containing a hole in the lid of the tube created by puncturing the lid with a 22-gauge needle. The aliquots were then frozen on dry ice for 1 hour, transferred to a lyophilization vessel, and lyophilized overnight. The next day, the aliquots were removed from the lyophilizer and each microcentrifuge tube was immediately transferred to its own 50 mL conical tube. The 50 mL conical tubes were sealed by tightening the lid and stored at -80 °C until use.

Indirect ELISAs.

Indirect ELISA was used to determine the selectivity of pAb$_{2AT\text{-}L}$ for 2AT-L and 2AM-L, as well as the negative control protein bovine serum albumin. Indirect ELISA was also used to investigate the binding of pAb$_{2AT\text{-}L}$ and 6E10 to Aβ_{42}. Three technical replicates were performed for each experiment. Each aspiration and washing step in the ELISA procedure was performed using a Fisherbrand™ accuWash™ Microplate Washer (catalog # 14-377-577).

Coating the wells of the ELISA plates. For the ELISA against 2AT-L and 2AM-L, 10 mg/mL stock solutions of the 2AT-L and 2AM-L TFA salts were prepared gravimetrically by dissolving 1.0 mg of each compound in 100 μL of deionized water that had been passed through a 0.2 μm filter. 1 μg/mL solutions of 2AT-L and 2AM-L were then prepared by adding 1 μL of the 10 mg/mL stocks to 10 mL carbonate buffer (15 mM Na_2CO_3, 35 mM $NaHCO_3$, 0.02% (w/v) sodium azide, pH 9.5). The 1 μg/mL solutions of 2AT-L and 2AM-L were then poured into a reagent reservoir and a multichannel pipette was used to transfer 50 μL

to the appropriate wells of a Thermo Scientific™ Maxisorp 96-well plate (catalog# 12-565-135). Each well contained a total of 50 ng 2AT-L or 2AM-L. 2AT-L was added to all wells in columns 1–3 and 2AM-L was added to all wells in columns 4–6. For BSA, a 1% (w/v) solution of BSA was prepared by dissolving 100 mg of BSA (Fraction V) (Fisher BioReagents™, catalog # BP1600-100) in 10 mL carbonate buffer. 50 μL of the 1% BSA solution was then added to all wells in columns 7–9. The 96-well plate was then sealed with an adhesive 96-well plate seal (Axygen, catalog# PCR-SP) and incubated overnight (~16 hours) at room temperature on a rotating shaker set to 90 RPM.

For the ELISA against $A\beta_{42}$, a 0.02 μmol aliquot of $A\beta_{42}$ (prepared as described above) was removed from the -80 °C freezer and allowed to equilibrate to room temperature. The aliquot was then dissolved in 92.6 μL of deionized water to create a 216 μM solution of $A\beta_{42}$. A 1 μM solution of $A\beta_{42}$ in carbonate buffer was then prepared from the 216 μM solution and 50 μL of the 1 μM $A\beta_{42}$ solution was added to the wells of Thermo Scientific™ Maxisorp 96-well plate. The 96-well plate was then sealed with an adhesive 96-well plate seal and incubated overnight (~16 hours) at room temperature on a rotating shaker set to 90 RPM.

Treating the ELISA plates with $pAb_{2AT\text{-}L}$ or 6E10. The next day, 20 mL of 1% BSA was prepared by adding 200 mg of BSA (Fraction V) to 20 mL PBS. The solutions of 2AT-L, 2AM-L, BSA, or $A\beta_{42}$ were aspirated from the wells of the 96-well plate and then washed 1x with PBST (10 mM Na_2HPO_4, 1.8 mM KH_2PO_4, 137 mM NaCl, 2.7 mM KCl, 0.5% Tween-20). A multichannel pipette was then used to transfer 75 μL of 1% BSA to each well, the plate was sealed, and incubated for at least 1 hour at room temperature on a rotating shaker set to 90 RPM to block uncoated sites in the wells. During the last 15 minutes of the blocking step, a 1 μg/mL $pAb_{2AT\text{-}L}$ solution was prepared by combining 1 μL of the 1 mg/mL affinity purified $pAb_{2AT\text{-}L}$ with 999 μL of 1% BSA for the ELISA against 2AT-L and 2AM-L; a 3 μg/mL $pAb_{2AT\text{-}L}$ solution and 3 μg/mL 6E10 solution were prepared analogously for the ELISA against $A\beta_{42}$. After the blocking step, the wells were aspirated and washed 3x with PBST. Using a multi-channel pipette, 50 μL of 1% BSA was then added to the wells in the bottom seven rows of the 96-well plate (rows B–H). 75 μL of the 1 μg/mL

$pAb_{2AT\text{-}L}$ solution was then added to the wells in the top row (row A); for the ELISA against $A\beta_{42}$, 75 μL of the 3 μg/mL $pAb_{2AT\text{-}L}$ and 6E10 solutions were added to their respective wells in the top row (row A). A multi-channel pipette was then used to create a three-fold dilution series of the antibodies by transferring 25 μL of the solutions from row A to row B and then mixing up and down 8 times. 25 μL was then transferred from row B to row C, and so on, until the last 25 μL had been transferred to row H. The 96-well plates were then sealed with an adhesive plate seal and incubated for 2 hours at room temperature on a rotating shaker set to 90 RPM.

Treating the ELISA plate with the secondary antibodies. After the 2-hour incubation, the primary antibody solutions were aspirated and the wells were washed 3x with PBST. A 50 μL portion of AffiniPure Goat Anti-Rabbit IgG (H+L) conjugated to horse radish peroxidase (GαR-HRP; Jackson ImmunoResearch, catalog # 111-035-144) diluted 1:10,000 in 1% BSA was then added to the wells that received $pAb_{2AT\text{-}L}$; a 50 μL portion of AffiniPure Goat Anti-Mouse IgG (H+L) conjugated to horse radish peroxidase (GαM-HRP; Jackson ImmunoResearch, catalog # 115-035-146) diluted 1:10,000 in 1% BSA was added to the wells that received 6E10. The 96-well plate was then sealed with an adhesive plate seal and incubated for 1 hour at room temperature on a rotating shaker set to 90 RPM.

Developing the ELISA plates. After the 1-hour incubation, the secondary antibody solutions were aspirated and the wells were washed 3x with PBST. A 50 μL portion of 3,3',5,5'-tetramethybenzidine (TMB) (Millipore Sigma, catalog # ES001-500ML) was then added to each well and allowed to react until the blue color reached a sufficient hue. A 50 μL portion of 1 M aqueous HCl was then added to each well to quench the reaction and the absorbance was measured at 450 nm using a MultiSkan GO plate reader. The absorbance readings for the three replicates were averaged and the standard deviations were calculated using GraphPad Prism. The data were then plotted and fit using GraphPad Prism to estimate the EC_{50} values.

Western blots.

For the western blot of 2AT-L, a 10 mg/mL solution of 2AT-L in deionized water was prepared as described previously. The 10 mg/mL solution was then used to prepare 40 μL of a 1 mg/mL 2AT-L solution in 1x Tricine Sample Buffer (Bio-Rad, cat. # 1610739). A twofold dilution series (1–0.002 mg/mL) was then created in 1x Tricine Sample Buffer, making 20 μL portions of each dilution.

For the western blot of $A\beta_{42}$, a 0.02 μmol aliquot of $A\beta_{42}$ (prepared as described above) was removed from the -80 °C freezer and allowed to equilibrate to room temperature. The 0.02 μmol aliquot was then dissolved in 26.7 μL deionized water and then 13.3 μL 3x Tricine Sample Buffer to create 40 μL of 0.5 mM $A\beta_{42}$ in 1x Tricine Sample Buffer. The 0.5 mM $A\beta_{42}$ solution was then used to create a twofold dilution series (0.25–0.016 mM) in 1x Tricine Sample Buffer, making 20 μL portions of each dilution.

A 10 μL portion from each dilution of the dilution series was loaded into the lanes of a 16.5% Mini-PROTEAN Tris-Tricine Gel (Bio-Rad, cat. # 4563066). The gel was run at a constant 100 volts until adequate separation was observed for the protein ladders. The gel was then transferred to a 0.2 μm nitrocellulose membrane using the "StandardSD" preprogrammed method on a Bio-Rad Trans-Blot Turbo Transfer System. 1x Trans-Blot Turbo Buffer (Bio-Rad, cat. # 10026938) containing 20% (v/v) methanol was used for the semi-dry transfer.

The membrane was removed from the transfer system, and non-reactive sites were blocked by rocking the membrane in 5% (w/v) non-fat powdered milk in low-Tween TBS (TBS-lT: 50 mM Tris buffer pH 7.4, 150 mM NaCl, 0.01% Tween 20) for 1 hour at room temperature. The membrane was then incubated while rocking overnight at 4 °C in $pAb_{2AT\text{-}L}$ (3 μg/mL) prepared in the 5% milk solution. The next day, the membrane was washed with 3x with TBS-lT for 5 minutes. The $pAb_{2AT\text{-}L}$-exposed membrane was then treated with a solution of Goat Anti-Rabbit IgG (H+L) conjugated to horse radish peroxidase (GαR-HRP; Jackson ImmunoResearch, catalog # 111-035-144) diluted 1:10,000 in 5% milk for 1 hour at room temperature. The membrane was then washed 3x with TBS-lT for 5 min. A 5 mL portion of Thermo Scientific

SuperSignal™ West Pico PLUS Chemiluminescent Substrate (catalog # 34580) was prepared according to the manufacturer's instructions and supplemented with 200 μL Thermo Scientific SuperSignal™ West Femto Maximum Sensitivity Substrate (catalog # 34095). The membrane was incubated in the HRP substrate for ~5 min and then imaged using a Bio-Rad ChemiDoc Gel Imaging System.

After imaging, the membrane was washed 3x in TBS-lT for 5 minutes. The antibodies were then stripped from the membrane by agitating the membrane in stripping buffer (62.5 mM Tris buffer at pH 6.8, 2% (w/v) SDS, and 100 mM 2-mercaptoethanol) for 30 minutes at 37 °C. The membrane was then washed 6x in TBS-lT for 5 minutes, and reblocked in 5% milk for 1 hour. To ensure the antibodies had been stripped, the membrane was treated with a solution of GαR-HRP diluted 1:10,000 in 5% milk for 1 hour at room temperature, and then imaged as described above. The membrane was then washed 3x with TBS-lT for 5 min. The membrane was then rocked for 1 hour at room temperature 6E10 (3 μg/mL) prepared in the 5% milk solution. The membrane was then washed 3x with TBS-lT for 5 min. The 6E10-exposed membrane was treated with a solution of AffiniPure Goat Anti-Mouse IgG (H+L) conjugated to horse radish peroxidase (GαM-HRP; Jackson ImmunoResearch, catalog # 115-035-146) diluted 1:10,000 in 5% milk for 1 hour at room temperature. The membrane was then washed 3x with TBS-lT for 5 min. A 5 mL portion of Thermo Scientific SuperSignal™ West Pico PLUS Chemiluminescent Substrate (catalog # 34580) was prepared according to the manufacturer's instructions and supplemented with 200 μL Thermo Scientific SuperSignal™ West Femto Maximum Sensitivity Substrate (catalog # 34095). The membrane was incubated in the HRP substrate for ~5 min and then imaged using a Bio-Rad ChemiDoc Gel Imaging System.

Time course $A\beta_{42}$ aggregation dot blots.

For the dot blot assays, an aliquot of $A\beta_{42}$ was removed from the -80 °C freezer and allowed to equilibrate to room temperature. The aliquot was then dissolved in 100 μL deionized water followed by 100 μL of 2x PBS to create a 100 μM solution of $A\beta_{42}$ in 1x PBS. The 100 μM $A\beta_{42}$ was then used to create a

twofold dilution series (50–6.25 μM) in PBS in low-binding microcentrifuge tubes (Axygen, catalog# MCT-175-L-C). The 6.25 μM Aβ_{42} solution was then aggregated at room temperature for 96 hours. Over the first five hours of the aggregation period, 1 μL portions of the Aβ_{42} solution were spotted onto 0.2 μm nitrocellulose membranes in triplicate every 30 minutes. The tube was flicked 3–5 times to mix the Aβ_{42} solution before each time point. After the 96-hour time point was spotted, the membrane was allowed to dry for 30 minutes, and then standard immunoblotting was performed as follows.

Non-reactive sites were blocked on each membrane by rocking the membranes in 5% (w/v) non-fat powdered milk in low-Tween TBS (TBS-lT: 50 mM Tris buffer pH 7.4, 150 mM NaCl, 0.01% Tween 20) for 1 hour at room temperature. The membranes were then incubated while rocking overnight at 4 °C in pAb$_{2AT\text{-}L}$ (3 μg/mL) or 4G8 (3 μg/mL) prepared in the 5% milk solution. The next day, the membranes were washed with 3x TBS-lT for 5 minutes. The pAb$_{2AT\text{-}L}$-exposed membranes were then treated with a solution of Goat Anti-Rabbit IgG (H+L) conjugated to horse radish peroxidase (GαR-HRP; Jackson ImmunoResearch, catalog # 111-035-144) diluted 1:10,000 in 5% milk for 1 hour at room temperature; the 4G8-exposed membrane was treated with a solution of AffiniPure Goat Anti-Mouse IgG (H+L) conjugated to horse radish peroxidase (GαM-HRP; Jackson ImmunoResearch, catalog # 115-035-146) diluted 1:10,000 in 5% milk for 1 hour at room temperature. The membranes were then washed 3x with TBS-lT for 5 min. Thermo Scientific SuperSignal™ West Pico PLUS Chemiluminescent Substrate (catalog # 34580) was prepared according to the manufacturer's instructions and supplemented with Thermo Scientific SuperSignal™ West Femto Maximum Sensitivity Substrate (catalog # 34095). A 5 mL portion of the prepared substrate was added to each membrane and the membranes were incubated in the HRP substrate for ~5 min before imaging. The membranes were imaged using a Bio-Rad ChemiDoc Gel Imaging System. The integrated density of each spot was measured in ImageJ. The averages and standard deviations of the three replicates were calculated for each time point in Microsoft Excel. The change in integrated spot density from zero hours was also calculated in Microsoft Excel. The processed data was plotted in GraphPad Prism.

ThT assay on $A\beta_{42}$.

Assay notes: The ThT assay was performed on 6.25 μM $A\beta_{42}$ in PBS at pH 7.4 (10 mM Na_2HPO_4, 1.8 mM KH_2PO_4, 137 mM NaCl, 2.7 mM KCl) containing 10 μM ThT. The assay was performed in triplicate in Corning® 96-well Half Area Black/Clear Flat Bottom Polystyrene NBS Microplates (product# 3881) at 25 °C under quiescent conditions.

A 0.02 μmol aliquot of $A\beta_{42}$ was removed from the -80 °C freezer and allowed to equilibrate to room temperature. During this equilibration time, an 10 μM solution of thioflavin T (ThT) was prepared in PBS. To prepare the 10 μM ThT solution in PBS, a concentrated solution of ThT (1–3 mM) was prepared in a 15 mL polypropylene conical tube by adding ~5–7 mg of ThT (TCI Chemicals, catalog# T0558) to 7–10 mL PBS. The concentrated ThT solution was sonicated in a water bath sonicator for ~5 minutes and then passed through a 0.2 μm nylon syringe filter into a new 15 mL conical tube. The concentration of the concentrated ThT solution was determined photospectrometrically by preparing 2 mL of a 1:200 diluted ThT solution (0.010 mL of the concentrated ThT solution into 1.990 mL PBS). The absorbance of the diluted ThT solution was then measured at 412 nm in a 1 cm quartz cuvette and the concentration was calculated using an estimated extinction coefficient (ε) of 36,000 M^{-1} cm^{-1}. The concentration of the diluted ThT solution was multiplied by 200 to calculate the concentration of the concentrated ThT solution. A 10 mL portion of 10 μM ThT in PBS was prepared by diluting an appropriate volume of the concentrated ThT solution with PBS. The 10 μM ThT solution was then kept on ice until used.

After the 10 μM ThT solution was prepared, a 6.25 μM solution of $A\beta_{42}$ was prepared in 10 μM ThT. To prepare the 6.25 μM solution of $A\beta_{42}$, the equilibrated 0.02 μmol $A\beta_{42}$ aliquot was first dissolved in 92.6 μL deionized water to create a 216 μM $A\beta_{42}$ solution, which was then sonicated in a water bath sonicator for 5 minutes. During the sonication time, 194.21 μL of ice-cold 10 μM ThT was transferred to a 1.7 mL microcentrifuge and kept on ice. After sonication, 5.79 μL of the 216 μM $A\beta_{42}$ solution was added to the

194.21 μL 10 μM ThT to create the 6.25 μM Aβ_{42} solution in ThT and the solution was mixed by pipetting up and down. Three 40 μL portions of the 6.25 μM Aβ_{42} solution were transferred to the wells of the 96-well plate. The plate was sealed with a clear adhesive plate seal (Axygen, catalog# PCR-SP) and was immediately inserted into a ThermoFisher Scientific Varioskan Lux plate reader. Fluorescence measurements of each well were acquired every 2 minutes over a 22-hour period with the following parameters:

excitation: 440 nm	emission: 485 nm
measurement time: 1000 ms	optics: bottom read
excitation bandwidth: 12 nm	

The data were plotted in GraphPad Prism.

Immunostaining and fluorescence microscopy of human and 5xFAD mouse brain slices.

Preparing the human brain tissues for immunostaining. The LOAD, DSAD, and CAA brain tissues used in this project were provided by the University of California Alzheimer's Disease Research Center (UCI-ADRC) and the Institute for Memory Impairments and Neurological Disorders. The human brain tissues were received as blocks of tissue fixed in 4% paraformaldehyde and stored in PBS with 0.02% sodium azide (NaN_3). Upon receipt, the tissue blocks were cryopreserved by soaking the tissue in 15% (w/v) sucrose in PBS with 0.02% NaN_3 in a 50 mL conical tube at 4 ºC until the tissue block sunk to the bottom of the tube, and then in 30% (w/v) sucrose in PBS containing 0.02% NaN_3 in a similar fashion until the tissue block sunk to the bottom of the tube. The tissue blocks were stored at 4 ºC in the 30% sucrose solution until sectioned. For sectioning, the tissue blocks were mounted and frozen in dry ice on the stage of an Epredia™ HM 430 Sliding Microtome (Fisher, catalog # 22-050-855), and then cut into slices with a thickness of 40 μm. The slices were stored at 4 ºC in a 6-well or 12-well dish in PBS with 0.02% NaN_3 until use.

Preparing the 5xFAD mouse brain tissues for immunostaining. 5xFAD mice were bred and genotyped by the Chao Family Comprehensive Cancer Center Transgenic Mouse Facility Shared Resource. The mice were euthanized by intraperitoneal administration of a lethal dose of EUTHASOL® Euthanasia

Solution (pentobarbital sodium and phenytoin sodium). The euthanized mice were transcardiac perfused with ice-cold PBS and their brains were then removed. Using a spatula, the excised brains were cut along the deep longitudinal fissure to separate the two hemispheres. One hemisphere was placed in a 1.7 mL microcentrifuge tube, flash frozen in liquid nitrogen, and then stored at -80 ºC for subsequent biochemical studies. The other hemisphere was drop-fixed in 20 mL of 4% paraformaldehyde in a 20 mL glass scintillation vial and fixed for 96 hours at room temperature for the subsequent immunostaining studies. After fixation, the brain hemisphere was washed 5x with PBS containing 0.02% NaN_3 and then stored in the PBS/azide solution at 4 ºC until use. To prepare the fixed brain hemisphere for sectioning, the tissue was cryopreserved by soaking the tissue in 15% (w/v) sucrose in PBS with 0.02% NaN_3 in the 20 mL glass scintillation vial at 4 ºC until the tissue sunk to the bottom of the tube, and then in 30% (w/v) sucrose in PBS with 0.02% NaN_3 in a similar fashion until the tissue sunk to the bottom of the tube. The brain hemispheres were stored at 4 ºC in the 30% sucrose solution until sectioned. For sectioning, the brain hemispheres were mounted and frozen in dry ice on the stage of an Epredia™ HM 430 Sliding Microtome (Fisher catalog # 22-050-855), and then cut into coronal slices with a thickness of 40 µm. The slices were stored at 4 ºC in a 12-well or 24-well dish in PBS with 0.02% NaN_3 until use.

Immunofluorescence staining and imaging of the human and 5xFAD mouse brain tissue slices. The human and mouse brain tissue slices were immunostained with $pAb_{2AT\text{-}L}$ in an identical manner. All washing, soaking, and incubation steps were performed at room temperature on a rotating platform set to 70 RPM. In some instances, the tissue slices were also stained with the anti-Aβ antibody 6E10, or the amyloid-staining dyes AmyTracker480 (AT480) and AmyTracker680 (AT680).

antibody and dye information for immunostaining

antibody	host animal	source	working concentration
$pAb_{2AT\text{-}L}$	rabbit	custom made	3 µg/mL
6E10	mouse	Biolegend (catalog # 803015)	3 µg/mL
Goat anti-Rabbit IgG Alexa Fluor™ Plus 488	goat	Invitrogen (catalog # A32731)	2 µg/mL
Goat anti-Mouse IgG Alexa Fluor™ Plus 647	goat	Invitrogen (catalog # A32728)	2 µg/mL

dye	source	dilution
AmyTracker480 (AT480)	Ebba Biotech (catalog # A680)	1:1000
AmyTracker680 (AT680)	Ebba Biotech (catalog # A480)	1:1000
DAPI	Thermo Scientific™ (catalog # 62248)	1:1000

To begin the immunostaining procedure, the tissue slices were transferred from the storage plates to the wells of clean 12- or 24-well plates containing TBS (50 mM Tris buffer pH 7.4, 150 mM NaCl). The tissue slices were then washed 3x with TBS for 5 minutes. Antigen retrieval was then performed by soaking the tissues in 88% aqueous formic acid for 5 minutes, followed by washing the tissue 3x with TBS for 5 minutes. Permeabilization was then performed by incubating the tissues in TBSA (TBS with 0.1% Triton-X) for 30 minutes. The tissue slices were then blocked by incubating the tissues in TBSB (TBSA with 2% bovine serum albumin Fraction V) for 1 hour. In instances where mouse tissue slices were stained with 6E10, which has a mouse IgG1 isotype, M.O.M. ® (Mouse on Mouse) Blocking Reagent (vector laboratories, catalog # MKB-2213-1) was added to the TBSB used in the blocking step. The blocking solution was then removed and replaced with a primary antibody solution prepared in TBSB. The primary antibodies were used at the concentrations listed in the table above. The tissue slices were incubated in the primary antibody solution overnight (~16 hours) at room temperature. The next day, the primary antibody solutions were removed and the tissue slices were washed 3x with TBSA for 5 minutes. The tissue slices were then re-blocked by incubating the tissues in TBSB containing 5% normal goat serum (Gibco™, catalog # PCN5000) for 30 minutes. The blocking solution was then removed and replaced with an appropriate secondary antibody solution prepared in TBSB with 5% normal goat serum. The secondary antibodies were used at the dilutions

listed in the table above. The tissue slices were incubated in the secondary antibody solution for 2–3 hours. The secondary antibody solutions were then removed and the tissue slices were washed 3x with TBS for 5 minutes. In instances where the AmyTracker dyes were used, the tissue slices were then incubated in a solution of AmyTracker dye prepared in TBS at a 1:1000 dilution for 30 minutes. The dye solution was then removed, and the tissue slices were washed 2x in TBS for 2 minutes. In instances where DAPI was used, the tissue slices were then incubated in a solution of DAPI prepared in TBS at a 1:1000 dilution for 30 minutes. The tissue slices were then washed 3x with TBS for 5 minutes and then mounted on glass slides and cover-slipped. The tissue slices were either imaged immediately or were placed in a slide storage box and stored at 4 ºC until imaged. The stained brain slices were imaged using a Zeiss LSM 900 with Airyscan 2 confocal microscope, or a Keyence BZ-X800 fluorescent microscope. The images in Figures 3, 4, and 5 were acquired on the Zeiss LSM 900 confocal microscope using a 10x, 20x, or 63x objective; the images in Figures S3–S9 were acquired on the BZ-X800 fluorescent microscope using a 4x or 10x objective. Plaque classification in the human brain tissue slices was performed based on morphological analysis of the plaques as well as the chemical and immunological staining properties of the plaques.

DAB staining and imaging of the human and 5xFAD mouse brain tissue slices. The human and mouse brain tissue slices were immunostained with $pAb_{2AT\text{-}L}$ in an identical manner. All washing, soaking, and incubation steps were performed at room temperature on a rotating platform set to 70 RPM.

To begin the immunostaining procedure, the tissue slices were transferred from the storage plates to net wells in a clean 12-well plate containing TBS (50 mM Tris buffer pH 7.4, 150 mM NaCl). The tissue slices were then washed 3x with TBS for 5 minutes. Antigen retrieval was then performed by soaking the tissues in 88% aqueous formic acid for 5 minutes, followed by washing the tissue 3x with TBS for 5 minutes. Endogenous peroxidases were quenched by soaking the tissue in 3% H_2O_2 with 10% MeOH for 30 minutes. The tissue slices were then washed 3x with TBS for 5 min. Permeabilization was then performed by incubating the tissues in TBSA (TBS with 0.1% Triton-X) for 30 minutes. The tissue slices were then blocked by incubating the tissues in TBSB (TBSA with 2% bovine serum albumin Fraction V) for 1 hour. The

blocking solution was then removed and replaced with pAb_{2AT-L} (3 μg/mL) prepared in TBSB. The tissue slices were incubated in the primary antibody solution overnight (~16 hours). The next day, pAb_{2AT-L} was removed and the tissue slices were washed 3x with TBSA for 5 minutes. The tissue slices were then re-blocked by incubating the tissues in TBSB containing 5% normal horse serum (Vector Laboratories, REF S-2000) for 30 minutes. The blocking solution was then removed and replaced with horse anti-rabbit biotinylated secondary antibody (1:1000) (Vector Laboratories, BA-1100-1.5) prepared in TBSB with 5% normal horse serum. The tissue slices were incubated in the secondary antibody solution for 1 hour. The secondary antibody solution was then removed and the tissue slices were washed 3x with TBS for 5 minutes. The tissue slices were then placed in ABC solution (Vectastain Elite ABC-HRP Kit, Peroxidase, Cat. No. PK-6100) for 1 hour. The tissue was then washed 3x in TBS for 5 minutes. During the last of the three washes, the DAB (3, 3 -diaminobenzidine) reagent was prepared in the fume hood and according to manufacturer's instructions (DAB Substrate Kit, Peroxidase (HRP), with Nickel, Cat. No. SK-4100). The tissue was then incubated in DAB for 5 minutes. The tissue slices were washed 3x with TBS for 5 minutes and then mounted on glass slides and cover-slipped. The tissue slices were imaged using a Keyence BZ-X800 fluorescent microscope using a 40x objective.

Preparation of 5xFAD brain protein extracts.

The protocol for isolating pAb_{2AT-L}-reactive species was adapted from a protocol first described by Ashe and co-workers. Frozen brain hemispheres from 8-month-old 5xFAD and wild type mice, isolated and stored as described above, were thawed on ice. A 250 μL portion of ice-cold TBSE (50 mM Tris buffer pH 7.4, 150 mM NaCl, and 1 mM EDTA with Halt™ Protease Inhibitor Cocktail (Thermo Scientific, catalog # 78430)) was then added to the thawed brain tissue. The tissue was then passed through a 20-gauge needle attached to a 1 mL syringe 8 times to mechanically homogenize the tissue. The tissue homogenate was then centrifuged at 17,000 x g in a tabletop centrifuge at 4 ºC for 90 minutes. After centrifugation, the TBSE-soluble protein supernatant was collected in a new 1.7 mL microcentrifuge tube and stored at -20 ºC

overnight. A 300 μL portion of ice-cold TBSEd (TBSE with 0.5% Triton-X, 3% (w/v) SDS, and 1% (w/v) sodium deoxycholate) was then added to the tissue pellet. The tissue pellet was then passed through a 1 mL pipette tip attached to a pipette until the suspension smoothly moved through the pipette tip (about 10 x). The tissue pellet homogenate was then centrifuged at 17,000 x g in a tabletop centrifuge at 4 ºC for 90 minutes. After centrifugation, the TBSEd-soluble protein supernatant was collected in a new 1.7 mL microcentrifuge tube and stored at -20 ºC overnight.

The next day, the TBSE and TBSEd protein extracts were removed from the -20 ºC freezer and thawed on ice. The extracts were then centrifuged at 17,000 x g in a tabletop centrifuge at 4 ºC for 90 minutes to remove any precipitate. During centrifugation, two 50 μL portions of Protein A/G Magnetic Beads (Pierce, catalog # 88802) were washed 5 x with TBS. The extracts were then added to the washed Protein A/G beads and incubated on a tube rotator at 4 ºC for 1 hour to immunodeplete the extracts. The Protein A/G beads were then removed and the extracts were transferred to new 1.7 mL microcentrifuge tubes. The total protein concentration in each extract was then quantified using a Pierce™ BCA Protein Assay Kit (Thermo Scientific™, catalog # 23225). The extracts were then aliquoted into portions containing 250 µg total protein and stored at -80 ºC until use.

Dot blot assay of pAb$_{2AT-L}$ against 5xFAD brain protein extracts.

The 5xFAD and wild type brain protein extracts were removed from the -80 ºC freezer and thawed on ice. The concentration of each extract was adjusted to 1 mg/mL with ice-cold TBSE or TBSEd. A twofold dilution series (1–0.13 mg/mL) for each extract was then created by diluting the 1 mg/mL protein extracts with ice-cold TBSE or TBSEd. A 1 μL portion from each concentration in the dilution series for each extract was then spotted onto a nitrocellulose membrane, and the membrane was allowed to air-dry for 10 minutes. Three identical membranes were created: one to be probed with pAb$_{2AT-L}$, one to be probed with 6E10, and one to be probed with goat anti-rabbit IgG HRP. Non-reactive sites were blocked on each membrane by rocking the membranes in 5% (w/v) non-fat powdered milk in low-Tween TBS (TBS-lT: 50 mM Tris buffer

pH 7.4, 150 mM NaCl, 0.01% Tween 20) for 1 h at room temperature. The membranes were then incubated while rocking overnight at 4 °C in pAb_{2AT-L} (3 μg/mL) or 6E10 (3 μg/mL) prepared in 5% milk solution. The third blot was incubated in a 5% milk solution containing no primary antibody and would serve as the goat anti-rabbit "secondary only" negative control.

The next day, the membranes were washed with 3x TBS-lT for 5 minutes. The pAb_{2AT-L} membrane and the "secondary only" membrane were then treated with a solution of goat anti-rabbit IgG HRP antibody (0.8 μg/mL) (Jackson ImmunoResearch catalog# 111-035-003); the 6E10 membrane was treated with a solution of goat anti-mouse IgG HRP antibody (0.8 μg/mL) (Jackson ImmunoResearch catalog# 115-035-062) for 1 hour at room temperature. The membranes were then washed 3x with TBS-lT for 5 min. A 20 mL portion of chemiluminescent HRP substrate (Thermo Scientific SuperSignal™ West Pico PLUS Chemiluminescent Substrate, catalog # 34580) was prepared according to the manufacturer's instructions and then 5 mL of the prepared substrate was added to each membrane. The membranes were incubated in the HRP substrate for ~5 min before imaging. The blot was imaged using a Bio-Rad GelDoc Go Gel Imaging System using the "Auto-expose" option of the Bio-Rad Image Lab Touch Software.

Immunoprecipitation LC-MS on 5xFAD brain protein extracts.

Two 250 μg 5xFAD brain protein extract aliquots were removed from the -80 ºC freezer and thawed on ice. The volume of each brain protein extract was then adjusted to 1 mL with ice-cold TBS. The brain protein extracts were then immunodepleted once more by adding 50 μL of washed Protein A/G magnetic beads to each extract and then incubating the samples at 4 ºC on a tube rotator for 1 hour. The Protein A/G magnetic beads were then removed and 5 μg of pAb_{2AT-L} was added to one of the extracts. No antibody was added to the other extract, as it will serve as the (-) pAb_{2AT-L} control. A 50 μL portion of washed Protein A/G magnetic beads was then added to each extract and the samples were incubated over night at 4 ºC on a tube rotator. The next day, the magnetic beads were separated from the extracts using a magnet and washed 5x with ice cold TBS. The bound contents were eluted from the Protein A/G magnetic

beads by incubating the beads in 50 µL 88% aqueous formic acid at room temperature for 5 minutes. The formic acid was then evaporated on a SpeedVac centrifuge and the remaining material was redissolved in 20 µL 20% aqueous acetonitrile containing 0.1% formic acid. The redissolved material was then analyzed by LC-MS. LC-MS was performed on an ACQUITY UPLC H-class system, Xevo G2-XS QTof (Waters Corp.) equipped with a Protein BEH C4 column (300 Å, 1.7 µm, 2.1 mm X 50 mm, Waters Corp). For LC-MS, a 5 µL portion of the redissolved material was injected onto the column and eluted with gradient of Buffer A consisting of 0.1% formic acid in water (Water LC-MS #9831-02, J.T. Baker; Formic Acid LC-MS #85178, Thermo Scientific) and Buffer B, acetonitrile (Acetonitrile UHPLC/MS #A956, Thermo Scientific). Gradient table listed below:

LC/MS elution gradient table (5 minute method)

time (min)	flow rate (mL/min)	%A	%B
initial	0.3	97	3
0.5	0.3	97	3
2	0.3	40	60
2.5	0.3	40	60
3	0.3	10	90
3.5	0.3	10	90
4	0.3	97	3
5	0.3	97	3

The Aβ_{42} peak was identified by averaging the spectrum of each peak in the LC trace and looking for mass to charge ratios that correspond to Aβ_{42}. The $[M+4H]^{4+}$, $[M+5H]^{5+}$, and $[M+6H]^{6+}$ for Aβ_{42} were the most predominant mass to charge ratios for the peak labeled Aβ_{42} in Figure S14. These mass to charge ratios were not found for any other peak in the LC trace.

References and Notes

1 These procedures follow closely those that our laboratory has previously published. The procedures in this section are adapted from and in some cases taken verbatim from:

A. G. Kreutzer, S. Yoo, R. K. Spencer, J. S. Nowick, Stabilization, Assembly, and Toxicity of Trimers Derived from Aβ. *J. Am. Chem. Soc.* **139**, 966–975 (2017).

A. G. Kreutzer, I. L. Hamza, R. K. Spencer, J. S. Nowick, X-ray Crystallographic Structures of a Trimer, Dodecamer, and Annular Pore Formed by an Aβ17–36 β-Hairpin. *J. Am. Chem. Soc.* **138**, 4634–4642 (2016).

R. K. Spencer, A. G. Kreutzer, P. J. Salveson, H. Li, J. S. Nowick, X-ray Crystallographic Structures of Oligomers of Peptides Derived from β2-Microglobulin. *J. Am. Chem. Soc.* **137**, 6304–6311 (2015).

R. K. Spencer, H. Li, J. S. Nowick, X-ray crystallographic structures of trimers and higher-order oligomeric assemblies of a peptide derived from Aβ(17–36). *J. Am. Chem. Soc.* **136**, 5595–5598 (2014).

G. Guaglianone, *et al.*, Elucidating the Oligomerization and Cellular Interactions of a Trimer Derived from Aβ through Fluorescence and Mass Spectrometric Studies. *ACS Chem. Neurosci.* **13**, 2473–2482 (2022).

2 Bollhagen, R.; Schmiedberger, M.; Barlosb, K.; Grell, E. A new reagent for the cleavage of fully protected peptides synthesised on 2-chlorotrityl chloride resin. *J. Chem. Soc., Chem. Commun.* **1994**, 2559–2560.

3 J. P. Tam, C. R. Wu, W. Liu, J. W. Zhang, Disulfide bond formation in peptides by dimethyl sulfoxide. Scope and applications. *J. Am. Chem. Soc.* **113**, 6657–6662 (1991).

4 O. Khakshoor, J. S. Nowick, Use of Disulfide "Staples" To Stabilize β-Sheet Quaternary Structure. *Org. Lett.* **11**, 3000–3003 (2009).

5 Painter, J.; Merritt, E. A. Optimal description of a protein structure in terms of multiple groups undergoing TLS motion. *Acta Crystallogr D Biol Crystallogr.* **2006**, *62*, 439–450.

6 Painter, J.; Merritt, E. A. TLSMD web server for the generation of multi-group TLS models. *J. Appl. Cryst.* **2006**, *39*, 109–111.

7 H. Schägger, Tricine–SDS-PAGE. *Nat. Protoc.* **1**, 16–22 (2006).

8 R. J. Simpson, Staining proteins in gels with silver nitrate. *CSH Protoc.* **2007**, db.prot4727 (2007).

9 S. Lesné, *et al.*, A specific amyloid-beta protein assembly in the brain impairs memory. *Nature* **440**, 352–357 (2006).

Characterization Data

Characterization of 2AM-L

LC-MS of 2AM-L

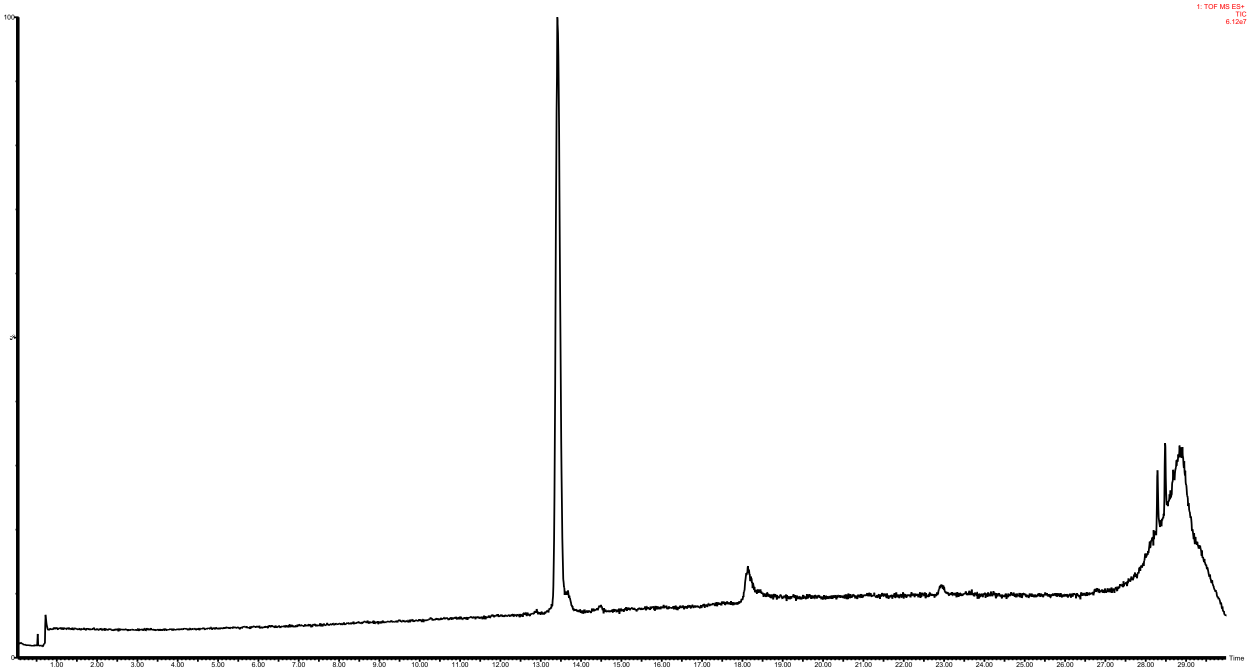

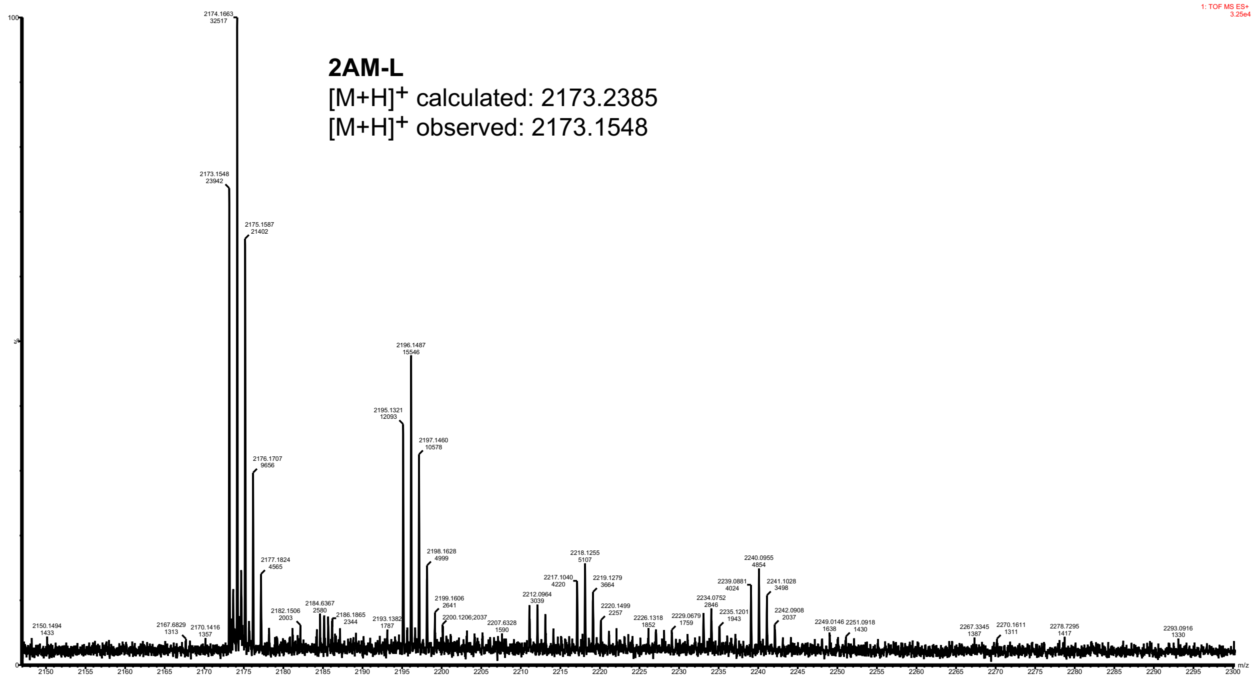

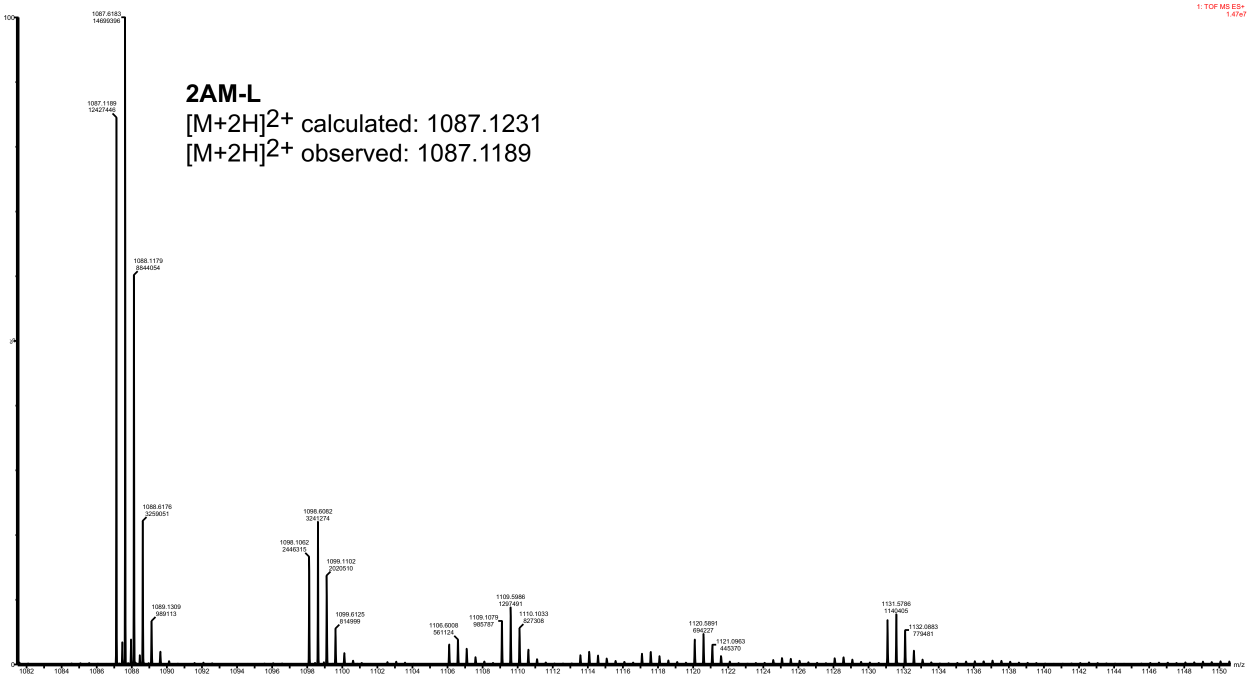
2AM-L
[M+2H]2+ calculated: 1087.1231
[M+2H]2+ observed: 1087.1189

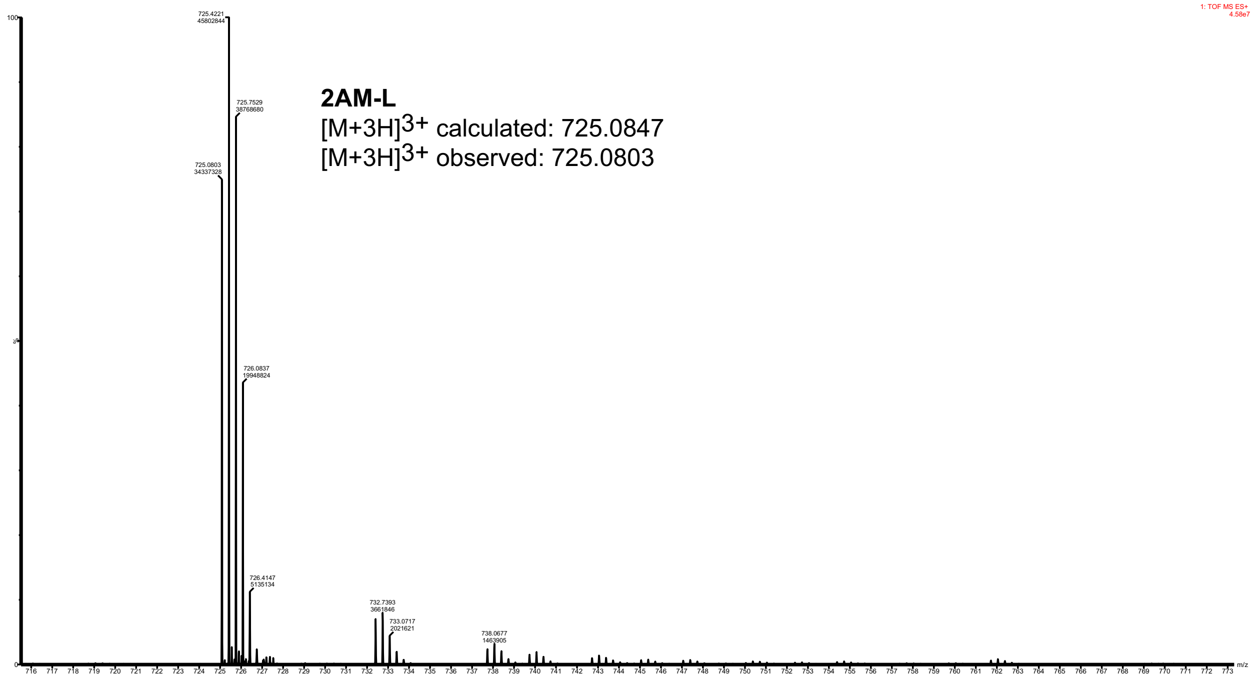
2AM-L
[M+3H]3+ calculated: 725.0847
[M+3H]3+ observed: 725.0803

Characterization of 2AT-L

LC-MS of 2AT-L

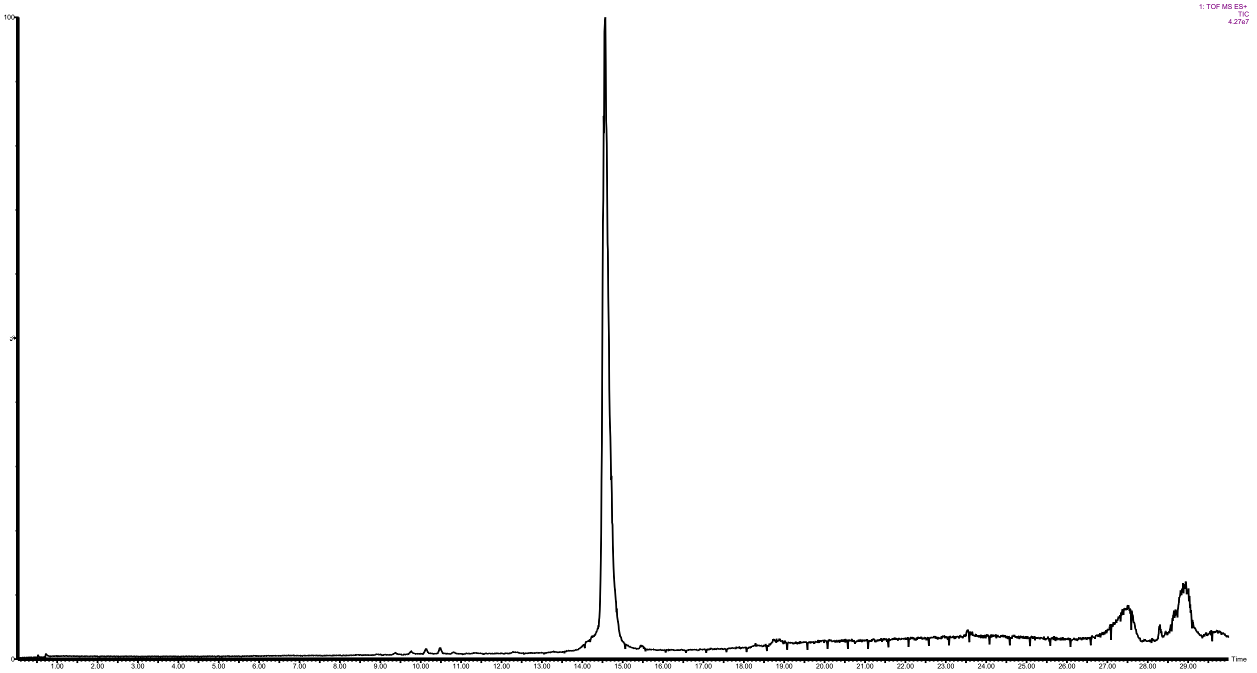

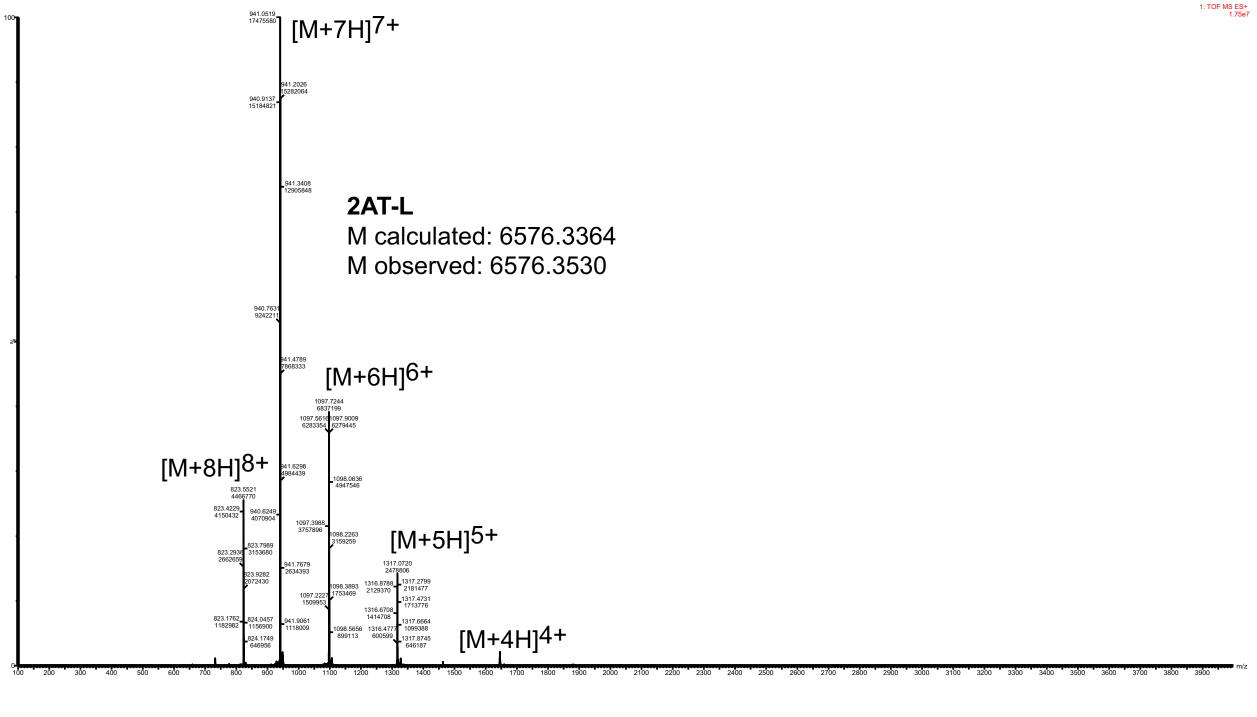

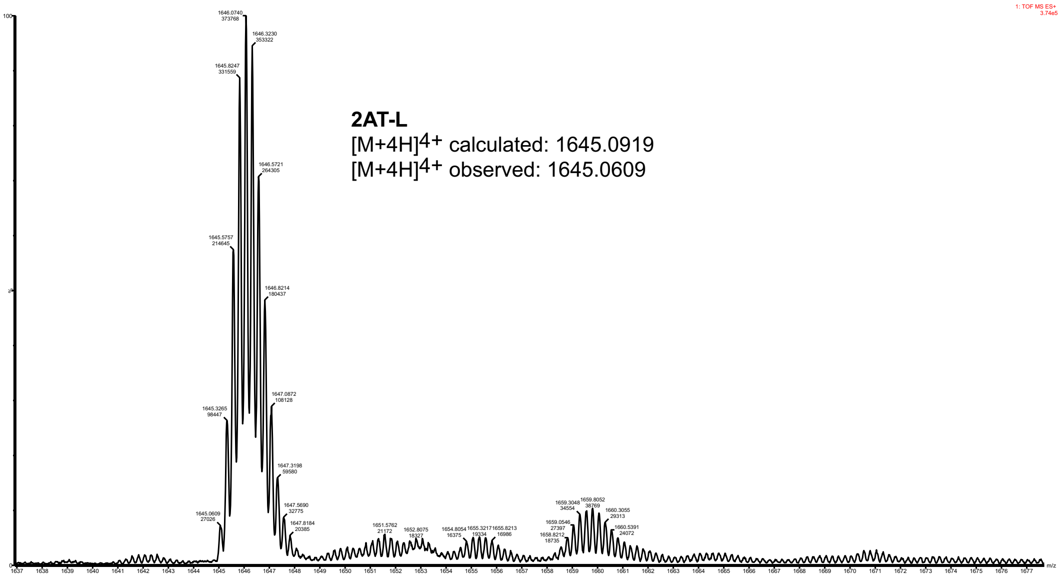
2AT-L
[M+4H]4+ calculated: 1645.0919
[M+4H]4+ observed: 1645.0609

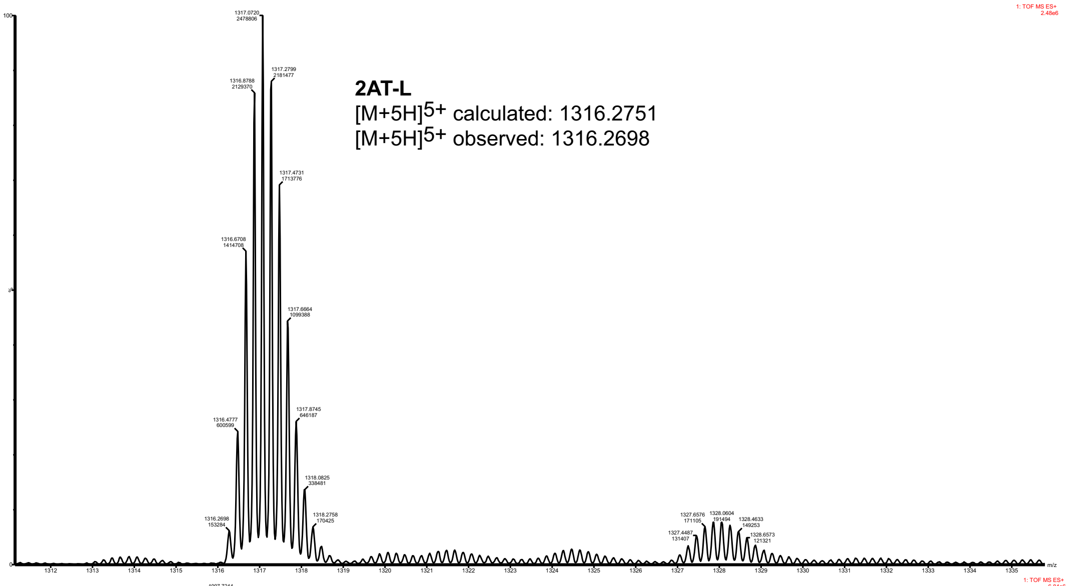
2AT-L
[M+5H]5+ calculated: 1316.2751
[M+5H]5+ observed: 1316.2698

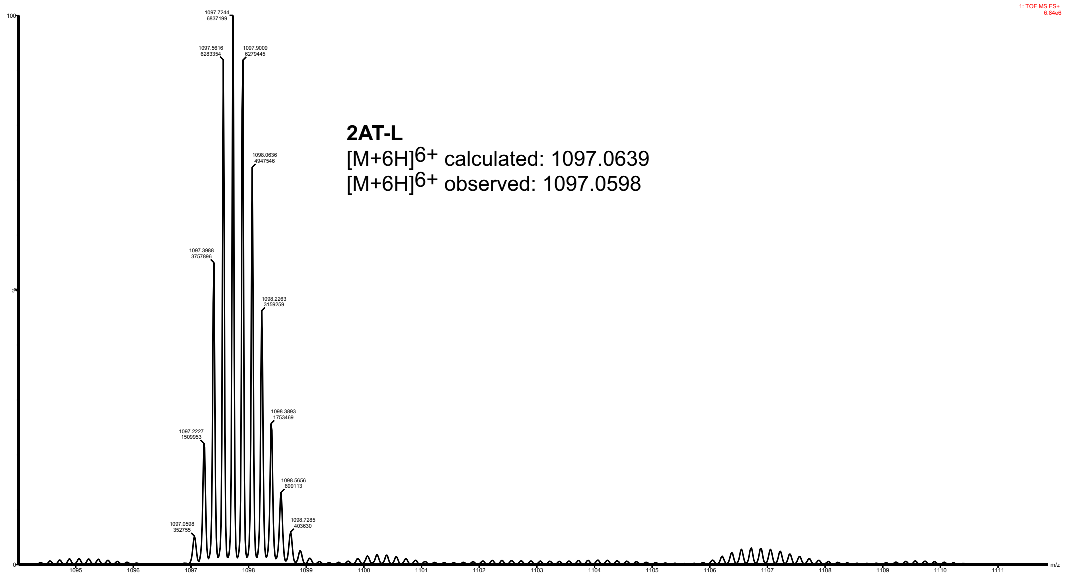
2AT-L
[M+6H]6+ calculated: 1097.0639
[M+6H]6+ observed: 1097.0598

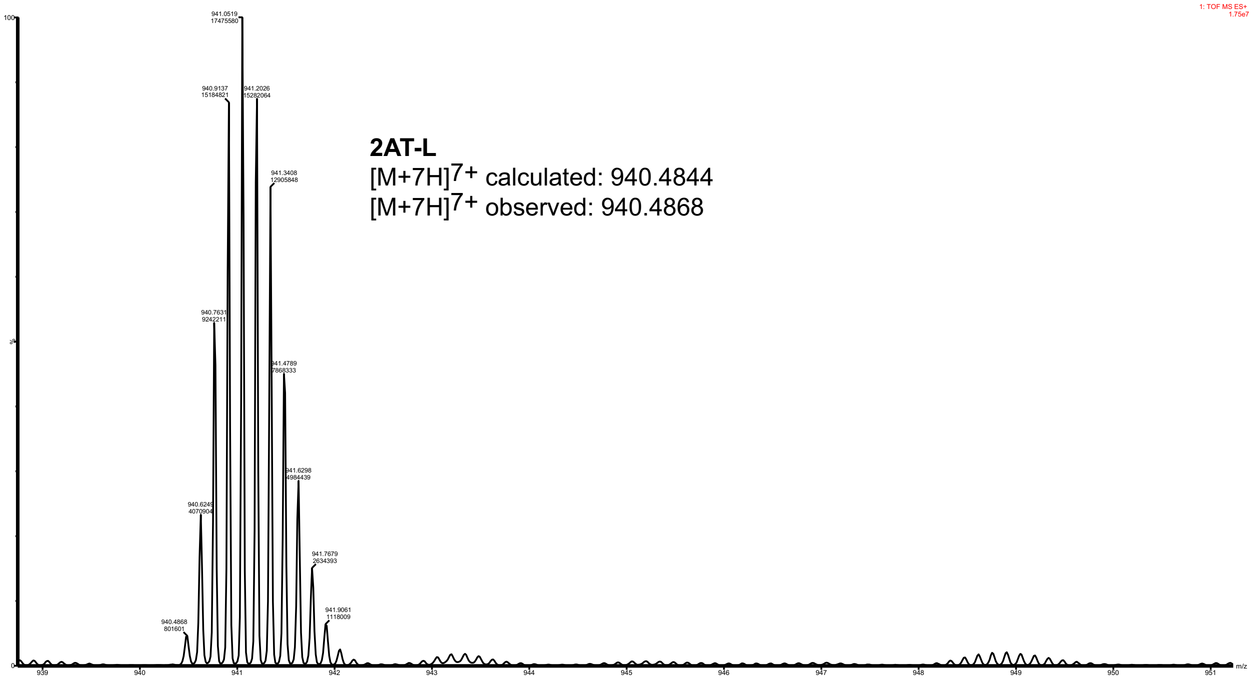
2AT-L
[M+7H]7+ calculated: 940.4844
[M+7H]7+ observed: 940.4868
1: TOF MS ES+
1.75e7
m/z

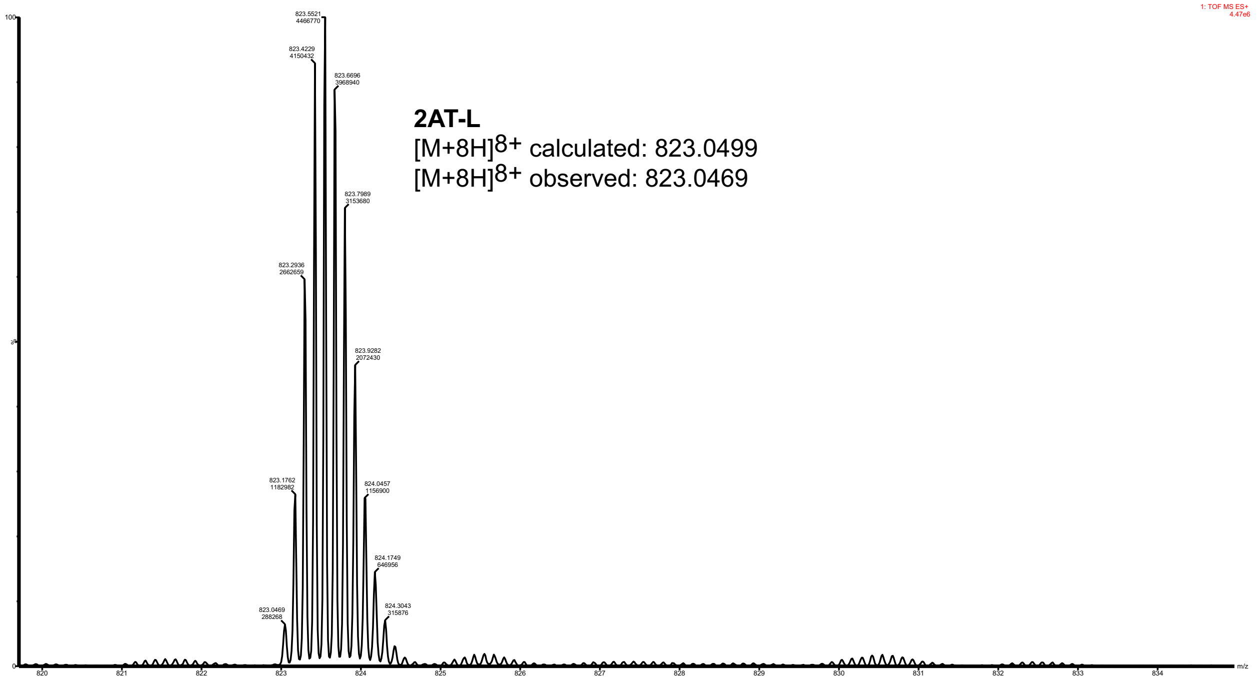
2AT-L
[M+8H]8+ calculated: 823.0499
[M+8H]8+ observed: 823.0469
1: TOF MS ES+
4.47e6
m/z
